# Supplementary material for: The burden of stroke and modifiable risk factors in Ethiopia: A systemic review and meta-analysis
Source: PLoS One. 2021 Nov 1;16(11):e0259244. doi: 10.1371/journal.pone.0259244 (PMC8559958; doi:10.1371/journal.pone.0259244)
Supplement: S1 File — Forest plot of in the proportion of stroke among female and male in Ethiopia, 2020. (DOCX) [file pone.0259244.s002.docx]

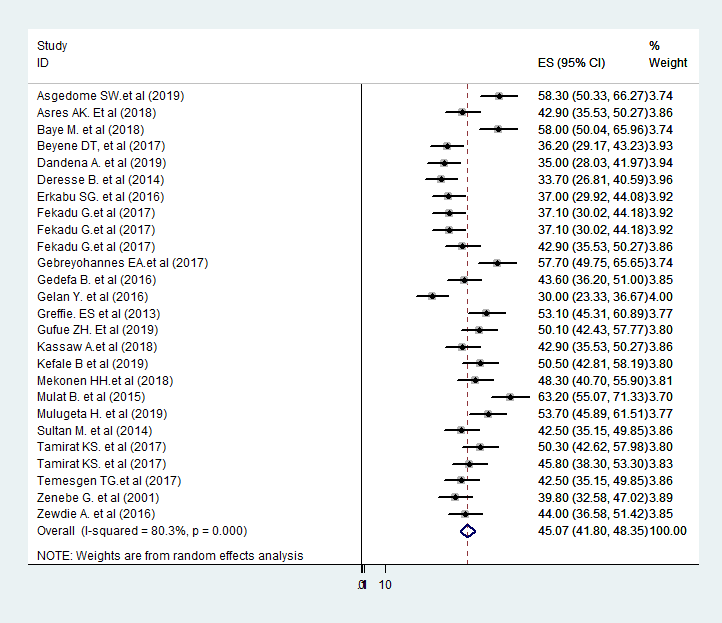


Figure 1 Forest plot of in the proportion of stroke among female in Ethiopia, 2020.


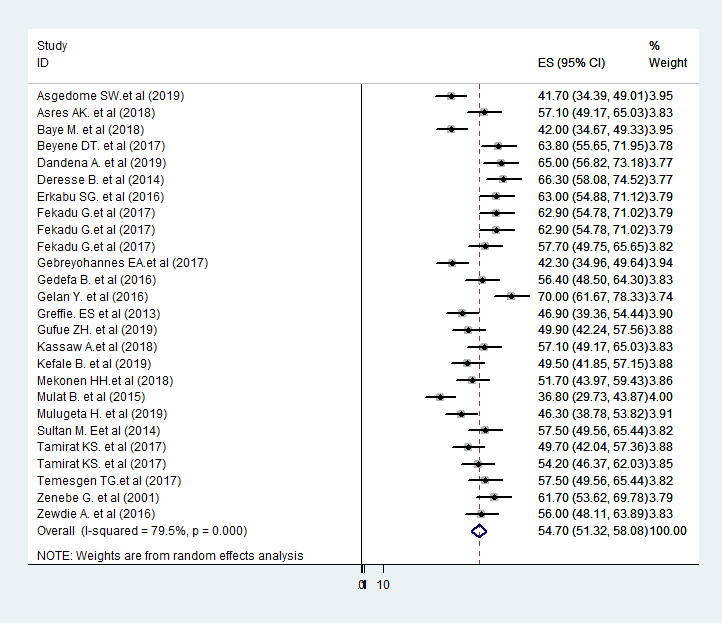


Figure 2 Forest plot of in the proportion of stroke among male in Ethiopia, 2020.
