# Supplementary material for: The burden of stroke and modifiable risk factors in Ethiopia: A systemic review and meta-analysis
Source: PLoS One. 2021 Nov 1;16(11):e0259244. doi: 10.1371/journal.pone.0259244 (PMC8559958; doi:10.1371/journal.pone.0259244)
Supplement: S1 Table — (DOCX) [file pone.0259244.s003.docx]

**Supporting information 1:** search strategy applied to PubMed database in the current review

| Search # | Query | Items found |
| --- | --- | --- |
| 1 | Stroke [Title] OR “Ischemic stroke”[Title] OR “Ischaemic stroke”[Title] OR “Haemorrhagic stroke”[Title] OR “Hemorrhagic stroke” OR “Cerebral Vascular accident” OR CVA | 354,311 |
| 2 | Adults OR “18 years or older” | 985,567 |
| 3 | Ethiopia | 19,558 |
| 4 | Hypertension OR “High blood pressure” [Text Word] OR Diabetes [Text Word] OR “Diabetes mellitus” OR “Smoking” OR “Obesity” OR Alcohol OR “Heavy drinking” [Text Word] OR Physical exercise OR “Physical activity” [Text Word] OR (High blood cholesterol level OR “Hypercholesterolemia, OR Hyperlipidemia” OR “Hyperlipoproteinemia” OR “Arterial fibrillation” | 1,883,137 |
| 5 | #1 AND #2 AND #3 AND #4 | 183 |
