# Supplementary material for: The burden of stroke and modifiable risk factors in Ethiopia: A systemic review and meta-analysis
Source: PLoS One. 2021 Nov 1;16(11):e0259244. doi: 10.1371/journal.pone.0259244 (PMC8559958; doi:10.1371/journal.pone.0259244)
Supplement: S2 Table — (DOCX) [file pone.0259244.s004.docx]

| **S2 Table. Risk of Bias assessment Tool of Eligible Articles by using the Hoy 2012 tool** | | | | | | | | | | | |
| --- | --- | --- | --- | --- | --- | --- | --- | --- | --- | --- | --- |
| **Study ID** | **Representation** | **Sampling** | **Random selection** | **Non response bias** | **Data collection** | **Case Definition** | **Reliability and validity of study tool** | **Method of data collection** | **Prevalence period** | **Numerator and denominator** | **Summary Assessment** |
| Temesgen TG.et al | High risk | Low risk | High risk | Low risk | Low risk | High risk | High risk | High risk | low risk | Low risk | High risk |
| Beyene DT. et al | High risk | Low risk | High risk | Low risk | Low risk | High risk | High risk | High risk | low risk | Low risk | High risk |
| Kefale B. et al | Low risk | Low risk | Low risk | Low risk | Low risk | High risk | Low risk | Low risk | Low risk | Low risk | Low risk |
| Erkabu SG. Et al | Low risk | Low risk | Low risk | Low risk | Low risk | High risk | Low risk | Low risk | Low risk | Low risk | Low risk |
| Deresse B. et al | Low risk | Low risk | High risk | Low risk | High risk | High risk | High risk | Low risk | Low risk | Low risk | High risk |
| Fekadu G.et al | High risk | Low risk | Low risk | Low risk | Low risk | Low risk | Low risk | Low risk | Low risk | Low risk | Low risk |
| Gebreyohannes EA.etal | High risk | Low risk | High risk | Low risk | High risk | Low risk | High risk | Low risk | Low risk | Low risk | Medium risk |
| Gedefa B. et al | High risk | Low risk | Low risk | Low risk | Low risk | Low risk | Low risk | Low risk | Low risk | Low risk | Low risk |
| Greffie. ES et al | High risk | Low risk | Low risk | Low risk | Low risk | Low risk | Low risk | Low risk | Low risk | Low risk | Low risk |
| Kassaw A.et al | High risk | High risk | Low risk | Low risk | Low risk | Low risk | Low risk | Low risk | Low risk | Low risk | Low risk |
| Sultan M. Eet al | Low risk | Low risk | Low risk | Low risk | Low risk | Low risk | Low risk | Low risk | Low risk | Low risk | Low risk |
| Zewdie A. et al | High risk | Low risk | Low risk | Low risk | Low risk | High risk | Low risk | Low risk | Low risk | Low risk | Low risk |
| Mekonen HH.et al | High risk | Low risk | Low risk | High risk | High risk | High risk | High risk | Low risk | High risk | Low risk | High risk |
| Baye M. et al | High risk | Low risk | Low risk | High risk | Low risk | Low risk | Low risk | Low risk | Low risk | Low risk | Low risk |
| Mulugeta H. et al | Low risk | Low risk | Low risk | Low risk | Low risk | Low risk | Low risk | Low risk | Low risk | Low risk | Low risk |
| Mulat B. et al | Low risk | Low risk | Low risk | Low risk | Low risk | High risk | Low risk | Low risk | Low risk | Low risk | Low risk |
| Gufue ZH. Et al | Low risk | Low risk | Low risk | Low risk | Low risk | Low risk | Low risk | Low risk | Low risk | Low risk | Low risk |
| Asres AK. Et al | Low risk | Low risk | Low risk | Low risk | Low risk | Low risk | Low risk | Low risk | Low risk | Low risk | Low risk |
| Bedassa T. et al | High risk | Low risk | Low risk | High risk | Low risk | High risk | Low risk | Low risk | Low risk | Low risk | Medium risk |
| Dandena A. et al | High risk | Low risk | Low risk | Low risk | Low risk | Low risk | Low risk | Low risk | Low risk | Low risk | Low risk |
| Fekadu G.et al | High risk | High risk | High risk | Low risk | Low risk | Low risk | Low risk | Low risk | Low risk | Low risk | Low risk |
| Tamirat KS. et al | Low risk | Low risk | Low risk | Low risk | Low risk | Low risk | Low risk | Low risk | Low risk | Low risk | Low risk |
| Gebremariam SA. et al | High risk | Low risk | Low risk | Low risk | Low risk | High risk | Low risk | Low risk | Low risk | Low risk | Low risk |
| Asgedome SW.et al | Low risk | Low risk | Low risk | Low risk | Low risk | Low risk | Low risk | Low risk | Low risk | Low risk | Low risk |
| Fekadu G.et al | Low risk | Low risk | Low risk | Low risk | Low risk | High risk | Low risk | Low risk | Low risk | Low risk | Low risk |
| Gelan Y. et al | Low risk | Low risk | Low risk | Low risk | Low risk | Low risk | Low risk | Low risk | Low risk | Low risk | Low risk |
| Zenebe G. et al | Low risk | Low risk | Low risk | Low risk | Low risk | Low risk | Low risk | Low risk | Low risk | Low risk | Low risk |
| **Risk of bias assessment tool: Yes (low risk); No (high risk)** | | | | | | | | | | |  |
| 1. Representation: Was the study population a close representation of the national population? | | | | | | | | | | |  |
| 2. Sampling: Was the sampling frame a true or close representation of the target population? | | | | | | | | | | |  |
| 3. Random selection: Was some form of random selection used to select the sample OR was a census undertaken? | | | | | | | | | | |  |
| 4. Non-response bias: Was the likelihood of non-response bias minimal? | | | | | | | | | | |  |
| 5. Data collection: Were data collected directly from the subjects? | | | | | | | | | | |  |
| 6. Case definition: Was an acceptable case definition used in the study? | | | | | | | | | | |  |
| 7. Reliability and validity of study tool: Was the study instrument that measured the parameter of interest show to have reliability and validity? | | | | | | | | | | |  |
| 8. Data collection: Was the same mode of data collection used for all subjects? | | | | | | | | | | |  |
| 9. Prevalence period: Was the length of the prevalence period for the parameter of interest appropriate? | | | | | | | | | | |  |
| 10. Numerators and denominators: Were the numerator(s) and denominator(s) for the parameter of interest appropriate? | | | | | | | | | | |  |
| **The overall risk of bias scored based on the number of high risk of bias per study: low risk (≤2), moderate risk (3–4), and high risk (≥5).** | | | | | | | | | | |  |
|  |  |  |  |  |  |  |  |  |  |  |  |
