# Supplementary material for: The burden of stroke and modifiable risk factors in Ethiopia: A systemic review and meta-analysis
Source: PLoS One. 2021 Nov 1;16(11):e0259244. doi: 10.1371/journal.pone.0259244 (PMC8559958; doi:10.1371/journal.pone.0259244)
Supplement: Attachment — Submitted filename: PO Review_IS.pdf [file pone.0259244.s007.pdf]

PLOS ONE

# Patient recovery from stroke and modifiable risk factors in Ethiopia: a systemic review and meta-analysis.

--Manuscript Draft--

| | |
|---|---|
| **Manuscript Number:** | PONE-D-21-01404 |
| **Article Type:** | Research Article |
| **Full Title:** | Patient recovery from stroke and modifiable risk factors in Ethiopia: a systemic review and meta-analysis. |
| **Short Title:** | Patient recovery from stroke and modifiable risk factors in Ethiopia |
| **Corresponding Author:** | Teshager Wldegiorgis Abate<br>Bahir Dar University<br>Bahir Dar, ETHIOPIA |
| **Keywords:** | Stroke; Modifiable risk factors; Stroke recovery rate; Meta-Analysis; Ethiopia |
| **Abstract:** | Background<br><br>The contribution of modifiable risk factors to the increasing global and regional burden of stroke is unclear, but knowledge about this contribution is crucial for informing stroke prevention strategies. In recent decades, the issue of modifiable risk factors and stroke recovery is one of the keys to improving the quality of life. Modify the risk factor is a strong recommendation to prevent stroke. So far, many studies were conducted to estimate the proportion recovery of stroke and modifiable risk factors in Ethiopia, the lack of a nationwide study that determines the overall proportion of modifiable risk factors and recovery of stroke is an important research gap.<br><br>Methods<br><br>This study was conducted following the PRISMA checklist. Three reviewers have been searched and extracted from the CINAHL (EBSCO), MEDLINE (via Ovid), PubMed, EMcare, and Google scholar. Articles' quality was assessed using the Newcastle-Ottawa Scale by two independent reviewers, and only studies with low and moderate risk were included in the final analysis. The review presented the pooled proportion of stroke recovery and modifiable risk factors of stroke in people with stroke. The review has been registered in PROSPERO with protocol number CRD42020221906.<br><br>Results<br><br>In this study, t he pooled proportion stroke recovery in Ethiopia was 72.28% (95%CI: 62.48, 82.08; I2 = 96.5%). The overall magnitude modifiable risk factor of hypertension, alcohol consumption and dyslipidemia among people with stroke were 49% (95%CI: 43.59, 54.41), 24.96% (95CI%:15.01, 34.90), and 20.99% (95%CI: 11.10, 30.88), respectively. The least proportion of stroke recovery was in the Oromia region (67.38 (95%CI: 41.60-93.17; I2 = 98.1%). Besides, the proportion of stroke recovery was decreased after 2017 (70.50 (56.80 - 84.20).<br><br>Conclusions<br><br>Even though our pooled result showed that stroke recovery is in line with the ideal proportional recovery rule of stroke, this not satisfactory in the comparison of other developing countries. More than 90% of patients had one or more modifiable risk factors. All modifiable stroke risk factors, especially high blood pressure and alcohol consumption, should be understood as a major public health problem in Ethiopia and efforts should be focused on the primary prevention of stroke. |
| **Order of Authors:** | Teshager Wldegiorgis Abate |
| | Balew Zeleke |
| | Ashenafi Genanew |
| | |

Powered by Editorial Manager® and ProduXion Manager® from Aries Systems Corporation

| | Bidiru Weldegiorgis Abate |
|---|---|
| **Additional Information:** | |
| **Question** | **Response** |
| **Financial Disclosure**<br><br>Enter a financial disclosure statement that describes the sources of funding for the work included in this submission. Review the submission guidelines for detailed requirements. View published research articles from *PLOS ONE* for specific examples.<br><br>This statement is required for submission and **will appear in the published article** if the submission is accepted. Please make sure it is accurate.<br><br>**Unfunded studies**<br>Enter: *The author(s) received no specific funding for this work.*<br><br>**Funded studies**<br>Enter a statement with the following details:<br>• Initials of the authors who received each award<br>• Grant numbers awarded to each author<br>• The full name of each funder<br>• URL of each funder website<br>• Did the sponsors or funders play any role in the study design, data collection and analysis, decision to publish, or preparation of the manuscript?<br>• **NO** - Include this sentence at the end of your statement: *The funders had no role in study design, data collection and analysis, decision to publish, or preparation of the manuscript.*<br>• **YES** - Specify the role(s) played.<br><br>* typeset | NO - Include this sentence at the end of your statement: The funders had no role in study design, data collection and analysis, decision to publish, or preparation of the manuscript. |
| **Competing Interests**<br><br>Use the instructions below to enter a competing interest statement for this submission. On behalf of all authors, disclose any competing interests that could be perceived to bias this work—acknowledging all financial support and any other relevant financial or non-financial competing interests. | NO authors have competing interests |

| | |
|---|---|
| This statement is required for submission and **will appear in the published article** if the submission is accepted. Please make sure it is accurate and that any funding sources listed in your Funding Information later in the submission form are also declared in your Financial Disclosure statement.<br><br>View published research articles from *PLOS ONE* for specific examples.<br><br>**NO authors have competing interests**<br><br>Enter: *The authors have declared that no competing interests exist.*<br><br>**Authors with competing interests**<br><br>Enter competing interest details beginning with this statement:<br><br>*I have read the journal's policy and the authors of this manuscript have the following competing interests: [insert competing interests here]*<br><br>* typeset | |
| **Ethics Statement**<br><br>Enter an ethics statement for this submission. This statement is required if the study involved:<br><br>• Human participants<br>• Human specimens or tissue<br>• Vertebrate animals or cephalopods<br>• Vertebrate embryos or tissues<br>• Field research<br><br>Write "N/A" if the submission does not require an ethics statement.<br><br>General guidance is provided below. Consult the submission guidelines for detailed instructions. **Make sure that all** | N/A |

| | |
|---|---|
| **information entered here is included in the Methods section of the manuscript.**<br><br>**Format for specific study types**<br><br>**Human Subject Research (involving human participants and/or tissue)**<br>• Give the name of the institutional review board or ethics committee that approved the study<br>• Include the approval number and/or a statement indicating approval of this research<br>• Indicate the form of consent obtained (written/oral) or the reason that consent was not obtained (e.g. the data were analyzed anonymously)<br><br>**Animal Research (involving vertebrate animals, embryos or tissues)**<br>• Provide the name of the Institutional Animal Care and Use Committee (IACUC) or other relevant ethics board that reviewed the study protocol, and indicate whether they approved this research or granted a formal waiver of ethical approval<br>• Include an approval number if one was obtained<br>• If the study involved *non-human primates*, add *additional details* about animal welfare and steps taken to ameliorate suffering<br>• If anesthesia, euthanasia, or any kind of animal sacrifice is part of the study, include briefly which substances and/or methods were applied<br><br>**Field Research**<br><br>Include the following details if this study involves the collection of plant, animal, or other materials from a natural setting:<br>• Field permit number<br>• Name of the institution or relevant body that granted permission | |
| **Data Availability**<br><br>Authors are required to make all data underlying the findings described fully available, without restriction, and from the time of publication. PLOS allows rare exceptions to address legal and ethical | Yes - all data are fully available without restriction |

| | |
|---|---|
| concerns. See the PLOS Data Policy and FAQ for detailed information.<br><br>A Data Availability Statement describing where the data can be found is required at submission. Your answers to this question constitute the Data Availability Statement and **will be published in the article**, if accepted.<br><br>**Important:** Stating 'data available on request from the author' is not sufficient. If your data are only available upon request, select 'No' for the first question and explain your exceptional situation in the text box.<br><br>Do the authors confirm that all data underlying the findings described in their manuscript are fully available without restriction? | |
| **Describe where the data may be found in full sentences. If you are copying our sample text, replace any instances of XXX with the appropriate details.**<br><br>• If the data are **held or will be held in a public repository**, include URLs, accession numbers or DOIs. If this information will only be available after acceptance, indicate this by ticking the box below. For example: *All XXX files are available from the XXX database (accession number(s) XXX, XXX.*).<br>• If the data are all contained **within the manuscript and/or Supporting Information files**, enter the following: *All relevant data are within the manuscript and its Supporting Information files.*<br>• If neither of these applies but you are able to provide **details of access elsewhere**, with or without limitations, please do so. For example:<br><br>*Data cannot be shared publicly because of [XXX]. Data are available from the XXX Institutional Data Access / Ethics Committee (contact via XXX) for researchers who meet the criteria for access to confidential data.* | All relevant data are within the manuscript and its Supporting Information files. |

| | |
|---|---|
| *The data underlying the results presented in the study are available from (include the name of the third party and contact information or URL).*<br>• This text is appropriate if the data are owned by a third party and authors do not have permission to share the data.<br><br>* typeset | |
| Additional data availability information: | |

Powered by Editorial Manager® and ProduXion Manager® from Aries Systems Corporation

Manuscript Click here to access/download;Manuscript;001 munuscript of stroke current plos one.docx

# Patient recovery from stroke and modifiable risk factors in Ethiopia: a systemic review and meta-analysis.

Teshager Weldegiorgis Abate[1]*, Balew Zeleke[2&], Ashenafi Genanew[3 ¶], and Bidiru Weldegiorgis Abate[4 ¶]

[1]Department of adult health Nursing, School of Health Science, College of Medicine and Health Science, Bahir Dar University, P.O. Box 79, Bahir Dar, Ethiopia.

[2]Department of Pediatric and Child Health Nursing, School of Health Sciences, College of Medicine and Health Sciences, Bahir Dar University, P.O. Box 79, Bahir Dar, Ethiopia.

[3]Department of pharmacology, School of Health Sciences, College of Medicine and Health Science, Bahir Dar University, P.O. Box 79, Bahir Dar, Ethiopia.

[4]Department of Anesthetics, Addis Alem Hospitla, Amhara Regional Bureau

*Corresponding author: teshagerhylemarriam@gmail.com, phone number +251912886162

[¶]These authors contributed equally to this work.

[&]This authors also contributed equally to this work

# Abstract

**Background:** The contribution of modifiable risk factors to the increasing global and regional burden of stroke is unclear, but knowledge about this contribution is crucial for informing stroke prevention strategies. In recent decades, the issue of modifiable risk factors and stroke recovery is one of the keys to improving the quality of life. Modify the risk factor is a strong recommendation to prevent stroke. So far, many studies were conducted to estimate the proportion recovery of stroke and modifiable risk factors in Ethiopia, the lack of a nationwide study that determines the overall proportion of modifiable risk factors and recovery of stroke is an important research gap**.**

**Methods**: This study was conducted following the PRISMA checklist. Three reviewers have been searched and extracted from the CINAHL (EBSCO), MEDLINE (via Ovid), PubMed, EMcare, and Google scholar. Articles' quality was assessed using the Newcastle-Ottawa Scale by two independent reviewers, and only studies with low and moderate risk were included in the final analysis. The review presented the pooled proportion of stroke recovery and modifiable risk factors of stroke in people with stroke. The review has been registered in PROSPERO with protocol number CRD42020221906.

**Results:** In this study, **t**he pooled proportion stroke recovery in Ethiopia was 72.28% (95%CI: 62.48, 82.08; I2 = 96.5%). The overall magnitude modifiable risk factor of hypertension, alcohol consumption and dyslipidemia among people with stroke were 49% (95%CI: 43.59, 54.41), 24.96% (95CI%:15.01, 34.90), and 20.99% (95%CI: 11.10, 30.88), respectively. The least proportion of stroke recovery was in the Oromia region (67.38 (95%CI: 41.60-93.17; I2 = 98.1%). Besides, the proportion of stroke recovery was decreased after 2017 (70.50 (56.80 - 84.20).

**Conclusions:** Even though our pooled result showed that stroke recovery is in line with the ideal proportional recovery rule of stroke, this not satisfactory in the comparison of other developing countries. More than 90% of patients had one or more modifiable risk factors. All modifiable stroke risk factors, especially high blood pressure and alcohol consumption, should be understood as a major public health problem in Ethiopia and efforts should be focused on the primary prevention of stroke.

**Keywords:** Stroke, Modifiable risk factors, Stroke recovery rate, Meta-analysis, Ethiopia

# Background

Stroke remains the second leading cause of death worldwide, with an annual mortality rate of 5·5 million. Stroke has increased across the globe in both men and women of all ages. Fewer women (2·6 million) than men (2·9 million) died from stroke (1, 2). The incidence, prevalence, and mortality rate of stroke have ~~been~~ increased worldwide, with most of the burden being in the low and middle-income country including Ethiopia (3, 4). Ischemic stroke is more frequent, but hemorrhagic stroke is responsible for more deaths, and Disability-Adjusted Life-Years (DALYs) ~~are~~ lost. Incidence and mortality of stroke differ between countries, geographical regions, and ethnic groups (5).

Ethiopia faces the unenviable threat of a triple burden of disease: infectious or communicable diseases, Non-Communicable Diseases (NCDs), and injuries are steadily increasing and pose risks to the lives of Ethiopians (6). Although Ethiopia is progressing towards universal health coverage, the country faces the double burden of both communicable and NCD (7). The magnitude of stroke deaths in Ethiopia was 6.23% of total deaths, and the age-adjusted death rate of stroke in the country is 89.82 per 100 000 of the population (8). Besides, previous reports indicated that 90%

of the burdens of stroke are attributable to modifiable risk factors (9). Of this, three-quarters of the stroke burden is attributable to behavioral risk factors (10).

Most of the stroke burden was attributable to modifiable risks. For example, metabolic factors (high blood pressure, obesity, fasting plasma glucose, cardiac disorder, and total cholesterol) accounted for 72% of stroke DALYs, behavioral factors (smoking, poor diet, and physical inactivity) accounted for 66%, and environmental risks (air pollution and lead exposure) accounted for about 28%. Although most of the risk attribution information is not novel, many of these risks are modifiable. They have been shown to reduce stroke and important to track and emphasis (5, 11-15).

Though there were many primary studies conducted to estimate the magnitude of subtypes of stroke, modifiable risk of stroke, and rate of improvement at discharge after stroke in Ethiopia, informative and comprehensive reviews are lacking, and the magnitude of subtypes of stroke, modifiable risk of stroke, and rate of improvement at discharge after stroke is an important research gap. Meta-analysis is key to improve the accuracy of estimates through the use of more data sets. Thus, this study was aimed to determine the overall magnitude subtypes of stroke, modifiable risk factors of stroke, and rate improvement after stroke in Ethiopia.

# Methods and analysis

## Protocol design and registration

A systematic review and meta-analysis of observational studies published and unpublished research articles that were conducted to assess the magnitude of ischemic and hemorrhagic stroke and the rate of improvement at discharge in Ethiopia were included during this study. The Preferred Reporting Items for Systematic Review and Meta-analysis Protocol (PRISMA-P) (16, 17) and Meta-analysis of Observational Studies in Epidemiology (MOOSE) guideline statement (18) were

used for the development of this study protocol, the conduct and design, and the reporting of these results. To minimize duplication of the same review, provide transparency, and to reduce reporting bias of the current review, an intensive search was done before data extraction. ~~A~~ protocol registered in the International Registration of Systems Reviews (PROSPERO) (registration number CRD42020221906).

## Eligibility Criteria

The eligibility of the study was determined using the following criteria: (1) all facility-based observational studies; (2) all studies conducted in Ethiopia; (3) all studies reporting either the magnitude of any subtypes of stroke or rate of improvement at discharge ~~and~~ modifiable risk factors; and both published and unpublished studies. On the other hand, anonymous report, case report, qualitative, and research which were unable to access the full-text after three email contacts of principal investigators of the particular studies were excluded from the analysis.

## Information source and search strategies

Standardized and well-described methods were used in this systemic review (16). Briefly, a search strategy was developed after the identification of the relevant Medical Subject Headings (MESH), keywords, and their synonyms. The search strategy for Pumed: the key words which we used in our search included terms describing stroke, terms describing age, and term describing modifiable risk factors as shown in the search strategy as follows: (1) (Stroke [Title] OR “Ischemic stroke”[Title] OR “Ischaemic stroke”[Title] OR “Haemorrhagic stroke”[Title] OR “Hemorrhagic stroke” OR “Cerebral Vascular accident” OR CVA); (2) (Adults OR “18 years or older”) [Text Word] (3) (Ethiopia) [Text Word] **(**4**)** (Hypertension OR “High blood pressure” [Text Word] OR Diabetes [Text Word] OR “Diabetes mellitus” OR “Smoking” OR “Obesity” OR Alcohol OR “Heavy drinking” [Text Word] OR Physical exercise OR “Physical activity” [Text Word] OR (High

blood cholesterol level OR “Hypercholesterolemia, Hyperlipidemia” OR “Hyperlipoproteinemia” OR “Arterial fibrillation) [Text Word] (5) #1 AND #2 AND #3 AND #4 (S1.).

A pretest of the search strategy by two authors was performed in PubMed, and the actual electronic search was done from November 20 to 25, 2020. Two independent reviewers implemented the electronic search in the following electronic databases: CINAHL (EBSCO), MEDLINE (via Ovid), PubMed, EMcare, and Google scholar search engines. Moreover, snowballing and retrieving references or hand searching was performed on the reference lists of eligible studies to include studies that unable to identify by the search strategy. Finally, the search process was presented in a PRISMA flow chart.

## Study Selection

Two of the reviewers (TWA and BWA) screened the titles and abstracts of each article to find potentially eligible studies. After removing duplicates, search results were exported to End-Note software (version X7 Thomson Reuters, New York, NY) used to create a bibliographical database of the retrieved references. The selection process was conducted in two stages: first screening of titles and abstracts against the predetermined inclusion/exclusion criteria, followed by a second screening of the full text of the research reports identified as probably relevant in the initial screening. Both stages were carried out independently by two authors (TWA and AG), and disagreements resolved by discussion with another author (BWA) determined the last eligibility of the included articles. Articles that fulfilled the earlier criteria were used as a source of data for analysis.

## Data Extraction process and Quality Assessment

The abstract and full-text review data abstraction was done by three independent authors (TWA, BZ, and AG) using a pre-piloted data extraction format prepared in the Microsoft$^{TM}$ Excel

spreadsheet. Disagreement in data abstraction between the first two and third authors was resolved by a fourth independent author (BWA). From each primary study, we have extracted data participant sex, study year, region, sample size, study design, and first author name for the proportion of ischemic stroke, hemorrhagic stroke, improvement at discharge, and each risk factors (hypertension, diabetes mellitus, alcohol consumption, smoking, heart disease, lack physical activities, cholesterol, and obesity).

Before analysis, a transformation of prevalence was made. The Newcastle-Ottawa Scale (NOS) was used to select for assessing the quality of the included studies. The NOS included three categorical criteria with a maximum score of ten points. The assessment tool contains representatives of the sample, sample size, non-respondents, and ascertainment of exposure, independent blind assessment, and statistical test. Based on this tool, a primary study with a score of 6 out of 10 was considered as good quality. In the review that to maintain the validity of this review, we only included primary studies with fair to good quality (17-19).

The primary outcome of this review was the pooled proportion of the overall ischemic, hemorrhagic, and survival rate discharge, and the magnitude of common modifiable risk factors among people with stroke in Ethiopia. Stroke was defined as rapidly developing clinical signs of focal, or at times, global disturbance of cerebral function, lasting more than 24 hours or leading to death with no apparent cause other than a vascular origin (20, 21).

## Quality Assessment

The risk of bias of included studies is assessed using the 10-item rating scale developed by Hoy et al. for prevalence studies (22). The assessment tool has a representative sample size, method of data collection, reliability and validity of study tools, case definition, and prevalence periods of the studies. Researchers categorized each study as having a low risk of bias ("yes" answers to

domain questions) or a high risk of bias ("no" answers to domain questions). Each study assigned a score of 1 (Yes) or 0 (No) for each domain, and these domain scores were summed to give an overall study quality score. Scores of 8–10 were considered as having a "low risk of bias", 6–7 a "moderate risk", and 0–5 a "high risk". For the least risk of bias classification, discrepancies between the reviewers resolved via consensus.

# Data analysis

## Heterogeneity test and publication bias

Heterogeneity between the findings of the primary studies was assessed by using Cochran's Q test and quantified with the I-square statistics. A P-value of less than 0.1 was considered as ~~suggest~~ statistically significant heterogeneity, considering a small number of studies and their heterogeneity in design (23). Heterogeneity ~~had taken low, moderate, and high classifications~~ when I-square values were below 25%, between 25 and 75%, and above 75% respectively (24). The random-effect model was used to pool the recommended health life rate. The random effect model accounts for heterogeneity among study results beyond the variation associated with the chance unlike the fixed effect model (25).

To investigate the source of heterogeneity, the random-effect model was used. The meta-analysis was weighted to account for the residual between-study heterogeneity (i.e., heterogeneity not explained by the covariate in the regression (26). Publication bias was assessed by visual inspection of funnel plots based on the shape of the graph. The symmetrical graph was interpreted as an absence of publication bias. An asymmetrical one indicated the presence of publication bias. On the other hand, Begg's and Egger's weighted regression was employed to identify the source of publication bias. P-values less than 0.05 were considered as the presence of significant publication bias (27, 28). A leave-out sensitivity analysis performed to confirm some studies

potentially biased the direction of the pooled estimate. Subgroup analyses by region and type of study setup (hospitals) were carried out because of significant heterogeneity between studies (i.e., $I^2 = 96.5\%$, $p<0.05$)

### Statistical analysis

Data were analyzed in Stata Version 14. Data was presented in the evidence table and summarized using descriptive statistics. The effect measure for outcome variables were computed using the "Metaprop" command for meta-analysis of the proportion in Stata. In this review, the overall burden of ischemic stroke, hemorrhagic stroke, rate of improvement, and common modifiable risk factors was calculated together with their corresponding 95% CI. A forest plot was generated to show the individual pooled burden of strokes and common modifiable stroke 95% CI, the author's name, study year, and study weights.

# Result

## Study selection process

From electronic databases, we retrieved 986 observational studies. After screening their titles and abstracts, duplications were removed using Endnote X7. Accordingly, 644 duplicate articles were removed. From 342 articles, 315 articles were excluded because their titles and abstracts were not in-line with our inclusion criteria (full article not found, different population, different setting, and different outcome). Finally, 27 articles were included for this systemic review and meta-analysis (Fig. 1).

## Study characteristics

Overall, a total of 27 observational studies were selected in this systematic review and meta-analysis. This consisted of 5,845 participants. Among them, 2,647 participants were male, and

3,228 participants were improved at the time of discharge. The number of participants in each study ranged from 73 to 503. The most retrieved studies (n = 8) were from the Oromia (29-36) region followed by Addis Ababa (n=7) (37-43) and Amhara region (n=7) (44-50), Tigray region represented by four studies (51-54), whereas Southern Nations Nationalities and People's (SNNP) (55) region represented by one study.

The smallest sample size was 73 obtained from a study conducted at Shashemene Referral Hospital, Ethiopia (29). Whereas, the largest sample size was 503 reported from a study done at Ayder Comprehensive Specialized Hospital, Northern Ethiopia (52). Most studies dealt with hypertension as a modifiable risk factors of stroke (n=24) (29-34, 36, 37, 39-41, 43, 44, 48, 51-59) followed by Arterial Fibrillation (AF) (n = 17) (30-32, 37, 40, 41, 44, 45, 47-50, 53-55, 57, 58), Diabetes mellitus (DM) (n = 15) (30, 31, 33, 37, 39-41, 44, 48, 49, 55-59), other heart disease rather than AF (n = 14) (29, 30, 32, 37, 41, 43, 47, 49, 54-59), and high cholesterol level (n = 7) (32, 33, 35, 37, 44, 48, 49, 51, 55) (Table1).

*Table 1: Study characteristics of included articles for the final systematic review and meta-analysis on the burden of modifiable risk factors and rate of improvement at discharge after stroke in Ethiopia 2020*

| Authors name | Study year | Region | Sample size | Study design | Burden of Stroke reported outcome percentage (95% CI) | | | | | |
|---|---|---|---|---|---|---|---|---|---|---|
| | | | | | Ischemic stroke | Hemorrhagic stroke | Female | Male | Improvement at discharge | NOS score |
| Asgedome SW.et al | 2019 | Tigray | 216 | R | 55.6 | 44.4 | 58.3 | 41.7 | 77.8 | 8 |
| Asres AK. et al | 2018 | AA | 170 | CC | 51.2 | 37.6 | 42.9 | 57.1 | 72.4 | 7 |
| Baye M. et al | 2018 | Amhara | 448 | R | 31.5 | 68.5 | 58.0 | 42.0 | 59.8 | 8 |
| Bedassa T. et al | 2018 | Oromia | 242 | R | 64.3 | 35.7 | no | Not | Not | 5 |
| Beyene DT. et al | 2017 | Oromia | 367 | R | 35.7 | 64.31 | 36.2 | 63.8 | 26.4 | 8 |
| Dandena A. et al | 2019 | Oromia | 283 | P | 43.1 | 44.5 | 35.0 | 65.0 | Not | 6 |

| | | | | | | | | | | |
|---|---|---|---|---|---|---|---|---|---|---|
| Deresse B. et al | 2014 | SNNP | 163 | P | 50.3 | 49.7 | 33.7 | 66.3 | 85.3 | 8 |
| Erkabu SG. et al | 2016 | Amhara | 303 | R | 59.4 | 40.6 | 37 | 63.0 | 89.0 | 7 |
| Fekadu G.et al | 2017 | Oromia | 116 | P | 51.7 | 48.3 | 37.1 | 62.9 | Not | 6 |
| Fekadu G.et al | 2017 | Oromia | 116 | CC | 48.3 | 41.6 | 37.1 | 62.9 | 78.4 | 8 |
| Fekadu G.et al | 2017 | Oromia | 364 | CC | 42.3 | 57.7 | 42.9 | 57.7 | 94.0 | 7 |
| Gebremariam SA. et al | 2014 | Tigray | 142 | CC | 55.6 | 38.0 | 45.8 | 54.2 | 47.9 | 8 |
| Gebreyohannes EA.et al | 2017 | Amhara | 208 | R | 57.7 | Not | 57.7 | 42.3 | 87.5 | 7 |
| Gedefa B. et al | 2016 | AA | 163 | R | 35.6 | 64.4 | 43.6 | 56.4 | 69.9 | 8 |
| Gelan Y. et al | 2016 | AA | 227 | CC | 49.8 | 48.9 | 30.0 | 70.0 | 70.0 | 7 |
| Greffie. ES et al | 2013 | Amhara | 98 | R | 69.4 | 30.6 | 53.1 | 46.9 | 87.0 | 7 |
| Gufue ZH. et al | 2019 | Tigray | 503 | R | 56.6 | 43.4 | 50.1 | 49.9 | 85.1 | 7 |
| Kassaw A.et al | 2018 | AA | 170 | R | 51.2 | 48.8 | 42.9 | 57.1 | 80.0 | 8 |
| Kefale B. et al | 2019 | Oromia | 111 | R | 80.1 | 18.0 | 50.5 | 49.5 | 83.8 | 7 |
| Mekonen HH.et al | 2018 | Tigray | 89 | R | 32.6 | 36.6 | 63.2 | 51.7 | Not | 5 |
| Mulat B. et al | 2015 | Amhara | 427 | R | 56.7 | 43.3 | 63.2 | 36.8 | Not | 6 |
| Mulugeta H. et al | 2019 | Amhara | 162 | R | 50.0 | 30.0 | 53.7 | 46.3 | 27.2 | 7 |
| Sultan M. et al | 2014 | AA | 301 | p | 53.8 | 17.9 | 42.5 | 57.5 | 80.7 | 8 |
| Tamirat KS. et al | 2017 | Amhara | 151 | R | 60.3 | 39.7 | 50.3 | 49.7 | 90.7 | 7 |
| Temesgen TG.et al | 2017 | Oromia | 73 | R | 65.8 | 34.2 | 42.5 | 57.5 | 54.8 | 6 |
| Zenebe G. et al | 2001 | AA | 128 | CC | 43 | 57.0 | 39.8 | 61.7 | Not | 6 |
| Zewdie A. et al | 2016 | AA | 104 | CC | 44.2 | 55.8 | 44.0 | 56.0 | Not | 5 |

R: Retrospective, P: Prospective, CC: Cross-Sectional, AA: Addis Ababa, NOS: Newcastle-Ottawa Scale

# Quality Appraisal

The quality score of the included study ranged from 5 to 8 to a mean score of 7.04 (SD = 0.94). Out of 27 studies, 21 (77.78 %) studies received a low risk of bias. The authors also find types of bias: 5 studies (29-31, 36, 39, 44, 49, 51, 55) had a high risk of case definition, 5 studies (29, 30, 35, 55, 56) had a high risk of random selection bias, and 14 studies (29, 33-35, 39, 41, 47, 51, 53, 57, 58) had a high risk of representation bias (S2.)

## Magnitude of strokes in Ethiopia

From the total ranked of twenty seven primary studies, twenty five, twenty six studies provided information on the proportion of hemorrhagic and ischemic stroke respectively. Twenty six primary studies also provide information the proportion of stroke ~~on~~ female and male. Whereas twenty primary studies reported the rate improvement at discharge after stroke. As presented in the forest plot (Fig. 2 and 3), the pooled estimate proportion of hemorrhagic and ischemic stroke were 46.42% (95%CI: 41.82-51.53; $I^2$ = 91.6%) and 51.40% (95%CI: 46.97- 55.82; $I^2$ = 85.5%) respectively. High heterogeneity was observed among the included studies. The pooled estimate of stroke among female was 45.07 % (95%CI: 41.80-48.35; $I^2$ = 80.3%) and male was 54.70 % (95%CI: 51.32-58.08; $I^2$ = 79.5%) (S3.).

## Magnitude of modifiable risk factors of stroke in Ethiopia

We investigated the magnitude of modifiable risk factors of stroke among the included studies. The proportion of DM among stroke patients ranged from 5.2% (54) to 21.6% (39). A total of 3, 356 stroke ~~people~~ were included to determine the pooled magnitude of DM among stroke ~~people.~~ Accordingly, our pooled analysis showed that 14.722% (95%CI: 9.51, 19.94; $I^2$ = 95.8) of stroke people had DM. In this review, stroke people who had hypertension ranged from 24.1% (33) to 75.2% (32). To determine the pooled magnitude of hypertension among stroke patients, a total of 5,064 people with stroke were included. Consequently, we found that the overall magnitude of hypertension among stroke people was 49% (95CI%:43.59, 54.41; $I^2$ = 91.6%). Furthermore, the proportion of prestrike story excessive alcohol consumption among stroke people included in this study ranged from 10.4% (55) to 41.4% (48), and our meta-analysis revealed that 24.96 % (95%CI: 15.01, 34.90; $I^2$ = 92.7) of people with stroke had history of excessive alcohol intake (Table 2).

Table 2: The pooled effect of common modifiable risk factors among the primary studies of stroke in Ethiopia

| Modifiable risk factors | Estimated pooled proportion (95%CI) | I-squared (%) |
|---|---|---|
| Hypertension | 49 (43.59, 54.41) | 91.6 |
| Diabetes mellitus | 14.72 (9.51, 19.94) | 95.8 |
| Atrial fibrillation | 19.21 (13.96, 24.46) | 94.4 |
| Other heart disease | 20.11 (14.27, 25.95) | 94.2 |
| Dyslipidemia | 20.99 (11.10, 30.88) | 96.4 |
| Smoking | 10.38 (6.27, 14.94) | 86.0 |
| Obesity | 11.64 (2.48, 20.79) | 95.3 |
| Alcohol | 24.96 (15.01, 34.90) | 92.7 |

Other heart Disease: Congestive heart failure, Structural heart disease, Myocardia friction

## Recovery from stroke in Ethiopia

Proportion of improvement during discharge after stroke among the included primary studies ~~was~~ ranged from 26.4% (30) to 94% (36). A total of 2321 stroke people were included to determine the pooled magnitude of improvement at the time of discharge among stroke patients, and the pooled improvement status of stroke during discharge in Ethiopia was 72.28% (95%CI: 62.48, 82.08; $I^2$ = 96.5%) (Fig. 4).

## Publication bias

Both funnels plot of precision asymmetry and Egger's test of the intercept showed that there is no publication bias in the primary studies. Visual examination of the funnel plot showed a symmetric distribution of studies. Additionally, Egger's test of the intercept was -0.147 (95% CI: -0.26, 1.18) $p > 0.05$

(0.102), as judged by Egger's test, there was no evidence of publication bias being presented at 5% significance level (Fig. 5).

## Subgroup analysis

Due to the heterogeneity of included studies, further sub-group analysis was done by using the following study characteristics: region, sample size and study year. The random-effect model was applied for reporting the pooled proportion of clinical outcome during discharge of the sub-group analysis. Accordingly, the highest recovery rate was observed from the Addis Ababa region 74.51 (69.84-79.17; $I^2$ = 34.5%). Whereas the least proportion of recovery was in Oromia region (67.38 (95%CI: 41.60-93.17; $I^2$ = 98.1%). The subgroup analysis by study year showed that the proportion of recovery after stroke ~~during~~ discharge was 75.59 % (95%CI 64.28 - 86.9; $I^2$ = 92.1%) for studies conducted before 2017 (Table 3).

Table 3: Subgroup analysis of ~~recover~~ after stroke by region, sample size, and study year in Ethiopia 2020.

| Variables | Characteristics | Estimated stroke recover during discharge (95% CI; $I^2$ = %) |
|---|---|---|
| Region | Oromia | 67.38 (41.60-93.17; $I^2$ = 98.1) |
| | Addis Ababa | 74.51 (69.84-79.17; $I^2$ = 34.5) |
| | Amhara | 73.44 (50.29 – 96. 59; $I^2$ = 97.9) |
| | Tigray | 70.19 (47.10 - 93.28; $I^2$ = 95.7) |
| | SNNPR | Single study |
| Sample size | <223 (median) | 72.41 (60.00 - 83.82; $I^2$ = 96.0) |
| | >=223 (median) | 72.05 (527.44 - 91.66; $I^2$ = 97.5) |
| Study year | Before 2017 | 75.59 (64.28 - 86.9; $I^2$ = 92.1) |

| | |
|---|---|
| After 2017 | 70.50 (56.80 - 84.20; $I^2$ = 92.1) |

SNNPR: South Nations, Nationalities and People Region

## Meta-regression and sensitivity analysis

The sub-group analysis showed that heterogeneity across the studies was widespread. To name the source of heterogeneity, we conducted a meta-regression and sensitivity analysis. During the meta-regression analysis, we conducted using the following study covariance: study years and region. However, the results showed that none of these variables were a statistically significant source of heterogeneity. We also performed a sensitivity analysis to find the influence of each study on the overall effect size. No single study much affected the overall pooled proportion of non-adherence to self-care practice among people with diabetes in Ethiopia (Table 4, Fig. 6).

Table 4: Meta-regression output to explore the heterogeneity of the pooled proportion of clinical outcome of stroke in Ethiopia, 2020.

| Variables | Coefficients | P-value | 95% CI |
|---|---|---|---|
| Study Year | -4.83 | 0.538 | -21.03, 11.35 |
| Region | | | |
| Addis Ababa | -10.68 | 0.660 | -61.37, 40.01 |
| Amhara | -11.67 | 0.626 | -61.63, 38.29 |
| Oromia | -17.72 | 0.468 | -68.44 33.01 |
| Tigray | -14.83 | 0.563 | -68.26 38.61 |

# Discussion

This study was aimed to determine the overall proportion of patient recovery after stroke, and modifiable risk factors in Ethiopia. Accordingly, from all stroke case, more than half (51.40%) of stroke patients in Ethiopia was ischemic stroke. This finding was almost similar to a study done in Kenya (56.1%) (60), much lower than a study done in China (81.9- 91.7%) (61, 62), Burkina Faso (61.63%) (63), Iran (76.5 - 81.9%) (64, 65), and a 22 country case control study (78%) (13). The difference in culture and economic status, lifestyle difference, poor management of modifiable risk factors and difference in the preventive strategies the general public could be the reasons for the difference.

For all stroke case, men stroke patients (54.70%) are relatively higher prevalence than women stroke patient (45.07%) with 54.70: 45.07 split of male and female in all recruited patients in Ethiopia. A systematic review in epidemiological studies on Western European surveys, have shown that stroke is more common in men than in women (66). The gender difference might be men present a higher risk of stroke than women. But women suffer of stroke in older ages making them more

prone to die from stroke than are men. Females were risk avoidance than men such as female less likely to drink heavily or smoke than males (67).

Our meta-analysis showed that almost half (49%) of all stroke patients had hypertension. Previous evidence also showed that 75.8 % of people with stroke had hypertension (63), people with hypertension are two to four times more likely to have stroke (13, 61, 68). This is because hypertension has remained the leading modifiable predictor of stroke morbidity and mortality since 1990 (69). Maintaining a normal blood pressure and lowering high blood pressure can decrease the risk of stroke by 30 to 40% (70). Though hypertension is the main reported modifiable risk factor of stroke among the included primary studies, the pooled proportion of hypertension among people with stroke found in the current study is lower than the previous studies conducted in Burkina Faso (63), Iran (65), China (62), Bosnia-Herzegovina (71), Nigeria (72), and Bangladesh (73). The possible explanation for this variation might be due to the lack of diagnostic modalities and proficiency, level of income, hypertension awareness, treatment, and control (74).

Excessive alcohol consumption is a well-established risk factor of stroke, and our analysis showed that alcohol consumption is the second most common modifiable risk factors of stroke. Almost one-fourth (24.96%) of people with stroke had a history excessive alcohol consumption. This is because drinking excessive amounts of alcohol can trigger atrial fibrillation – a type of irregular heartbeat. Atrial fibrillation increases the risk of stroke by five times, because it can cause blood clots to form in the heart. If these clots move up into the brain, it can lead to stroke (75). A review of 84 studies of alcohol consumption and cardio-vascular disease, alcohol consumption >60 g/day increased the risk of incident stroke by 62% compared with abstaining from alcohol (76). This proportion of alcohol consumption among people with stroke higher than a study conducted in Nigeria (72). The possible explanation for this variation might be due to the lack of

diagnostic modalities and proficiency; measured dyslipidemia in the medical record before the occurrence of stroke. Even though Ethiopia passes tough law regulating alcohol sales and adverts, currently, there is no effective community action to control alcohol consumption (77, 78).

In our current study, dyslipidemia is a third most common modifiable risk factors of stroke. More than two-tenth (20.99%) of people with stroke had dyslipidemia. This is because dyslipidemia promotes cervical or coronary atherosclerosis, which predisposes to athero-thrombotic and cardio-embolic stroke (79). This result is comparable with a previous study conducted in Nigeria (72). However, this estimated proportion dyslipidemia among people with stroke is lower than a study conducted in Bosnia-Herzegovina (71), China (62).

The reasons for the above results could be attributed to the following: first, the dramatic ~~shift of~~ increases in the prevalence of many known risk factors for chronic diseases such as unhealthy lifestyles (e.g. decreased physical activity, excessive smoking, excessive alcohol use, and westernized diet) (80, 81). Second, is the impact of China's rapid economic growth and urbanization (risk of obesity in a subsequently more socio-economically developed environment) (82). In Nigeria, a study postulated that high level of dyslipidemia is closely linked to rapid urbanization and western diet with most urban cities saturated with fast food outlets and increasing sedentary lifestyle which contrasts with our previous highly active agrarian based lifestyle (83).

Diabetes is a well-established risk factor of stroke, and our analysis showed that diabetes mellitus is the fourth most common comorbidity of stroke. More than one-tenth (14.72%) of stroke patients had diabetes mellitus. This is because diabetes causes various micro-vascular and macro-vascular changes often ending in major clinical complications (84). This finding is comparable with the previous study conducted in Nigeria (72); higher than study done in Burkina Faso (63), sub-Saharan African (85) and a previous a systematic review and meta-analysis done in Ethiopia

(86). However, this proportion of diabetes mellitus among people with stroke is lower than a study done in Bosnia-Herzegovina (71) and Iran (65).

Recovery after stroke was the main outcome of interest, and the result found in this study showed that the overall pooled estimated proportion of recovery after stroke in Ethiopia was 72.28% (95%CI:62.48, 82.08). This finding is in line with the ideal proportional recovery rule of stroke (87-89), but still lower than the goal set for 2015 (85%) in the Helsingborg Declaration 2006 (90). Even though recovery after stroke is in line the ideal recovery rule of stroke, this result is still lower than the previous study conducted in Bosnia-Herzegovina (71), Iran (65), sub-Saharan Africa (85), and Kenya (60). The possible reason might be the management protocol for stroke patients was sub-optimal and lagging behind the recommended guidelines due to lack of skilled personnel, appropriate treatment and diagnostic agents in Ethiopia (91). However, this result is relatively higher than previous studies conducted in Ghana (92).

The subgroup analyses by year of studies showed that the overall proportion of recovery after stroke was higher among studies conducted before 2017. The lowest proportion of recovery of stroke in this study population after 20717 may reflect the increased exposure to risk factors for stroke due to ongoing epidemiological and demographic transitions.

# Limitations

There is considerable heterogeneity across the included studies. The observed heterogeneity may be described by differences in the study design, the quality of the studies, and sensitivity. Our study is mainly based on the in-patient cases, and it cannot be externally validated to the general population.

# Implication

This study has many implications for clinical practice and future research. Firstly, the health care provider (especially physicians and nurses) can develop effective strategies to improve recovery after stroke and all modifiable stroke, especially high blood pressure, alcohol consumption behaviors that people with stroke exhibit such modifiable stroke risk factors. Secondly identifying and understanding that restrict to modify these modifiable stroke risk factors is the first step in developing evidence-based interventions to promote short and long-term health outcomes and quality of life. Future research should focus on developing and testing a conceptual model that can ~~use~~ to intervention target accessibility to screening, treatment, sociocultural aspect of stroke risk factor modification in a national context should considered. Finally, to give a long-term reduction in stroke risk factor related co-morbidity, researches should assess ways to extend and sustain modify a stroke risk factors and recovery rate after stroke of this population.

# Conclusion

Even though our pooled result showed that stroke recovery is in line with the ideal proportional recovery rule of stroke, this not satisfactory in the comparison of other developing countries. There is a high burden of stroke in Ethiopia is related with a high rate of modifiable risk factors. More than 90% of patients had one or more modifiable risk factors. All modifiable stroke risk factors, especially high blood pressure and alcohol consumption, should be understood as a major public health problem in Ethiopia and eff orts should be focused on the primary prevention of stroke.

# Declarations

## Abbreviations

DALYs: Disability-Adjusted Life-Years; NCDs: Non-Communicable Diseases; MESH: Medical Subject Headings; SNNPR: South Nations, Nationalities and People Region; NOS: Newcastle-Ottawa Scale

## Ethics approval and consent to participate

Not applicable.

## Consent for publication

Not applicable.

## Acknowledgments

Not applicable

# Reference

1. Gorelick PBJTLN. The global burden of stroke: persistent and disabling. 2019;18(5):417-8.

2. Johnson CO, Nguyen M, Roth GA, Nichols E, Alam T, Abate D, et al. Global, regional, and national burden of stroke, 1990–2016: a systematic analysis for the Global Burden of Disease Study 2016. 2019;18(5):439-58.

3. Yan LL, Li C, Chen J, Miranda JJ, Luo R, Bettger J, et al. Prevention, management, and rehabilitation of stroke in low-and middle-income countries. J Eneurologicalsci. 2016;2:21-30.

4. Mukherjee D, Patil CG. Epidemiology and the global burden of stroke. World neurosurgery. 2011;76(6):S85-S90.

5. Katan M, Luft A, editors. Global burden of stroke. Seminars in neurology; 2018: Georg Thieme Verlag.

6. Kaba MJEJoHD. Non-communicable diseases: unwelcome in Ethiopia. 2018;32(3).

7. Misganaw A, Haregu TN, Deribe K, Tessema GA, Deribew A, Melaku YA, et al. National mortality burden due to communicable, non-communicable, and other diseases in Ethiopia, 1990–2015: findings from the Global Burden of Disease Study 2015. Population health metrics. 2017;15(1):29.

8. BeLue R, Okoror TA, Iwelunmor J, Taylor KD, Degboe AN, Agyemang C, et al. An overview of cardiovascular risk factor burden in sub-Saharan African countries: a socio-cultural perspective. 2009;5(1):10.

9. Feigin VL, Roth GA, Naghavi M, Parmar P, Krishnamurthi R, Chugh S, et al. Global burden of stroke and risk factors in 188 countries, during 1990–2013: a systematic analysis for the Global Burden of Disease Study 2013. 2016;15(9):913-24.

10. Jones P, Jones DJNT. Primary and Secondary Stroke Prevention Strategies. 2017;113(12):42-6.

11. Johnston SC, Mendis S, Mathers CDJTLN. Global variation in stroke burden and mortality: estimates from monitoring, surveillance, and modelling. 2009;8(4):345-54.

12. Mayosi BM, Lawn JE, Van Niekerk A, Bradshaw D, Karim SSA, Coovadia HM, et al. Health in South Africa: changes and challenges since 2009. 2012;380(9858):2029-43.

13. O'donnell MJ, Xavier D, Liu L, Zhang H, Chin SL, Rao-Melacini P, et al. Risk factors for ischaemic and intracerebral haemorrhagic stroke in 22 countries (the INTERSTROKE study): a case-control study. 2010;376(9735):112-23.

14. Gebremariam LW, Chiang C, Yatsuya H, Hilawe EH, Kahsay AB, Godefay H, et al. Non-communicable disease risk factor profile among public employees in a regional city in northern Ethiopia. 2018;8(1):1-11.

15. Shiferaw F, Letebo M, Misganaw A, Feleke Y, Gelibo T, Getachew T, et al. Non-communicable Diseases in Ethiopia: Disease burden, gaps in health care delivery and strategic directions. 2018;32(3).

16. Moher D, Liberati A, Tetzlaff J, Altman DG, med PGJP. Preferred reporting items for systematic reviews and meta-analyses: the PRISMA statement. 2009;6(7):e1000097.

17. Liberati A, Altman DG, Tetzlaff J, Mulrow C, Gøtzsche PC, Ioannidis JP, et al. The PRISMA statement for reporting systematic reviews and meta-analyses of studies that evaluate health care interventions: explanation and elaboration. Journal of clinical epidemiology. 2009;62(10):e1-e34.

18. Modesti PA, Reboldi G, Cappuccio FP, Agyemang C, Remuzzi G, Rapi S, et al. Panethnic differences in blood pressure in Europe: a systematic review and meta-analysis. PloS one. 2016;11(1):e0147601.

19. . Newcastle-Ottawa Scale customized for cross-sectional studies In. available from https://static-content.springer.com/esm/.../12889_2012_5111_MOESM3_ESM.doc.

20. WHO. WHO STEPS stroke manual: the WHO STEPwise approach to stroke surveillance: World Health Organization; 2005.

21. Sacco RL, Kasner SE, Broderick JP, Caplan LR, Connors J, Culebras A, et al. An updated definition of stroke for the 21st century: a statement for healthcare professionals from the American Heart Association/American Stroke Association. 2013;44(7):2064-89.

22. Hoy D, Brooks P, Woolf A, Blyth F, March L, Bain C, et al. Assessing risk of bias in prevalence studies: modification of an existing tool and evidence of interrater agreement. Journal of clinical epidemiology. 2012;65(9):934-9.

23. Higgins JP, Thompson SG, Deeks JJ, Altman DG. Measuring inconsistency in meta-analyses. Bmj. 2003;327(7414):557-60.

24. Cumpston M, Li T, Page MJ, Chandler J, Welch VA, Higgins JP, et al. Updated guidance for trusted systematic reviews: a new edition of the Cochrane Handbook for Systematic Reviews of Interventions. Cochrane Database Syst Rev. 2019;10:ED000142.

25. Borenstein M, Hedges LV, Higgins JP, Rothstein HR. A basic introduction to fixed-effect and random-effects models for meta-analysis. Research synthesis methods. 2010;1(2):97-111.

26. Thompson SG, Higgins JP. How should meta-regression analyses be undertaken and interpreted? Statistics in medicine. 2002;21(11):1559-73.

27. Duval S, Tweedie R. A nonparametric “trim and fill” method of accounting for publication bias in meta-analysis. Journal of the american statistical association. 2000;95(449):89-98.

28. Egger M, Smith GD, Schneider M, Minder C. Bias in meta-analysis detected by a simple, graphical test. Bmj. 1997;315(7109):629-34.

29. Temesgen TG, Teshome B, Njogu PJSr, treatment. Treatment outcomes and associated factors among hospitalized stroke patients at Shashemene Referral Hospital, Ethiopia. 2018;2018.

30. Beyene D, Asefa H. A two year retrospective cross-sectional study on prevalence, associated factors and treatment outcome among patients admitted to medical ward (stroke unit) at Jimma University Medical Center, Jimma, South West, Ethiopia, 2018. J Palliat Med Care. 2018;5(4):1-6.

31. Kefale B, Betero G, Temesgen G, Degu A. Management practice, and treatment outcome and its associated factors among hospitalized stroke patient at Ambo University Referral Hospital, Ethiopia: an Institutional Based Cross Sectional Study. 2019.

32. Fekadu G, Chelkeba L, Kebede AJBn. Risk factors, clinical presentations and predictors of stroke among adult patients admitted to stroke unit of Jimma university medical center, south west Ethiopia: prospective observational study. 2019;19(1):1-11.

33. Bedassa TSJIR. Assessment of the types and factors associated with stroke among adult patients admitted in Adama Hospital Medical College, Ethiopia. 2019;6:S490.

34. Dandena A, Sinaga M, Yirga Y, Zelalem T. CT Scan Pattern of Stroke Patients at Jimma University Medical Center, South West Ethiopia. J Biomedical Journal of Scientific Technical Research. 2020;29(4):22652-7.

35. Fekadu G, Chelkeba L, Kebede AJBn. Burden, clinical outcomes and predictors of time to in hospital mortality among adult patients admitted to stroke unit of Jimma university medical center: a prospective cohort study. 2019;19(1):213.

36. Fekadu G, Adola B, Mosisa G, Shibiru T, Chelkeba L. Clinical characteristics and treatment outcomes among stroke patients hospitalized to Nekemte referral hospital, western Ethiopia. Journal of Clinical Neuroscience. 2020;71:170-6.

37. Sultan M, Debebe F, Azazh A, Hassen GWJEJoHD. Epidemiology of stroke patients in Tikur Anbessa Specialized Hospital: Emphasizing clinical characteristics of hemorrhagic stroke patients. Ethiop J Health Dev. 2017;31(1):13-7.

38. Gedefa B MT, Berhe T, Abera H. Assessment of risk factors and treatment outcome of stroke admissions at St. Paul's teaching hospital, addis ababa, Ethiopia. Neurophysiology. 2017;8:1-6.

39. Zewdie A DF, Kebede S, Azazh A, Laytin A, Pashmforoosh G. et al. Prospective assessment of patients with stroke in Tikur Anbessa specialised hospital, Addis Ababa, Ethiopia. African Journal of Emergency Medicine. 2018;8(1):21-4.

40. Asres AK CA, Bedada T, Gebrekidan H. Frequency, nursing managements and stroke patients' outcomes among patients admitted to Tikur Anbessa specialized hospital, Addis Ababa, Ethiopia a retrospective, institution based cross-sectional study. International Journal of Africa Nursing Sciences. 2020: Jan 1;13:100228.

41. Ayehu Kassaw AC, Tadesse Bedada. Prevalence, nursing managements and patients' outcomes among stroke patients admitted to Tikur Anbessa specialized hospital, Addis Ababa, Ethiopia, 2018: Addis Ababa University; 2018.

42. Zenebe G, Alemayehu M, Asmera JJEmj. Characteristics and outcomes of stroke at Tikur Anbessa Teaching Hospital, Ethiopia. 2005;43(4):251-9.

43. Gelan Y, Weldeab A. Predictors of Stroke Mortality among Patients Admitted to a Hospital in Ethiopia (2070). AAN Enterprises; 2020.

44. Erkabu SG AY, Mihretu DD, Semere A, Alemu YM. Ischemic and hemorrhagic stroke in Bahir Dar, Ethiopia: a retrospective hospital-based study. Journal of Stroke and Cerebrovascular Diseases. 2018;27(6):1533-8.

45. Gebreyohannes EA BA, Abebe TB, Seid MA, Haile KT. In-hospital mortality among ischemic stroke patients in Gondar University Hospital: a retrospective cohort study. Stroke research and treatment. 2019;2019.

46. Greffie ES MT, Getahun S. Risk factors, clinical pattern and outcome of stroke in a referral hospital, Northwest Ethiopia. Clin Med Res. 2015;4(6):182-8.

47. Baye M HA, Gordon-Murer C, Mariscal T, Belay GJ, Gebremariam AA et al. Stroke Characteristics and Outcomes of Adult Patients in Northwest Ethiopia. Frontiers in Neurology. 2020;11:428.

48. Mulugeta H, Yehuala A, Haile D, Mekonnen N, Dessie G, Kassa GM, et al. Magnitude, risk factors and outcomes of stroke at Debre Markos Referral Hospital, Northwest Ethiopia: a retrospective observational study. The Egyptian Journal of Neurology, Psychiatry and Neurosurgery. 2020;56:1-9.

49. Mulat B MJ, Yeseni M, Alamirew M, Dermello M, Asemahagn MA. Magnitude of stroke and associated factors among patients who attended the medical ward of Felege Hiwot Referral Hospital, Bahir Dar town, Northwest Ethiopia  Ethiopian Journal of Health Development. 2016;30(3):129-34.

50. Tamirat KS AS, Gebi NB, Gerffie ES. Clinical profile, in-hospital outcome and associated factors of stroke after the start of a standard organized stroke care unit at university of Gondar hospital, northwest Ethiopia. 2019. p. 15.

51. Mekonen HH, Birhanu MM, Mossie TB, Gebreslassie HT. Factors associated with stroke among adult patients with hypertension in Ayder Comprehensive Specialized Hospital, Tigray, Ethiopia, 2018: A case-control study. J PloS one. 2020;15(2):e0228650.

52. Gufue ZH GN, Ayele W, Yifru YM, Hailu NA, Welesemayat ET et al Survival of Stroke Patients According to Hypertension Status in Northern Ethiopia: Seven Years Retrospective Cohort Study. Vascular Health and Risk Management 2020;16:389.

53. Gebremariam SA, Yang HS. Types, risk profiles, and outcomes of stroke patients in a tertiary teaching hospital in northern Ethiopia. J Eneurologicalsci. 2016;3:41-7.

54. Asgedom SW, Gidey K, Gidey K, Niriayo YL, Desta DM, Atey TM. Medical complications and mortality of hospitalized stroke patients. J Journal of Stroke Cerebrovascular Diseases. 2020;29(8):104990.

55. Deresse B, Shaweno DJJotns. Epidemiology and in-hospital outcome of stroke in South Ethiopia. 2015;355(1-2):138-42.

56. Gebreyohannes EA, Bhagavathula AS, Abebe TB, Seid MA, Haile KTJSr, treatment. In-hospital mortality among ischemic stroke patients in Gondar University Hospital: a retrospective cohort study. 2019;2019.

57. Gedefa B, Menna T, Berhe T, Abera H. Assessment of risk factors and treatment outcome of stroke admissions at St. Paul's teaching hospital, addis ababa, Ethiopia. Journal of Neurology Neurophysiology. 2017;8(3):1-6.

58. Greffie ES, Mitiku T, Getahun S. Risk factors, clinical pattern and outcome of stroke in a referral hospital, Northwest Ethiopia. J Clin Med Res. 2015;4(6):182-8.

59. Tamirat K, Abdella S, Gebi N, Gerffie E. Clinical profile, in-hospital outcome and associated factors of stroke after the start of a standard organized stroke care unit at university of Gondar hospital, northwest Ethiopia. 2019.

60. Kaduka L, Muniu E, Oduor C, Mbui J, Gakunga R, Kwasa J, et al. Stroke mortality in Kenya's public tertiary hospitals: a prospective facility-based study. 2018;8(2):70-9.

61. Yi X, Luo H, Zhou J, Yu M, Chen X, Tan L, et al. Prevalence of stroke and stroke related risk factors: a population based cross sectional survey in southwestern China. 2020;20(1):5.

62. Zhang F-L, Guo Z-N, Wu Y-H, Liu H-Y, Luo Y, Sun M-S, et al. Prevalence of stroke and associated risk factors: a population based cross sectional study from northeast China. 2017;7(9):e015758.

63. Samadoulougou DRS, Kpoda H, Traore I, Savadogo L, Sombie I, Millogo A, editors. Evolution of the magnitude of stroke at the teaching hospital of Bobo-Dioulasso. World Congress on Clinical Trials in Diabetes; 2016: BioScientifica.

64. Ghandehari KJGMJ. Epidemiology of stroke in Iran. 2016;5(S1):3-9.

65. Farhoudi M, Mehrvar K, Sadeghi-Bazargani H, Hashemilar M, Seyedi-Vafaee M, Sadeghi-Hokmabad E, et al. Stroke subtypes, risk factors and mortality rate in northwest of Iran. 2017;16(3):112.

66. Appelros P, Stegmayr B, Terént AJS. Sex differences in stroke epidemiology: a systematic review. 2009;40(4):1082-90.

67. Yao X-y, Lin Y, Geng J-l, Sun Y-m, Chen Y, Shi G-w, et al. Age-and gender-specific prevalence of risk factors in patients with first-ever ischemic stroke in China. 2012;2012.

68. Chobanian AV, Bakris GL, Black HR, Cushman WC, Green LA, Izzo Jr JL, et al. Seventh report of the joint national committee on prevention, detection, evaluation, and treatment of high blood pressure. hypertension. 2003;42(6):1206-52.

69. Avan A, Digaleh H, Di Napoli M, Stranges S, Behrouz R, Shojaeianbabaei G, et al. Socioeconomic status and stroke incidence, prevalence, mortality, and worldwide burden: an ecological analysis from the Global Burden of Disease Study 2017. 2019;17(1):191.

70. Lawes CM, Bennett DA, Feigin VL, Rodgers AJS. Blood pressure and stroke: an overview of published reviews. 2004;35(3):776-85.

71. Bender M, Jusufovic E, Railic V, Kelava S, Tinjak S, Dzevdetbegovic D, et al. High burden of stroke risk factors in developing country: the case study of Bosnia-Herzegovina. 2017;29(4):277.

72. Watila M, Ibrahim A, Balarabe S, Gezawa I, Bakki B, Tahir A, et al. Risk factor profile among black stroke patients in Northeastern Nigeria. 2012;4(5):50-8.

73. Hossain A, Ahmed N, Rahman M, Islam M, Sadhya G, Fatema KJFMCJ. Analysis of sociodemographic and clinical factors associated with hospitalized stroke patients of Bangladesh. 2011;6(1):19-23.

74. Mills KT, Bundy JD, Kelly TN, Reed JE, Kearney PM, Reynolds K, et al. Global disparities of hypertension prevalence and control: a systematic analysis of population-based studies from 90 countries. 2016;134(6):441-50.

75. Alcohol and stroke, Factsheet13 (2014).

76. Paul E Ronksley SEB, Barbara J Turner, Kenneth J Mukamal, William A Ghali. Association of alcohol consumption with selected cardiovascular disease outcomes: a systematic review and meta-analysis. BMJ. 2011:342:d671.

77. Beyene NJJophp. Alcohol control policy in Ethiopia and implications for public health. 2019;40(4):423-35.

78. Getachew T, Defar A, Teklie H, Gonfa G, Bekele A, Bekele A, et al. Magnitude and predictors of excessive alcohol use in Ethiopia. 2017;31(1).

79. Ayata C, Shin HK, Dileköz E, Atochin DN, Kashiwagi S, Eikermann-Haerter K, et al. Hyperlipidemia disrupts cerebrovascular reflexes and worsens ischemic perfusion defect. 2013;33(6):954-62.

80. Popkin BMJHA. Will China's nutrition transition overwhelm its health care system and slow economic growth? 2008;27(4):1064-76.

81. Song P, Zha M, Yang X, Xu Y, Wang H, Fang Z, et al. Socioeconomic and geographic variations in the prevalence, awareness, treatment and control of dyslipidemia in middle-aged and older Chinese. 2019;282:57-66.

82. Xu S, Ming J, Yang C, Gao B, Wan Y, Xing Y, et al. Urban, semi-urban and rural difference in the prevalence of metabolic syndrome in Shaanxi province, northwestern China: a population-based survey. 2014;14(1):104.

83. Oguejiofor O, Onwukwe C, Odenigbo CJAoAm. Dyslipidemia in Nigeria: prevalence and pattern. 2012;11(4):197.

84. Chen R, Ovbiagele B, Feng WJTAjotms. Diabetes and stroke: epidemiology, pathophysiology, pharmaceuticals and outcomes. 2016;351(4):380-6.

85. Lekoubou A, Clovis N, Dzudie A, Kengne APJPCD. Diagnosed diabetes mellitus and in-hospital stroke mortality in a major sub-Saharan African urban medical unit. 2017;11(1):57-62.

86. Alene M, Assemie MA, Yismaw L, Ketema DBJBn. Magnitude of risk factors and in-hospital mortality of stroke in Ethiopia: a systematic review and meta-analysis. 2020;20(1):1-10.

87. Veerbeek JM, Winters C, van Wegen EE, Kwakkel GJPo. Is the proportional recovery rule applicable to the lower limb after a first-ever ischemic stroke? 2018;13(1):e0189279.

88. Hawe RL, Scott SH, Dukelow SPJS. Taking proportional out of stroke recovery. 2019;50(1):204-11.

89. Krakauer JW, Marshall RSJAon. The proportional recovery rule for stroke revisited. 2015;78(6):845-7.

90. Kjellstrom T NB, Shatchkute A. Helsingborg Declaration 2006 on European stroke strategies. Cerebrovasc Disease. 2007;23:231-41.

91. Ginenus Fekadu LC, Tsegaye Melaku, Busha Gamachu, Mohammed Gebre, Firomsa Bekele, Getahun Fetensad. Management protocols and encountered complications among stroke patients admitted to stroke unit of Jimma university medical center, Southwest Ethiopia: Prospective observational study. Annals of Medicine Surgery. 2019:135–43.

92. Sarfo FS, Akassi J, Awuah D, Adamu S, Nkyi C, Owolabi M, et al. Trends in stroke admission and mortality rates from 1983 to 2013 in central Ghana. 2015;357(1-2):240-5.

## Figure Legends

Figure 1: Flow chart to a selection of studies for a systematic review and meta-analysis of the proportion of adherence to healthy lifestyle modification of people with hypertension in Ethiopia 2020.

Figure 2: Frost plot of in the proportion of hemorrhagic stroke in Ethiopia, 2020.

Figure 3 Forest plot of in the proportion of ischemic stroke in Ethiopia, 2020.

Figure 4: Forest plot of the proportion of recover during discharge after stroke in Ethiopia, 2020.

Figure 5: Meta funnels presentations of the proportion of recover after stroke in Ethiopia, 2020, whereby SE PIV (standard error of proportion) plotted on the Y-axis and log PIV (logarithm of proportion.

Figure 6: One-leave-out sensitivity analysis for studies conducted on the pooled estimated proportion of stroke clinical outcome in Ethiopia, 2020.

Click here to access/download
**Supporting Information**
s1 search technique.docx

Click here to access/download
**Supporting Information**
001Stroke figure plos.docx

Click here to access/download
**Supporting Information**
01 Appendix 3 quality assessment NOS.xlsx

Click here to access/download
**Supporting Information**
01S2 qaulity appraisal risk assessment.docx

Click here to access/download

**Supporting Information**

S3 sex proportion of stroke in Ethiopia.docx

Click here to access/download
**Supporting Information**
S4 PRISMA check list.docx
