# Supplementary material for: The burden of stroke and modifiable risk factors in Ethiopia: A systemic review and meta-analysis
Source: PLoS One. 2021 Nov 1;16(11):e0259244. doi: 10.1371/journal.pone.0259244 (PMC8559958; doi:10.1371/journal.pone.0259244)
Supplement: Attachment — Submitted filename: plosone review_IS.pdf [file pone.0259244.s009.pdf]

PLOS ONE

The burden of stroke and modifiable risk factors in Ethiopia: a systemic review and meta-analysis.

--Manuscript Draft--

**Manuscript Number:**	PONE-D-21-01404R1
**Article Type:**	Research Article
**Full Title:**	The burden of stroke and modifiable risk factors in Ethiopia: a systemic review and meta-analysis.
**Short Title:**	The burden of stroke and modifiable risk factors in Ethiopia
**Corresponding Author:**	Teshager Weldegiorgis Abate<br>Bahir Dar University<br>Bahir Dar, ETHIOPIA
**Keywords:**	stroke; modifiable risk factors; Stroke recovery rate; Meta-analysis; Ethiopia
**Abstract:**	Background: The burden and contribution of modifiable risk factors of stroke in Ethiopia are unclear. Knowledge about this burden and modifying risk factors is pivotal for establishing stroke prevention strategies. In recent decades, the issue of modifiable risk factors a key to improves the quality of life. Modify the risk factor is an important issue to prevent stroke. So far, many primary studies conducted to estimate the proportion of stroke burden and modifiable risk factors in Ethiopia. However, the lack of a nationwide study that determines the overall pooled estimation burden and modifiable risk factors of stroke is a research gap.<br>Methods: To conduct this review, we are following the PRISMA checklist. Three reviewers have been searched and extracted from the CINAHL (EBSCO), MEDLINE (via Ovid), PubMed, EMcare, African Journals Online (AJOL), and Google scholar. The quality of the primary study ~~assess~~ using the Newcastle-Ottawa Scale (NOS) by two independent reviewers, and only studies with low and moderate risk include in the final analysis. The review presented the pooled proportion of stroke recovery and modifiable risk factors of stroke in stroke patients. The registered protocol number in PROSPERO is CRD42020221906.<br>Results: In this study, the pooled proportion stroke recovery in Ethiopia was 72.28% (95%CI: 62.48, 82.08; I2 = 96.5%). The overall magnitude modifiable risk factor of hypertension, alcohol consumption and dyslipidemia among stroke patients were 49% (95%CI: 43.59, 54.41), 24.96% (95CI%:15.01, 34.90), and 20.99% (95%CI: 11.10, 30.88), respectively. The least proportion of stroke recovery was in the Oromia region (67.38 (95%CI: 41.60-93.17; I2 = 98.1%). Besides, the proportion of stroke recovery was decreased after 2017 (70.50 (56.80 - 84.20).<br>Conclusions: In our study, more than 90% of stroke patients had one or more modifiable risk factors. All identified modifiable stroke risk factors are major public health issues in Ethiopia. Therefore, efforts focused on the primary prevention of stroke. Such as lower blood pressure, lose weight, exercise more, don't drink alcohol, or do it in moderation, treat atrial fibrillation and diabetes, and quit smoking.
**Order of Authors:**	Teshager Wldegiorgis Abate
	Balew Zeleke
	Ashenafi Genanew
	Bidiru Weldegiorgis Abate
**Response to Reviewers:**	Response to Reviewers<br>Response to editor and reviewers'<br>Response to the editor:<br>We thank you and the reviewers for a thorough reading and constructive criticism of our manuscript and for the opportunity to revise and resubmit. We are pleased to submit the improved research article, including a proposed comment, "The burden of stroke and modifiable risk factors in Ethiopia: a systemic review and meta-analysis with a manuscript ID of PONE-D-21-01404"<br>1.General Comments:<br>#1. COMMENT: Please ensure that your manuscript meets PLOS ONE's style

requirements, including those for file naming.
RESPONSE: We have checked and attest that all formatting and style requirements have
been met PLOS ONE's style requirements.
#2. COMMENT: The funders had no role in study design, data collection and analysis, decision to publish, or preparation of the manuscript. At this time, please address the following queries…
RESPONSE: The authors received no specific funding for this work.
#3. COMMENT: Please include captions for your Supporting Information files at the end of your manuscript, and update any in-text citations to match accordingly.
RESPONSE: We include all capitation
#4. COMMENT: Thank you for submitting the above manuscript to PLOS ONE. During our internal evaluation of the manuscript, we found significant text overlap between your submission and the following previously published works, some of which you are an author.
RESPONSE: We revise the manuscript to rephrase the duplicated text.
2.Review Comments to the Author
REVIEWER #1 COMMENTS
1.COMMENT: There are multiple language errors, grammatical mistakes, typing errors which alter the meaning of what the authors may be trying to convey.
RESPONSE: the language expert copyedited the manuscript for language, spelling, grammar and sentence structure.
2.COMMENT: There is use of different fonts in different sections of the manuscript, which the authors need to address.
RESPONSE: Thank you this comment. We make uniform of fonts throughout the manuscript.
3.COMMENT: The discussion section needs to highlight the reasons for the difference in results of each significant risk factor when compared to other studies quoted.
RESPONSE: We try to highlight the reason from the different results of each significant risk factor when compared to other studies quoted.
4.COMMENT: The conclusion should emphasize how this study adds to literature; authors should point out feasible strategies suggested by them that can help address the modifiable risk factors in Ethiopia.
RESPONSE: We accept the comment and we emphasize feasible strategies in the nation- Ethiopia.
REVIEWER #2 COMMENTS
1.COMMENT: Revise the research type as "review" not "research article"
RESPONSE: we accept the comment and replace research article" by "review"
2. COMMENT: Explain the operational definition you have used regarding "alcohol consumption", its broad term, is it any alcohol consumption?
RESPONSE: when to say alcohol consumption, more than two drinks in a day for men and more than 1 drink in a day for women
3.COMMENT: You have not used "AJOL: database. However, most of articles from Ethiopia were published on local and regional journals; which are indexed by AJOL. So, I believe by not including AJOL in your searching process, you may miss many articles from Ethiopia.
RESPONSE: we included AJOL for a data bases
4.COMMENT: You have also included un-published articles. But how did you access this un-published articles? Because I know there are many published and un-published articles from our departments you haven't included.
RESPONSE: we access the institutional repository (like Jimma University, Addis Ababa University). If you have an included article, please share a link and we are ready to included

5.COMMENT: The search key words are very few, to capture all modifiable stroke risk factor. Please explain this issue.
RESPONSE: we select the most public health important modifiable factors
6.COMMENT: In the Analysis process, since the authors mentioned meta-analysis, they should present their results in "Forest plot", which they didn't included.
RESPONSE: we try to present the result in 'Forest plot' in figure 2 to figure 4.
7.COMMENT: Please revise your discussion section by comparing your findings with other regions mainly focusing on points unique to this region.
RESPONSE: we try to discuss by comparing our findings with other regions mainly focusing on points unique to this region.

	8.COMMENT: Include your data extraction detail tables as a supplementary file for detail evaluation of your work.<br>RESPONSE: we include data extraction detail tables as a supplementary file.<br>REVIEWER #3 COMMENTS<br>1.COMMENT: The Title: was unclear and ambiguous as one doesn't understand. So this title should be changed to (for example): Burden of stroke in Ethiopia.<br>RESPONSE: we changed the tittle as recommended "Burden of stroke and modifiable risk factors in Ethiopia: a systemic review and meta-analysis."<br>On abstract part<br>2.COMMENT: abstract on back ground, line 2, 3. This sentence is not correct as the contribution of modifiable risk factors to the global burden of stroke is well studied in the international literature, so they should remove the word global from the sentence and specify the sentence to be regional (in Ethiopia).<br>RESPONSE: the sentence is specified in regional context-Ethiopia.<br>3.COMMENT: Back ground line 3: crucial for informing stroke prevention strategies. This sentence is wrong, they should write for establishing stroke prevention strategies and I suggest that, the language of the paper should be revised by native speaker to be more clear and informative.<br>RESPONSE: we accept the comment and write as the reviewer suggestion.<br>4.COMMENT: back ground line 5: one of the keys to improving the quality of life. To improve not to improving, and as I said before the language of paper should be revised and rewritten by native speaker<br>RESPONSE: we accept the comment and write as the reviewer suggestion.<br>5.COMMENT: Conclusion: needs to be rewritten, it should be more concise and informative.<br>RESPONSE: we try to revised the conclusion section.
**Additional Information:**	
**Question**	**Response**
**Financial Disclosure**<br><br>Enter a financial disclosure statement that describes the sources of funding for the work included in this submission. Review the submission guidelines for detailed requirements. View published research articles from *PLOS ONE* for specific examples.<br><br>This statement is required for submission and **will appear in the published article** if the submission is accepted. Please make sure it is accurate.	The authors received no specific funding for this work.

Powered by Editorial Manager® and ProduXion Manager® from Aries Systems Corporation

**Unfunded studies**<br>Enter: *The author(s) received no specific funding for this work.*<br><br>**Funded studies**<br>Enter a statement with the following details:<br>• Initials of the authors who received each award<br>• Grant numbers awarded to each author<br>• The full name of each funder<br>• URL of each funder website<br>• Did the sponsors or funders play any role in the study design, data collection and analysis, decision to publish, or preparation of the manuscript?<br>• **NO** - Include this sentence at the end of your statement: *The funders had no role in study design, data collection and analysis, decision to publish, or preparation of the manuscript.*<br>• **YES** - Specify the role(s) played.<br><br>* typeset	
**Competing Interests**<br><br>Use the instructions below to enter a competing interest statement for this submission. On behalf of all authors, disclose any competing interests that could be perceived to bias this work—acknowledging all financial support and any other relevant financial or non-financial competing interests.<br><br>This statement is required for submission and **will appear in the published article** if the submission is accepted. Please make sure it is accurate and that any funding sources listed in your Funding Information later in the submission form are also declared in your Financial Disclosure statement.<br><br>View published research articles from *PLOS ONE* for specific examples.	NO authors have competing interests

**NO authors have competing interests**<br><br>Enter: *The authors have declared that no competing interests exist.*<br><br>**Authors with competing interests**<br><br>Enter competing interest details beginning with this statement:<br><br>*I have read the journal's policy and the authors of this manuscript have the following competing interests: [insert competing interests here]*<br><br>* typeset	
**Ethics Statement**<br><br>Enter an ethics statement for this submission. This statement is required if the study involved:<br><br>• Human participants<br>• Human specimens or tissue<br>• Vertebrate animals or cephalopods<br>• Vertebrate embryos or tissues<br>• Field research<br><br>Write "N/A" if the submission does not require an ethics statement.<br><br>General guidance is provided below. Consult the submission guidelines for detailed instructions. **Make sure that all information entered here is included in the Methods section of the manuscript.**	N/A

Powered by Editorial Manager® and ProduXion Manager® from Aries Systems Corporation

**Format for specific study types**<br><br>**Human Subject Research (involving human participants and/or tissue)**<br>• Give the name of the institutional review board or ethics committee that approved the study<br>• Include the approval number and/or a statement indicating approval of this research<br>• Indicate the form of consent obtained (written/oral) or the reason that consent was not obtained (e.g. the data were analyzed anonymously)<br><br>**Animal Research (involving vertebrate animals, embryos or tissues)**<br>• Provide the name of the Institutional Animal Care and Use Committee (IACUC) or other relevant ethics board that reviewed the study protocol, and indicate whether they approved this research or granted a formal waiver of ethical approval<br>• Include an approval number if one was obtained<br>• If the study involved *non-human primates*, add *additional details* about animal welfare and steps taken to ameliorate suffering<br>• If anesthesia, euthanasia, or any kind of animal sacrifice is part of the study, include briefly which substances and/or methods were applied<br><br>**Field Research**<br><br>Include the following details if this study involves the collection of plant, animal, or other materials from a natural setting:<br>• Field permit number<br>• Name of the institution or relevant body that granted permission	
**Data Availability**<br><br>Authors are required to make all data underlying the findings described fully available, without restriction, and from the time of publication. PLOS allows rare exceptions to address legal and ethical concerns. See the PLOS Data Policy and FAQ for detailed information.	Yes - all data are fully available without restriction

A Data Availability Statement describing where the data can be found is required at submission. Your answers to this question constitute the Data Availability Statement and **will be published in the article**, if accepted.<br><br>**Important:** Stating 'data available on request from the author' is not sufficient. If your data are only available upon request, select 'No' for the first question and explain your exceptional situation in the text box.<br><br>Do the authors confirm that all data underlying the findings described in their manuscript are fully available without restriction?	
**Describe where the data may be found in full sentences. If you are copying our sample text, replace any instances of XXX with the appropriate details.**<br><br>• If the data are **held or will be held in a public repository**, include URLs, accession numbers or DOIs. If this information will only be available after acceptance, indicate this by ticking the box below. For example: *All XXX files are available from the XXX database (accession number(s) XXX, XXX.).*<br>• If the data are all contained **within the manuscript and/or Supporting Information files**, enter the following: *All relevant data are within the manuscript and its Supporting Information files.*<br>• If neither of these applies but you are able to provide **details of access elsewhere**, with or without limitations, please do so. For example:<br><br>*Data cannot be shared publicly because of [XXX]. Data are available from the XXX Institutional Data Access / Ethics Committee (contact via XXX) for researchers who meet the criteria for access to confidential data.*<br><br>*The data underlying the results presented in the study are available from (include the name of the third party*	All relevant data are within the manuscript and its Supporting Information files.

and contact information or URL).<br>• This text is appropriate if the data are owned by a third party and authors do not have permission to share the data.<br><br>* typeset	
Additional data availability information:	

Powered by Editorial Manager® and ProduXion Manager® from Aries Systems Corporation

Manuscript Click here to access/download;Manuscript;Manuscript.docx

The burden of stroke and modifiable risk factors in Ethiopia: a systemic review and meta-analysis.

Teshager Weldegiorgis Abate[1]*, Balew Zeleke[2&], Ashenafi Genanew[3¶], and Bidiru Weldegiorgis Abate[4¶]

[1]Department of adult health Nursing, School of Health Science, College of Medicine and Health Science, Bahir Dar University, P.O. Box 79, Bahir Dar, Ethiopia.

[2]Department of Pediatric and Child Health Nursing, School of Health Sciences, College of Medicine and Health Sciences, Bahir Dar University, P.O. Box 79, Bahir Dar, Ethiopia.

[3]Department of pharmacology, School of Health Sciences, College of Medicine and Health Science, Bahir Dar University, P.O. Box 79, Bahir Dar, Ethiopia.

[4]Department of Anesthetics, Addis Alem Hospitla, Amhara Regional Bureau

*Corresponding author: teshagerhylemarriam@gmail.com, phone number +251912886162

[¶]These authors contributed equally to this work.

[&]This authors also contributed equally to this work

Abstract

Background: The burden and contribution of modifiable risk factors of stroke in Ethiopia are unclear. Knowledge about this burden and modifying risk factors is pivotal for establishing stroke prevention strategies. In recent decades, the issue of modifiable risk factors a key to improves the quality of life. Modify the risk factor is an important issue to prevent stroke. So far, many primary studies conducted to estimate the proportion of stroke burden and modifiable risk factors in Ethiopia. However, the lack of a nationwide study that determines the overall pooled estimation burden and modifiable risk factors of stroke is a research gap**.**

Methods: To conduct this review, we are following the PRISMA checklist. Three reviewers have been searched and extracted from the CINAHL (EBSCO), MEDLINE (via Ovid), PubMed, EMcare, African Journals Online (AJOL), and Google scholar. The quality of the primary study assess using the Newcastle-Ottawa Scale (NOS) by two independent reviewers, and only studies with low and moderate risk include in the final analysis. The review presented the pooled proportion of stroke recovery and modifiable risk factors of stroke in stroke patients. The registered protocol number in PROSPERO is CRD42020221906.

Results: In this study, the pooled proportion stroke recovery in Ethiopia was 72.28% (95%CI: 62.48, 82.08; I2 = 96.5%). The overall magnitude modifiable risk factor of hypertension, alcohol consumption and dyslipidemia among stroke patients were 49% (95%CI: 43.59, 54.41), 24.96% (95CI%:15.01, 34.90), and 20.99% (95%CI: 11.10, 30.88), respectively. The least proportion of stroke recovery was in the Oromia region (67.38 (95%CI: 41.60-93.17; I2 = 98.1%). Besides, the proportion of stroke recovery was decreased after 2017 (70.50 (56.80 - 84.20).

Conclusions: In our study, more than 90% of stroke patients had one or more modifiable risk factors. All identified modifiable stroke risk factors are major public health issues in Ethiopia.

Therefore, efforts focused on the primary prevention of stroke. Such as lower blood pressure, lose weight, exercise more, don't drink alcohol, or do it in moderation, treat atrial fibrillation and diabetes, and quit smoking.

Keywords: Stroke, Modifiable risk factors, Stroke recovery rate, Meta-analysis, Ethiopia

Background

Stroke remains the second leading cause of death worldwide ~~in all sexes~~, with an annual mortality rate of 5·5 million. Fewer women (2·6 million) than men (2·9 million) died from stroke (1, 2). The incidence, prevalence, and mortality rate of stroke have ~~been~~ increased worldwide, with most of the burden being in the low and middle-income ~~country~~ including Ethiopia (3, 4). Hemorrhagic stroke is responsible for more deaths and Disability-Adjusted Life-Years (DALYs). Incidence and mortality of stroke differ between countries, geographical regions, and ethnic groups (5).

Ethiopia faces the unenviable threat of a triple burden of disease: infectious diseases, Non-Communicable Diseases (NCDs), and injuries ~~are steadily increasing and pose risks to the lives of Ethiopians~~ (6). Although Ethiopia is progressing towards national health coverage, the country faces the double burden of both communicable and NCD (7). The magnitude of stroke-related deaths in Ethiopia is 6.23% out of total deaths, and the age-adjusted death rate of stroke in the country is 89.82 per 100 000 of the population (8). Besides, previous reports indicated that 90% of the burdens of stroke ~~are~~ attributable to modifiable risk factors (9). Of this, three-quarters of the stroke burden is attributable to behavioral risk factors (10).

Most of the stroke burden was attributable to modifiable risks. ~~These are:~~ metabolic factors (high blood pressure, obesity, fasting plasma glucose, cardiac disorder, and total cholesterol)

accounted for 72% of stroke DALYs, and behavioral factors (smoking, poor diet, and physical inactivity) accounted for 66%. (5, 11-15).

In Ethiopia, a comprehensive national representative stroke burden and the modifiable risk factor are lacking. Thus, this study aimed to determine the overall magnitude of stroke, modifiable risk factors of stroke, and improvement rate after stroke in Ethiopia.

Methods and analysis

Protocol design and registration

A systematic review with a meta-analysis of published and unpublished observational studies is incorporated to assess the magnitude of stroke and modifiable risk factors of stroke in Ethiopia. The Preferred Reporting Items for Systematic Review and Meta-analysis Protocol (PRISMA-P) (16, 17), and Meta-analysis of Observational Studies in Epidemiology (MOOSE) guideline statement (18) used to the development of this study protocol, the conduct and design, and the reporting of these results. This review protocol register in the International Registration of Systems Reviews (PROSPERO) (registration number CRD42020221906).

Eligibility Criteria

The eligibility of the study was determined using the following criteria: (1) all facility-based observational studies; (2) all studies conducted in Ethiopia; (3) all studies reporting either the magnitude of any subtypes of stroke or rate of improvement at discharge and modifiable risk factors; and both published and unpublished studies. On the other hand, anonymous reports, case reports, qualitative, and unable to access the full text after three email contacts of principal investigators of the particular studies exclude the analysis.

Information source and search strategies

We used standardized and well-described methods in this systemic review (16). Briefly, a search strategy design using the relevant Medical Subject Headings (MESH), keywords, and synonyms. The search strategy for PubMed: the keywords which we used in our search included terms describing stroke, age, and modifiable risk factors as shown in the search strategy as follows: (1) (Stroke [Title] OR "Ischemic stroke"[Title] OR "Ischaemic stroke"[Title] OR "Haemorrhagic stroke"[Title] OR "Hemorrhagic stroke" OR "Cerebral Vascular Accident" OR CVA); (2) (Adults OR "18 years or older") [Text Word] (3) (Ethiopia) [Text Word] **(**4**)** (Hypertension OR "High blood pressure" [Text Word] OR Diabetes [Text Word] OR "Diabetes mellitus" OR "Smoking" OR "Obesity" OR Alcohol OR "Heavy drinking" [Text Word] OR Physical exercise OR "Physical activity" [Text Word] OR (High blood cholesterol level OR "Hypercholesterolemia, Hyperlipidemia" OR "Hyperlipoproteinemia" OR "Arterial fibrillation) [Text Word] (5) #1 AND #2 AND #3 AND #4 (S1.).

A pretest of the search strategy by two authors perform in PubMed. The actual electronic search did from November 20 to 25, 2020. Two independent reviewers implemented the electronic search in the following electronic databases: CINAHL (EBSCO), MEDLINE (via Ovid), PubMed, EMcare, AJOL, and Google scholar search engines. Finally, the search process present in a PRISMA flow chart.

Study Selection

Two of the reviewers (TWA and BWA) screened the titles and abstracts of each article to find potentially eligible studies. After removing duplicates, the search results ~~are~~ exported to End-Note software (version X7 Thomson Reuters, New York, NY) to create a bibliographical database of the retrieved references. The selection process ~~conduct~~ in two stages: first screening of titles and abstracts against the predetermined inclusion/exclusion criteria, followed by a second

screening of the full text of the research reports identified as probably relevant in the initial screening. Both stages were carried out independently by two authors (TWA and AG), and disagreements resolved by discussion with another author (BWA) determined the eligibility of the included in the review.

Data Extraction process and Quality Assessment

The abstract and full-text review data abstraction was done by three independent authors (TWA, BZ, and AG) using a pre-piloted data extraction format prepared in the Microsoft™ Excel spreadsheet. Disagreement in data abstraction between the first two and third authors resolved by a fourth independent author (BWA). From each observational study, we have extracted data participant sex, study year, region, sample size, study design, and first author name for the proportion of ischemic stroke, hemorrhagic stroke, improvement at discharge, and each risk factors (hypertension, diabetes mellitus, alcohol consumption, smoking, heart disease, lack physical activities, cholesterol, and obesity).

Before analysis, prevalence transformation carries out. The Newcastle-Ottawa Scale (NOS) was used to select for assessing the quality of the included studies. The NOS had three categorical criteria with a maximum score of ten points. The assessment tool contains representatives of the sample, sample size, non-respondents, and ascertainment of exposure, independent blind assessment, and statistical test. Based on NOS, a score of 6 out of 10 consider as good quality. To maintain the validity of this review, we only included primary studies with fair to good quality (17-19).

The primary outcome of this review was the pooled proportion of the overall ischemic, hemorrhagic, and survival rate discharge and the magnitude of common modifiable risk factors among stroke patients in Ethiopia. Stroke is rapidly developing clinical signs of focal, or at times, global disturbance of

cerebral function, lasting more than 24 hours or leading to death with no apparent cause other than a vascular origin (20, 21).

Quality Assessment

The 10-items rating scale uses to assess for each observational study (22). The assessment tool has a representative sample size, data collection method, reliability, and validity of study tools, case definition, and prevalence periods of the studies. Researchers categorized each observational article study as having a low risk of bias ("yes" answers to domain questions) or a high risk of bias ("no" answers to domain questions). Each study assigned a score of 1 (Yes) or 0 (No) for each domain, and these domain scores ~~sum~~ to give an overall study quality score. Scores of 8–10 ~~consider~~ as having a "low risk of bias," 6–7 a "moderate risk," and 0–5 a "high risk." For the least risk of bias classification, discrepancies between the reviewers resolved via consensus.

Data analysis

Heterogeneity test and publication bias

Heterogeneity between the findings of the primary studies was assessed by using Cochran's Q test and quantified with the I-square statistics. A P-value of less than 0.1 considers suggesting statistically significant heterogeneity. A heterogeneity considering a small number of studies and their heterogeneity in design (23). Heterogeneity classifications are: when I-square values below 25% low, 25-75% moderate, and above 75% high (24). The random-effect model was used to pool the recommended health life rate, accounts for heterogeneity among study results beyond the variation associated with the chance (25).

We used the random-effect model to investigate the source of heterogeneity. The meta-analysis was weighted to account for the residual between-study heterogeneity (i.e., heterogeneity not explained by the covariate in the regression (26). Publication bias assesses by visual inspection

of funnel plots based on the shape of the graph. The symmetrical graph interprets as an absence of publication bias. An asymmetrical one indicated the presence of publication bias.

We employed Begg's and Egger's weighted regression to identify the source of publication bias. P-values less than 0.05 were considered as the presence of significant publication bias (27, 28). We also applied leave-out sensitivity analysis to confirm some studies potentially biased the direction of the pooled estimate. Subgroup analyses by region and type of study setup (hospitals) carried out because of significant heterogeneity between studies (i.e., I^2 = 96.5%, p<0.05)

Statistical analysis

Data analyzed in Stata Version 14. Data was presented in the evidence table and summarized using descriptive statistics. The effect measure for outcome variables computed using the "Metaprop" command for meta-analysis of the proportion in Stata. In this review, the overall burden of stroke, rate of improvement, and common modifiable risk factors ~~calculate~~ together with their corresponding 95% CI. A forest plot generates to show the individual pooled burden of strokes and common modifiable stroke 95% CI, the author's name, study year, and study weights.

Result

Study selection process

From electronic databases, we retrieved 986 observational studies. After screening their titles and abstracts, duplications removed using Endnote X7. ~~Accordingly, we removed 644 duplicate articles.~~ Of 342 articles, 315 articles ~~exclude~~ because their titles and abstracts were not in line with our inclusion criteria (full article not found, different population, different setting, and different outcome). Finally, 27 articles include for this systemic review and meta-analysis (Fig. 1).

Study characteristics

Overall, we selected a total of 27 observational studies in this systematic review and meta-analysis. We included a total of 5,845 participants. Among them, 2,647 participants were male, and 3,228 participants improved at the time of discharge. The number of participants in each study ranged from 73 to 503. The most retrieved studies (n = 8) were from the Oromia (29-36) region followed by Addis Ababa (n=7) (37-43) and Amhara region (n=7) (44-50), Tigray region represented by four studies (51-54), Southern Nations Nationalities and People's (SNNP) (55) region had one study.

The smallest sample size was 73 obtained from a study conducted at Shashemene Referral Hospital, Ethiopia (29). The largest sample size was 503 reported from a study done at Ayder Comprehensive Specialized Hospital, Northern Ethiopia (52). Most studies dealt with hypertension as a modifiable risk factors of stroke (n=24) (29-34, 36, 37, 39-41, 43, 44, 48, 51-59) followed by Arterial Fibrillation (AF) (n = 17) (30-32, 37, 40, 41, 44, 45, 47-50, 53-55, 57, 58), Diabetes mellitus (DM) (n = 15) (30, 31, 33, 37, 39-41, 44, 48, 49, 55-59), other heart disease rather than AF (n = 14) (29, 30, 32, 37, 41, 43, 47, 49, 54-59), and high cholesterol level (n = 7) (32, 33, 35, 37, 44, 48, 49, 51, 55) (Table1).

Table 1: Study characteristics of included articles for the final systematic review and meta-analysis on the burden of modifiable risk factors and rate of improvement at discharge after stroke in Ethiopia 2020

Authors name	Study year	Region	Sample size	Study design	Burden of Stroke reported outcome percentage (95% CI)					
					Ischemic stroke	Hemorrhagic stroke	Female	Male	Improvement at discharge	NOS score
Asgedome SW.et al	2019	Tigray	216	R	55.6	44.4	58.3	41.7	77.8	8
Asres AK. et al	2018	AA	170	CC	51.2	37.6	42.9	57.1	72.4	7
Baye M. et al	2018	Amhara	448	R	31.5	68.5	58.0	42.0	59.8	8

Bedassa T. et al	2018	Oromia	242	R	64.3	35.7	no	Not	Not	5
Beyene DT. et al	2017	Oromia	367	R	35.7	64.31	36.2	63.8	26.4	8
Dandena A. et al	2019	Oromia	283	P	43.1	44.5	35.0	65.0	Not	6
Deresse B. et al	2014	SNNP	163	P	50.3	49.7	33.7	66.3	85.3	8
Erkabu SG. et al	2016	Amhara	303	R	59.4	40.6	37	63.0	89.0	7
Fekadu G.et al	2017	Oromia	116	P	51.7	48.3	37.1	62.9	Not	6
Fekadu G.et al	2017	Oromia	116	CC	48.3	41.6	37.1	62.9	78.4	8
Fekadu G.et al	2017	Oromia	364	CC	42.3	57.7	42.9	57.7	94.0	7
Gebremariam SA. et al	2014	Tigray	142	CC	55.6	38.0	45.8	54.2	47.9	8
Gebreyohannes EA.et al	2017	Amhara	208	R	57.7	Not	57.7	42.3	87.5	7
Gedefa B. et al	2016	AA	163	R	35.6	64.4	43.6	56.4	69.9	8
Gelan Y. et al	2016	AA	227	CC	49.8	48.9	30.0	70.0	70.0	7
Greffie. ES et al	2013	Amhara	98	R	69.4	30.6	53.1	46.9	87.0	7
Gufue ZH. et al	2019	Tigray	503	R	56.6	43.4	50.1	49.9	85.1	7
Kassaw A.et al	2018	AA	170	R	51.2	48.8	42.9	57.1	80.0	8
Kefale B. et al	2019	Oromia	111	R	80.1	18.0	50.5	49.5	83.8	7
Mekonen HH.et al	2018	Tigray	89	R	32.6	36.6	63.2	51.7	Not	5
Mulat B. et al	2015	Amhara	427	R	56.7	43.3	63.2	36.8	Not	6
Mulugeta H. et al	2019	Amhara	162	R	50.0	30.0	53.7	46.3	27.2	7
Sultan M. et al	2014	AA	301	p	53.8	17.9	42.5	57.5	80.7	8
Tamirat KS. et al	2017	Amhara	151	R	60.3	39.7	50.3	49.7	90.7	7
Temesgen TG.et al	2017	Oromia	73	R	65.8	34.2	42.5	57.5	54.8	6
Zenebe G. et al	2001	AA	128	CC	43	57.0	39.8	61.7	Not	6
Zewdie A. et al	2016	AA	104	CC	44.2	55.8	44.0	56.0	Not	5

R: Retrospective, P: Prospective, CC: Cross-Sectional, AA: Addis Ababa, NOS: Newcastle-Ottawa Scale

Quality Appraisal

The quality score of the included study ranged from 5 to 8 to a mean score of 7.04 (SD = 0.94). Out of 27 studies, 21 (77.78 %) studies received a low risk of bias. Five studies (29-31, 36,

39, 44, 49, 51, 55) had a high risk of case definition, five studies (29, 30, 35, 55, 56) had random selection bias, and 14 studies (29, 33-35, 39, 41, 47, 51, 53, 57, 58) had a high risk of representation bias (S2.).

The magnitude of strokes in Ethiopia

From the total rank of twenty-seven primary studies, twenty-five, twenty-six studies provided information on the proportion of hemorrhagic and ischemic stroke. Twenty-six studies also provide information on stroke proportion in females and males. Twenty primary studies reported the rate of improvement at discharge after stroke. As presented in the forest plot (Fig. 2 and 3), the pooled estimate proportion of hemorrhagic and ischemic stroke were 46.42% (95%CI: 41.82-51.53; I^2 = 91.6%) and 51.40% (95%CI: 46.97- 55.82; I^2 = 85.5%) respectively. The pooled estimate of stroke among female was 45.07 % (95%CI: 41.80-48.35; I^2 = 80.3%) and male was 54.70 % (95%CI: 51.32-58.08; I^2 = 79.5%) (S3.).

The magnitude of modifiable risk factors of stroke in Ethiopia

We investigated the magnitude of modifiable risk factors of stroke among the included studies. The proportion of DM among stroke patients ranged from 5.2% (54) to 21.6% (39). A total of 3356 stroke patients include determining the pooled magnitude of DM among stroke patients. Accordingly, our pooled analysis showed that 14.722% (95%CI: 9.51, 19.94; I^2 = 95.8) of stroke patients had DM. In this review, stroke patients who had hypertension ranged from 24.1% (33) to 75.2% (32).

We used a total of 5064 stroke patients to determine the pooled magnitude of hypertension risk factors in stroke patients. Consequently, we found that the overall pooled estimation of hypertension among stroke patients was 49% (95CI%:43.59, 54.41; I^2 = 91.6%). Furthermore, the proportion of alcohol consumption (more than two drinks in a day for men and more than one

drink in a day for women) among stroke patients included in this study ranged from 10.4% (55) to 41.4% (48). Our meta-analysis revealed that 24.96 % (95%CI: 15.01, 34.90; I^2 = 92.7) of stroke patients had a history of harmful alcohol intake (Table 2).

Table 2: The pooled effect of common modifiable risk factors among the primary studies of stroke in Ethiopia.

**Modifiable risk factors**	**Estimated pooled proportion (95%CI)**	**I-squared (%)**
Hypertension	49 (43.59, 54.41)	91.6
Diabetes mellitus	14.72 (9.51, 19.94)	95.8
Atrial fibrillation	19.21 (13.96, 24.46)	94.4
Other heart disease	20.11 (14.27, 25.95)	94.2
Dyslipidemia	20.99 (11.10, 30.88)	96.4
Smoking	10.38 (6.27, 14.94)	86.0
Obesity	11.64 (2.48, 20.79)	95.3
Alcohol	24.96 (15.01, 34.90)	92.7

Other heart Disease: Congestive heart failure, Structural heart disease, Myocardia friction

Recovery from stroke in Ethiopia

The proportion of improvement during discharge after stroke among the included primary studies was ranged from 26.4% (30) to 94% (36). We used 2321 stroke patients to estimate the pooled proportion of improvement at the time of discharge. The pooled improvement status of stroke during discharge in Ethiopia was 72.28% (95%CI: 62.48, 82.08; I^2 = 96.5%) (Fig. 4).

Publication bias

Both funnel plots of precision asymmetry and Egger's intercept test showed no publication bias in the primary studies. Visual examination of the funnel plot showed the symmetric distribution. Additionally,

Egger's intercept test o was -0.147 (95% CI: -0.26, 1.18) $p > 0.05$ (0.102), as judged by Egger's test, there was no evidence of publication bias present at a 5% significance level (Fig. 5).

Subgroup analysis

Due to the heterogeneity of included studies, we performed a subgroup analysis using the following study characteristics: region, sample size, and study year. We applied the random-effect model for reporting the pooled proportion of clinical outcomes during discharge of the subgroup analysis. Accordingly, the highest recovery rate observed from the Addis Ababa region 74.51 (69.84-79.17; I^2 = 34.5%). The least pooled proportion of recovery was in the Oromia region (67.38 (95%CI: 41.60-93.17; I^2 = 98.1%). The subgroup analysis by study year showed that the pooled proportion of recovery rate after stroke during discharge was 75.59 % (95%CI 64.28 - 86.9; I^2 = 92.1%) for studies conducted before 2017 (Table 3).

Table 3: Subgroup analysis of recovery after stroke by region, sample size, and study year in Ethiopia 2020.

Variables	Characteristics	Estimated stroke recover during discharge (95% CI; I^2 = %)
	Oromia	67.38 (41.60-93.17; I^2 = 98.1)
	Addis Ababa	74.51 (69.84-79.17; I^2 = 34.5)
Region	Amhara	73.44 (50.29 – 96. 59; I^2 = 97.9)
	Tigray	70.19 (47.10 - 93.28; I^2 = 95.7)
	SNNPR	Single study
Sample size	<223 (median)	72.41 (60.00 - 83.82; I^2 = 96.0)
	>=223 (median)	72.05 (527.44 - 91.66; I^2 = 97.5)
Study year	Before 2017	75.59 (64.28 - 86.9; I^2 = 92.1)

After 2017	70.50 (56.80 - 84.20; I^2 = 92.1)

SNNPR: South Nations, Nationalities and People Region

Meta-regression and sensitivity analysis

The subgroup analysis showed that heterogeneity across the studies was widespread. To ~~name~~ the source of heterogeneity, we conducted a meta-regression and sensitivity analysis. During the meta-regression analysis, we applied ~~using~~ the following study covariance: study years and region. However, the results showed that none of these variables were a statistically significant source of heterogeneity. We also performed a sensitivity analysis to find the influence of each study on the overall effect size. No single study affected the overall pooled proportion of clinical outcomes of stroke among stroke patients in Ethiopia (Table 4, Fig. 6).

Table 4: Meta-regression output to explore the heterogeneity of the pooled proportion of clinical outcome of stroke in Ethiopia, 2020.

Variables	Coefficients	P-value	95% CI
Study Year	-4.83	0.538	-21.03, 11.35
Region			
Addis Ababa	-10.68	0.660	-61.37, 40.01
Amhara	-11.67	0.626	-61.63, 38.29
Oromia	-17.72	0.468	-68.44 33.01
Tigray	-14.83	0.563	-68.26 38.61

Discussion

This study aimed to determine the overall proportion of stroke burden and modifiable risk factors in Ethiopia. Of all stroke cases in our review, more than half (51.40%) of stroke patients

in Ethiopia was an ischemic stroke. This finding was almost similar to a study done in Kenya (56.1%) (60), much lower than a study done in China (81.9- 91.7%) (61, 62), Burkina Faso (61.63%) (63), Iran (76.5 - 81.9%) (64, 65), and a 22 country case-control study (78%) (13). The difference in culture and economic status, lifestyle difference, poor management of modifiable risk factors, and difference in the preventive strategies in the general public could be the reasons for the difference.

For all stroke cases, males stroke patients (54.70%) are relatively higher prevalence than females stroke patients (45.07%) by a 54.70: 45.07 split of male and female in all recruited patients in Ethiopia. A systematic review of epidemiological studies on Western European surveys has shown that stroke is more common in males than in females (66). The gender difference might be males present a higher risk of stroke than female[illegible]ut females suffer from a stroke at older ages making them more prone to die from stroke than are males. Females were risk factor avoidance than males. Such as females are less likely to drink heavily or smoke than males (67).

Our meta-analysis showed that almost half (49%) of all stroke patients had hypertension. Previous evidence also showed that 75.8 % of stroke patients had hypertension (63), hypertensive individuals are two to four times more likely to have a stroke (13, 61, 68). Hypertension has remained the leading modifiable risk factors of stroke morbidity and mortality since 1990 (69). People who can maintain normal blood pressure can decrease the risk of stroke by 30 to 40% (70). Though hypertension is the main reported modifiable risk factor of stroke among the included primary studies, the pooled proportion of hypertension among stroke patients found in the current study is lower than the previous studies conducted in Burkina Faso (63), Iran (65), China (62), Bosnia-Herzegovina (71), Nigeria (72), and Bangladesh (73). The possible explanation for this

variation might be due to the lack of diagnostic modalities and proficiency, level of income, hypertension awareness, treatment, and control (74).

Above limit, alcohol consumption is a well-established risk factor of stroke. In our review, alcohol consumption is the second most common modifiable risk factor of stroke. Almost one-fourth (24.96%) of stroke patients had a history of alcohol consumption. Because ~~drinking the upper limit of~~ alcohol intake can trigger atrial fibrillation – a type of irregular heartbeat. Atrial fibrillation increases the risk of stroke by five times because it can cause blood clots to form in the heart. If these clots move up into the brain, it can lead to stroke (75).

A review of 84 studies of alcohol consumption and cardiovascular disease, alcohol consumption >60 g/day increased the risk of incident stroke by 62% compared ~~with abstaining~~ from alcohol (76). The pooled proportion of alcohol consumption among stroke patients in Ethiopia ~~is~~ higher than a study conducted in Nigeria (72). The possible explanation for this variation might be the lack of diagnostic modalities and proficiency; measured dyslipidemia in the medical record before the occurrence of stroke. Another reason for this variation, ~~there is no~~ effective community action to control alcohol consumption in Ethiopia (77, 78).

In our ~~current~~ study, dyslipidemia is the third most common modifiable risk factor of stroke. More than two-tenth (20.99%) of stroke patients had dyslipidemia. ~~Because of this, dyslipidemia~~ promotes cervical or coronary atherosclerosis, which predisposes to athero-thrombotic and cardio-embolic stroke (79). Our review is comparable with a previous study conducted in Nigeria (72). However, this estimated proportion of dyslipidemia among stroke patients is lower than a study conducted in Bosnia-Herzegovina (71), China (62).

The reasons for the above results could ~~attribute~~ to the following: first, the dramatic ~~shift of~~ increases in the prevalence of many known risk factors for chronic diseases such as unhealthy

lifestyles (decreased physical activity, smoking, alcohol consumption, and ~~familiarize~~ westernized diet) (80, 81). Second, the impact of rapid urbanization (increased risk of obesity) (82). In Nigeria, dyslipidemia in stroke patients is closely linked to ~~a~~ western diet ~~and saturated with fast food outlets~~ and physically inactive lifestyle behaviors (83).

Diabetes is a well-established risk factor for stroke, and our analysis showed that diabetes mellitus is the fourth most common comorbidity of stroke. More than one-tenth (14.72%) of stroke patients had diabetes mellitus. ~~Because of this,~~ diabetes causes various micro-vascular and macro-vascular changes ending in major clinical complications (84). ~~This finding is comparable with~~ the previous study conducted in Nigeria (72); higher than the ~~study~~ done in Burkina Faso (63), sub-Saharan African (85), and a systematic review and meta-analysis done in Ethiopia (86). However, this proportion of diabetes mellitus among stroke patients is lower than a study done in Bosnia-Herzegovina (71) and Iran (65).

The result found in this study showed that the overall pooled estimated proportion of recovery after stroke in Ethiopia was 72.28% (95%CI: 62.48, 82.08). This finding is in line with the ideal proportional recovery rule of stroke (87-89) but still lower than the goal set for 2015 (85%) in the Helsingborg Declaration 2006 (90). Even though recovery after stroke is in line with the ideal recovery rule of stroke, this result is still lower than the previous study conducted in Bosnia-Herzegovina (71), Iran (65), sub-Saharan Africa (85), and Kenya (60). The possible reason might be ~~the~~ management protocol for stroke patients ~~was sub-optimal and lagging behind the recommended guidelines due to a~~ lack of skilled personnel, appropriate treatment, and diagnostic agents in Ethiopia (91). However, this result is relatively higher than previous studies conducted in Ghana (92).

The subgroup analyses by year of studies showed that the overall pooled proportion of recovery rate after stroke was higher among studies conducted before 2017. The lowest pooled proportion of recovery rate of stroke in this study population after ~~20717~~ may reflect the increased exposure to risk factors for stroke due to ongoing epidemiological and demographic transitions.

Limitations

There is considerable heterogeneity across the included studies. The observed heterogeneity may ~~describe by~~ differences in the study design, the quality of the studies, and sensitivity. Since our study focused on in-patient, it cannot externally validate to the general population.

Implication

This study has many implications for clinical practice and future research. First, the health care provider (physicians and nurses) can develop effective strategies to improve recovery after stroke and all modifiable stroke like high blood pressure, alcohol consumption behaviors that stroke patients exhibit such modifiable stroke risk factors. Second, ~~identify the restriction to modify~~ these modifiable ~~stroke~~ risk factors is the first step in developing evidence-based interventions to promote short and long-term health outcomes and quality of life. Future research should focus on developing and testing a conceptual model that can use accessibility to screening, treatment, sociocultural aspects of stroke risk factor modification in a national context. Finally, to give a long-term reduction in stroke risk factor-related co-morbidity, researchers should assess ways to extend and sustain lifestyle modifiable stroke risk factors and recovery rate after ~~a~~ stroke ~~of~~ this population.

Conclusion

There is a high burden of stroke with a high rate of modifiable risk factors in Ethiopia. More than 90% of patients had one or more modifiable risk factors. Therefore, efforts focused on the primary prevention of stroke. ~~Such as lower high blood pressure, alcohol limitation or moderation,~~

early screening and treatment of atrial fibrillation and diabetes, lose weight, exercise more, and quit smoking.

Declarations

Abbreviations

DALYs: Disability-Adjusted Life-Years; NCDs: Non-Communicable Diseases; MESH: Medical Subject Headings; SNNPR: South Nations, Nationalities and People Region; NOS: Newcastle-Ottawa Scale

Ethics approval and consent to participate

Not applicable.

Consent for publication

Not applicable.

Acknowledgments

Not applicable

Reference

1. Gorelick PBJTLN. The global burden of stroke: persistent and disabling. 2019;18(5):417-8.

2. Johnson CO, Nguyen M, Roth GA, Nichols E, Alam T, Abate D, et al. Global, regional, and national burden of stroke, 1990–2016: a systematic analysis for the Global Burden of Disease Study 2016. 2019;18(5):439-58.

3. Yan LL, Li C, Chen J, Miranda JJ, Luo R, Bettger J, et al. Prevention, management, and rehabilitation of stroke in low-and middle-income countries. J Eneurologicalsci. 2016;2:21-30.

4. Mukherjee D, Patil CG. Epidemiology and the global burden of stroke. World neurosurgery. 2011;76(6):S85-S90.

5. Katan M, Luft A, editors. Global burden of stroke. Seminars in neurology; 2018: Georg Thieme Verlag.

6. Kaba MJEJoHD. Non-communicable diseases: unwelcome in Ethiopia. 2018;32(3).

7. Misganaw A, Haregu TN, Deribe K, Tessema GA, Deribew A, Melaku YA, et al. National mortality burden due to communicable, non-communicable, and other diseases in Ethiopia, 1990–2015: findings from the Global Burden of Disease Study 2015. Population health metrics. 2017;15(1):29.

8. BeLue R, Okoror TA, Iwelunmor J, Taylor KD, Degboe AN, Agyemang C, et al. An overview of cardiovascular risk factor burden in sub-Saharan African countries: a socio-cultural perspective. 2009;5(1):10.

9. Feigin VL, Roth GA, Naghavi M, Parmar P, Krishnamurthi R, Chugh S, et al. Global burden of stroke and risk factors in 188 countries, during 1990–2013: a systematic analysis for the Global Burden of Disease Study 2013. 2016;15(9):913-24.

10. Jones P, Jones DJNT. Primary and Secondary Stroke Prevention Strategies. 2017;113(12):42-6.

11. Johnston SC, Mendis S, Mathers CDJTLN. Global variation in stroke burden and mortality: estimates from monitoring, surveillance, and modelling. 2009;8(4):345-54.

12. Mayosi BM, Lawn JE, Van Niekerk A, Bradshaw D, Karim SSA, Coovadia HM, et al. Health in South Africa: changes and challenges since 2009. 2012;380(9858):2029-43.

13. O'donnell MJ, Xavier D, Liu L, Zhang H, Chin SL, Rao-Melacini P, et al. Risk factors for ischaemic and intracerebral haemorrhagic stroke in 22 countries (the INTERSTROKE study): a case-control study. 2010;376(9735):112-23.

14. Gebremariam LW, Chiang C, Yatsuya H, Hilawe EH, Kahsay AB, Godefay H, et al. Non-communicable disease risk factor profile among public employees in a regional city in northern Ethiopia. 2018;8(1):1-11.

15. Shiferaw F, Letebo M, Misganaw A, Feleke Y, Gelibo T, Getachew T, et al. Non-communicable Diseases in Ethiopia: Disease burden, gaps in health care delivery and strategic directions. 2018;32(3).

16. Moher D, Liberati A, Tetzlaff J, Altman DG, med PGJP. Preferred reporting items for systematic reviews and meta-analyses: the PRISMA statement. 2009;6(7):e1000097.

17. Liberati A, Altman DG, Tetzlaff J, Mulrow C, Gøtzsche PC, Ioannidis JP, et al. The PRISMA statement for reporting systematic reviews and meta-analyses of studies that evaluate health care interventions: explanation and elaboration. Journal of clinical epidemiology. 2009;62(10):e1-e34.

18. Modesti PA, Reboldi G, Cappuccio FP, Agyemang C, Remuzzi G, Rapi S, et al. Panethnic differences in blood pressure in Europe: a systematic review and meta-analysis. PloS one. 2016;11(1):e0147601.

19. . Newcastle-Ottawa Scale customized for cross-sectional studies In. available from https://static-content.springer.com/esm/.../12889_2012_5111_MOESM3_ESM.doc.

20. WHO. WHO STEPS stroke manual: the WHO STEPwise approach to stroke surveillance: World Health Organization; 2005.

21. Sacco RL, Kasner SE, Broderick JP, Caplan LR, Connors J, Culebras A, et al. An updated definition of stroke for the 21st century: a statement for healthcare professionals from the American Heart Association/American Stroke Association. 2013;44(7):2064-89.

22. Hoy D, Brooks P, Woolf A, Blyth F, March L, Bain C, et al. Assessing risk of bias in prevalence studies: modification of an existing tool and evidence of interrater agreement. Journal of clinical epidemiology. 2012;65(9):934-9.

23. Higgins JP, Thompson SG, Deeks JJ, Altman DG. Measuring inconsistency in meta-analyses. Bmj. 2003;327(7414):557-60.

24. Cumpston M, Li T, Page MJ, Chandler J, Welch VA, Higgins JP, et al. Updated guidance for trusted systematic reviews: a new edition of the Cochrane Handbook for Systematic Reviews of Interventions. Cochrane Database Syst Rev. 2019;10:ED000142.

25. Borenstein M, Hedges LV, Higgins JP, Rothstein HR. A basic introduction to fixed- effect and random- effects models for meta- analysis. Research synthesis methods. 2010;1(2):97-111.

26. Thompson SG, Higgins JP. How should meta- regression analyses be undertaken and interpreted? Statistics in medicine. 2002;21(11):1559-73.

27. Duval S, Tweedie R. A nonparametric “trim and fill” method of accounting for publication bias in meta-analysis. Journal of the american statistical association. 2000;95(449):89-98.

28. Egger M, Smith GD, Schneider M, Minder C. Bias in meta-analysis detected by a simple, graphical test. Bmj. 1997;315(7109):629-34.

29. Temesgen TG, Teshome B, Njogu PJSr, treatment. Treatment outcomes and associated factors among hospitalized stroke patients at Shashemene Referral Hospital, Ethiopia. 2018;2018.

30. Beyene D, Asefa H. A two year retrospective cross-sectional study on prevalence, associated factors and treatment outcome among patients admitted to medical ward (stroke unit) at Jimma University Medical Center, Jimma, South West, Ethiopia, 2018. J Palliat Med Care. 2018;5(4):1-6.

31. Kefale B, Betero G, Temesgen G, Degu A. Management practice, and treatment outcome and its associated factors among hospitalized stroke patient at Ambo University Referral Hospital, Ethiopia: an Institutional Based Cross Sectional Study. 2019.

32. Fekadu G, Chelkeba L, Kebede AJBn. Risk factors, clinical presentations and predictors of stroke among adult patients admitted to stroke unit of Jimma university medical center, south west Ethiopia: prospective observational study. 2019;19(1):1-11.

33. Bedassa TSJIR. Assessment of the types and factors associated with stroke among adult patients admitted in Adama Hospital Medical College, Ethiopia. 2019;6:S490.

34. Dandena A, Sinaga M, Yirga Y, Zelalem T. CT Scan Pattern of Stroke Patients at Jimma University Medical Center, South West Ethiopia. J Biomedical Journal of Scientific Technical Research. 2020;29(4):22652-7.

35. Fekadu G, Chelkeba L, Kebede AJBn. Burden, clinical outcomes and predictors of time to in hospital mortality among adult patients admitted to stroke unit of Jimma university medical center: a prospective cohort study. 2019;19(1):213.

36. Fekadu G, Adola B, Mosisa G, Shibiru T, Chelkeba L. Clinical characteristics and treatment outcomes among stroke patients hospitalized to Nekemte referral hospital, western Ethiopia. Journal of Clinical Neuroscience. 2020;71:170-6.

37. Sultan M, Debebe F, Azazh A, Hassen GWJEJoHD. Epidemiology of stroke patients in Tikur Anbessa Specialized Hospital: Emphasizing clinical characteristics of hemorrhagic stroke patients. Ethiop J Health Dev. 2017;31(1):13-7.

38. Gedefa B MT, Berhe T, Abera H. Assessment of risk factors and treatment outcome of stroke admissions at St. Paul's teaching hospital, addis ababa, Ethiopia. Neurophysiology. 2017;8:1-6.

39. Zewdie A DF, Kebede S, Azazh A, Laytin A, Pashmforoosh G. et al. Prospective assessment of patients with stroke in Tikur Anbessa specialised hospital, Addis Ababa, Ethiopia. African Journal of Emergency Medicine. 2018;8(1):21-4.

40. Asres AK CA, Bedada T, Gebrekidan H. Frequency, nursing managements and stroke patients' outcomes among patients admitted to Tikur Anbessa specialized hospital, Addis Ababa, Ethiopia a retrospective, institution based cross-sectional study. International Journal of Africa Nursing Sciences. 2020: Jan 1;13:100228.

41. Ayehu Kassaw AC, Tadesse Bedada. Prevalence, nursing managements and patients' outcomes among stroke patients admitted to Tikur Anbessa specialized hospital, Addis Ababa, Ethiopia, 2018: Addis Ababa University; 2018.

42. Zenebe G, Alemayehu M, Asmera JJEmj. Characteristics and outcomes of stroke at Tikur Anbessa Teaching Hospital, Ethiopia. 2005;43(4):251-9.

43. Gelan Y, Weldeab A. Predictors of Stroke Mortality among Patients Admitted to a Hospital in Ethiopia (2070). AAN Enterprises; 2020.

44. Erkabu SG AY, Mihretu DD, Semere A, Alemu YM. Ischemic and hemorrhagic stroke in Bahir Dar, Ethiopia: a retrospective hospital-based study. Journal of Stroke and Cerebrovascular Diseases. 2018;27(6):1533-8.

45. Gebreyohannes EA BA, Abebe TB, Seid MA, Haile KT. In-hospital mortality among ischemic stroke patients in Gondar University Hospital: a retrospective cohort study. Stroke research and treatment. 2019;2019.

46. Greffie ES MT, Getahun S. Risk factors, clinical pattern and outcome of stroke in a referral hospital, Northwest Ethiopia. Clin Med Res. 2015;4(6):182-8.

47. Baye M HA, Gordon-Murer C, Mariscal T, Belay GJ, Gebremariam AA et al. Stroke Characteristics and Outcomes of Adult Patients in Northwest Ethiopia. Frontiers in Neurology. 2020;11:428.

48. Mulugeta H, Yehuala A, Haile D, Mekonnen N, Dessie G, Kassa GM, et al. Magnitude, risk factors and outcomes of stroke at Debre Markos Referral Hospital, Northwest Ethiopia: a retrospective observational study. The Egyptian Journal of Neurology, Psychiatry and Neurosurgery. 2020;56:1-9.

49. Mulat B MJ, Yeseni M, Alamirew M, Dermello M, Asemahagn MA. Magnitude of stroke and associated factors among patients who attended the medical ward of Felege Hiwot Referral Hospital, Bahir Dar town, Northwest Ethiopia Ethiopian Journal of Health Development. 2016;30(3):129-34.

50. Tamirat KS AS, Gebi NB, Gerffie ES. Clinical profile, in-hospital outcome and associated factors of stroke after the start of a standard organized stroke care unit at university of Gondar hospital, northwest Ethiopia. 2019. p. 15.

51. Mekonen HH, Birhanu MM, Mossie TB, Gebreslassie HT. Factors associated with stroke among adult patients with hypertension in Ayder Comprehensive Specialized Hospital, Tigray, Ethiopia, 2018: A case-control study. J PloS one. 2020;15(2):e0228650.

52. Gufue ZH GN, Ayele W, Yifru YM, Hailu NA, Welesemayat ET et al Survival of Stroke Patients According to Hypertension Status in Northern Ethiopia: Seven Years Retrospective Cohort Study. Vascular Health and Risk Management 2020;16:389.

53. Gebremariam SA, Yang HS. Types, risk profiles, and outcomes of stroke patients in a tertiary teaching hospital in northern Ethiopia. J Eneurologicalsci. 2016;3:41-7.

54. Asgedom SW, Gidey K, Gidey K, Niriayo YL, Desta DM, Atey TM. Medical complications and mortality of hospitalized stroke patients. J Journal of Stroke Cerebrovascular Diseases. 2020;29(8):104990.

55. Deresse B, Shaweno DJJotns. Epidemiology and in-hospital outcome of stroke in South Ethiopia. 2015;355(1-2):138-42.

56. Gebreyohannes EA, Bhagavathula AS, Abebe TB, Seid MA, Haile KTJSr, treatment. In-hospital mortality among ischemic stroke patients in Gondar University Hospital: a retrospective cohort study. 2019;2019.

57. Gedefa B, Menna T, Berhe T, Abera H. Assessment of risk factors and treatment outcome of stroke admissions at St. Paul's teaching hospital, addis ababa, Ethiopia. Journal of Neurology Neurophysiology. 2017;8(3):1-6.

58. Greffie ES, Mitiku T, Getahun S. Risk factors, clinical pattern and outcome of stroke in a referral hospital, Northwest Ethiopia. J Clin Med Res. 2015;4(6):182-8.

59. Tamirat K, Abdella S, Gebi N, Gerffie E. Clinical profile, in-hospital outcome and associated factors of stroke after the start of a standard organized stroke care unit at university of Gondar hospital, northwest Ethiopia. 2019.

60. Kaduka L, Muniu E, Oduor C, Mbui J, Gakunga R, Kwasa J, et al. Stroke mortality in Kenya’s public tertiary hospitals: a prospective facility-based study. 2018;8(2):70-9.

61. Yi X, Luo H, Zhou J, Yu M, Chen X, Tan L, et al. Prevalence of stroke and stroke related risk factors: a population based cross sectional survey in southwestern China. 2020;20(1):5.

62. Zhang F-L, Guo Z-N, Wu Y-H, Liu H-Y, Luo Y, Sun M-S, et al. Prevalence of stroke and associated risk factors: a population based cross sectional study from northeast China. 2017;7(9):e015758.

63. Samadoulougou DRS, Kpoda H, Traore I, Savadogo L, Sombie I, Millogo A, editors. Evolution of the magnitude of stroke at the teaching hospital of Bobo-Dioulasso. World Congress on Clinical Trials in Diabetes; 2016: BioScientifica.

64. Ghandehari KJGMJ. Epidemiology of stroke in Iran. 2016;5(S1):3-9.

65. Farhoudi M, Mehrvar K, Sadeghi-Bazargani H, Hashemilar M, Seyedi-Vafaee M, Sadeghi-Hokmabad E, et al. Stroke subtypes, risk factors and mortality rate in northwest of Iran. 2017;16(3):112.

66. Appelros P, Stegmayr B, Terént AJS. Sex differences in stroke epidemiology: a systematic review. 2009;40(4):1082-90.

67. Yao X-y, Lin Y, Geng J-l, Sun Y-m, Chen Y, Shi G-w, et al. Age-and gender-specific prevalence of risk factors in patients with first-ever ischemic stroke in China. 2012;2012.

68. Chobanian AV, Bakris GL, Black HR, Cushman WC, Green LA, Izzo Jr JL, et al. Seventh report of the joint national committee on prevention, detection, evaluation, and treatment of high blood pressure. hypertension. 2003;42(6):1206-52.

69. Avan A, Digaleh H, Di Napoli M, Stranges S, Behrouz R, Shojaeianbabaei G, et al. Socioeconomic status and stroke incidence, prevalence, mortality, and worldwide burden: an ecological analysis from the Global Burden of Disease Study 2017. 2019;17(1):191.

70. Lawes CM, Bennett DA, Feigin VL, Rodgers AJS. Blood pressure and stroke: an overview of published reviews. 2004;35(3):776-85.

71. Bender M, Jusufovic E, Railic V, Kelava S, Tinjak S, Dzevdetbegovic D, et al. High burden of stroke risk factors in developing country: the case study of Bosnia-Herzegovina. 2017;29(4):277.

72. Watila M, Ibrahim A, Balarabe S, Gezawa I, Bakki B, Tahir A, et al. Risk factor profile among black stroke patients in Northeastern Nigeria. 2012;4(5):50-8.

73. Hossain A, Ahmed N, Rahman M, Islam M, Sadhya G, Fatema KJFMCJ. Analysis of sociodemographic and clinical factors associated with hospitalized stroke patients of Bangladesh. 2011;6(1):19-23.

74. Mills KT, Bundy JD, Kelly TN, Reed JE, Kearney PM, Reynolds K, et al. Global disparities of hypertension prevalence and control: a systematic analysis of population-based studies from 90 countries. 2016;134(6):441-50.

75. Alcohol and stroke, Factsheet13 (2014).

76. Paul E Ronksley SEB, Barbara J Turner, Kenneth J Mukamal, William A Ghali. Association of alcohol consumption with selected cardiovascular disease outcomes: a systematic review and meta-analysis. BMJ. 2011:342:d671.

77. Beyene NJJophp. Alcohol control policy in Ethiopia and implications for public health. 2019;40(4):423-35.

78. Getachew T, Defar A, Teklie H, Gonfa G, Bekele A, Bekele A, et al. Magnitude and predictors of excessive alcohol use in Ethiopia. 2017;31(1).

79. Ayata C, Shin HK, Dileköz E, Atochin DN, Kashiwagi S, Eikermann-Haerter K, et al. Hyperlipidemia disrupts cerebrovascular reflexes and worsens ischemic perfusion defect. 2013;33(6):954-62.

80. Popkin BMJHA. Will China's nutrition transition overwhelm its health care system and slow economic growth? 2008;27(4):1064-76.

81. Song P, Zha M, Yang X, Xu Y, Wang H, Fang Z, et al. Socioeconomic and geographic variations in the prevalence, awareness, treatment and control of dyslipidemia in middle-aged and older Chinese. 2019;282:57-66.

82. Xu S, Ming J, Yang C, Gao B, Wan Y, Xing Y, et al. Urban, semi-urban and rural difference in the prevalence of metabolic syndrome in Shaanxi province, northwestern China: a population-based survey. 2014;14(1):104.

83. Oguejiofor O, Onwukwe C, Odenigbo CJAoAm. Dyslipidemia in Nigeria: prevalence and pattern. 2012;11(4):197.

84. Chen R, Ovbiagele B, Feng WJTAjotms. Diabetes and stroke: epidemiology, pathophysiology, pharmaceuticals and outcomes. 2016;351(4):380-6.

85. Lekoubou A, Clovis N, Dzudie A, Kengne APJPCD. Diagnosed diabetes mellitus and in-hospital stroke mortality in a major sub-Saharan African urban medical unit. 2017;11(1):57-62.

86. Alene M, Assemie MA, Yismaw L, Ketema DBJBn. Magnitude of risk factors and in-hospital mortality of stroke in Ethiopia: a systematic review and meta-analysis. 2020;20(1):1-10.

87. Veerbeek JM, Winters C, van Wegen EE, Kwakkel GJPo. Is the proportional recovery rule applicable to the lower limb after a first-ever ischemic stroke? 2018;13(1):e0189279.

88. Hawe RL, Scott SH, Dukelow SPJS. Taking proportional out of stroke recovery. 2019;50(1):204-11.

89. Krakauer JW, Marshall RSJAon. The proportional recovery rule for stroke revisited. 2015;78(6):845-7.

90. Kjellstrom T NB, Shatchkute A. Helsingborg Declaration 2006 on European stroke strategies. Cerebrovasc Disease. 2007;23:231-41.

91. Ginenus Fekadu LC, Tsegaye Melaku, Busha Gamachu, Mohammed Gebre, Firomsa Bekele, Getahun Fetensad. Management protocols and encountered complications among stroke patients admitted to stroke unit of Jimma university medical center, Southwest Ethiopia: Prospective observational study. Annals of Medicine Surgery. 2019:135–43.

92. Sarfo FS, Akassi J, Awuah D, Adamu S, Nkyi C, Owolabi M, et al. Trends in stroke admission and mortality rates from 1983 to 2013 in central Ghana. 2015;357(1-2):240-5.

Figure Legends

Figure 1: Flow chart to a selection of studies for a systematic review and meta-analysis of the proportion of adherence to healthy lifestyle modification of people with hypertension in Ethiopia 2020.

Figure 2: Frost plot of in the proportion of hemorrhagic stroke in Ethiopia, 2020.

Figure 3 Forest plot of in the proportion of ischemic stroke in Ethiopia, 2020.

Figure 4: Forest plot of the proportion of recover during discharge after stroke in Ethiopia, 2020.

Figure 5: Meta funnels presentations of the proportion of recover after stroke in Ethiopia, 2020, whereby SE PIV (standard error of proportion) plotted on the Y-axis and log PIV (logarithm of proportion.

Figure 6: One-leave-out sensitivity analysis for studies conducted on the pooled estimated proportion of stroke clinical outcome in Ethiopia, 2020.

Click here to access/download

Supporting Information

s1 search technique.docx

Click here to access/download
Supporting Information
001Stroke figure plos.docx

Click here to access/download
Supporting Information
01 Appendix 3 quality assessment NOS.xlsx

Click here to access/download
Supporting Information
01S2 qaulity appraisal risk assessment.docx

Click here to access/download
Supporting Information
S3 sex proportion of stroke in Ethiopia.docx

Click here to access/download
Supporting Information
S4 PRISMA check list.docx

Click here to access/download
Supporting Information
Data extraction tables.xlsx

The burden of ~~Patient recovery from~~ stroke and modifiable risk factors in Ethiopia: a systemic review and meta-analysis.

Teshager Weldegiorgis Abate[1]*, Balew Zeleke[2&], Ashenafi Genanew[3¶], and Bidiru Weldegiorgis Abate[4¶]

[1]Department of adult health Nursing, School of Health Science, College of Medicine and Health Science, Bahir Dar University, P.O. Box 79, Bahir Dar, Ethiopia.

[2]Department of Pediatric and Child Health Nursing, School of Health Sciences, College of Medicine and Health Sciences, Bahir Dar University, P.O. Box 79, Bahir Dar, Ethiopia.

[3]Department of pharmacology, School of Health Sciences, College of Medicine and Health Science, Bahir Dar University, P.O. Box 79, Bahir Dar, Ethiopia.

[4]Department of Anesthetics, Addis Alem Hospitla, Amhara Regional Bureau

*Corresponding author: teshagerhylemarriam@gmail.com, phone number +251912886162

[¶]These authors contributed equally to this work.

[&]This authors also contributed equally to this work

Abstract

Background: The burden and contribution of modifiable risk factors ~~to~~ of stroke ~~the increasing global and regional~~in Ethiopia ~~burden of stroke is~~ are unclear.~~, but~~ ~~k~~Knowledge about this burden and modifying risk factors ~~contribution~~ is pivotal ~~crucial~~ for establishing ~~informing~~ stroke prevention strategies. In recent decades, the issue of modifiable risk factors ~~and stroke recovery is one of the~~ a key~~s~~ to improves~~ing~~ the quality of life. Modify the risk factor is an important issue ~~a strong recommendation~~ to prevent stroke. So far, many primary studies ~~were~~ conducted to estimate the proportion ~~recovery~~ of stroke burden and modifiable risk factors in Ethiopia.~~,~~ However, the lack of a nationwide study that determines the overall pooled estimation burden and ~~proportion of~~ modifiable risk factors ~~and recovery~~ of stroke is a ~~an important~~ research gap.

Methods: To conduct ~~T~~this ~~study~~ review, we are ~~was conducted~~ following the PRISMA checklist. Three reviewers have been searched and extracted from the CINAHL (EBSCO), MEDLINE (via Ovid), PubMed, EMcare, African Journals Online (AJOL), and Google scholar. ~~Articles'~~ The quality of the primary study ~~was~~ assess~~ed~~ using the Newcastle-Ottawa Scale (NOS) by two independent reviewers, and only studies with low and moderate risk ~~were~~ include~~d~~ in the final analysis. The review presented the pooled proportion of stroke recovery and modifiable risk factors of stroke in ~~people~~ stroke patients~~with stroke~~. The ~~review has been~~ registered ~~in PROSPERO with~~ protocol number in PROSPERO is CRD42020221906.

Results: In this study, the pooled proportion stroke recovery in Ethiopia was 72.28% (95%CI: 62.48, 82.08; I2 = 96.5%). The overall magnitude modifiable risk factor of hypertension, alcohol

consumption and dyslipidemia among ~~people~~ stroke patients~~with stroke~~ were 49% (95%CI: 43.59, 54.41), 24.96% (95CI%:15.01, 34.90), and 20.99% (95%CI: 11.10, 30.88), respectively. The least proportion of stroke recovery was in the Oromia region (67.38 (95%CI: 41.60-93.17; I2 = 98.1%). Besides, the proportion of stroke recovery was decreased after 2017 (70.50 (56.80 - 84.20).

Conclusions: ~~Even though our pooled result showed that stroke recovery is in line with the ideal proportional recovery rule of stroke, this not satisfactory in the comparison of other developing countries.~~ In our study, ~~M~~more than 90% of stroke patients had one or more modifiable risk factors. All identified modifiable stroke risk factors are major public health issues in Ethiopia.~~, especially high blood pressure and alcohol consumption, should be understood as a major public health problem in Ethiopia and~~Therefore, eff orts ~~should be~~ focused on the primary prevention of stroke. Such as lower blood pressure, lose weight, exercise more, don't drink alcohol, or do it in moderation, treat atrial fibrillation and diabetes, and quit smoking.

Keywords: Stroke, Modifiable risk factors, Stroke recovery rate, Meta-analysis, Ethiopia

Background

Stroke remains the second leading cause of death worldwide in all sexes, with an annual mortality rate of 5·5 million. ~~Stroke has increased across the globe in both men and women of all ages.~~ Fewer women (2·6 million) than men (2·9 million) died from stroke (1, 2). The incidence, prevalence, and mortality rate of stroke have been increased worldwide, with most of the burden being in the low and middle-income country including Ethiopia (3, 4). ~~Ischemic stroke is more frequent, but h~~Hemorrhagic stroke is responsible for more deaths~~,~~ and Disability-Adjusted Life-Years (DALYs).~~ are lost.~~ Incidence and mortality of stroke differ between countries, geographical regions, and ethnic groups (5).

Ethiopia faces the unenviable threat of a triple burden of disease: infectious ~~or communicable~~ diseases, Non-Communicable Diseases (NCDs), and injuries are steadily increasing and pose risks to the lives of Ethiopians (6). Although Ethiopia is progressing towards ~~universal~~ national health coverage, the country faces the double burden of both communicable and NCD (7). The magnitude of stroke-related deaths in Ethiopia ~~was~~ is 6.23% out of total deaths, and the age-adjusted death rate of stroke in the country is 89.82 per 100 000 of the population (8). Besides, previous reports indicated that 90% of the burdens of stroke are attributable to modifiable risk factors (9). Of this, three-quarters of the stroke burden is attributable to behavioral risk factors (10).

Most of the stroke burden was attributable to modifiable risks. ~~For example,~~These are: metabolic factors (high blood pressure, obesity, fasting plasma glucose, cardiac disorder, and total cholesterol) accounted for 72% of stroke DALYs, and behavioral factors (smoking, poor diet, and physical inactivity) accounted for 66%. ~~, and environmental risks (air pollution and lead exposure) accounted for about 28%. Although most of the risk attribution information is not novel, many of these risks are modifiable. They have been shown to reduce stroke and important to track and emphasis~~ (5, 11-15).

~~Though there were many primary studies conducted to estimate the magnitude of subtypes of stroke,~~In Ethiopia, a comprehensive national representative stroke burden and the modifiable risk factor are lacking. ~~of stroke, and rate of improvement at discharge after stroke in Ethiopia, informative and comprehensive reviews are lacking, and the magnitude of subtypes of stroke, modifiable risk of stroke, and rate of improvement at discharge after stroke is an important research gap. Meta-analysis is key to improve the accuracy of estimates through the use of more data sets.~~ Thus, this study ~~was~~ aimed to determine the overall magnitude ~~subtypes~~ of stroke, modifiable risk factors of stroke, and ~~rate~~ improvement rate after stroke in Ethiopia.

Methods and analysis

Protocol design and registration

A systematic review ~~and~~ with a meta-analysis of published and unpublished observational studies ~~published and unpublished research articles that were~~is incorporated ~~conducted~~ to assess the magnitude of ~~ischemic and hemorrhagic~~ stroke and modifiable risk factors of stroke~~the rate of improvement at discharge~~ in Ethiopia. ~~were included during this study.~~ The Preferred Reporting Items for Systematic Review and Meta-analysis Protocol (PRISMA-P) (16, 17), and Meta-analysis of Observational Studies in Epidemiology (MOOSE) guideline statement (18) ~~were~~ used ~~for~~ to the development of this study protocol, the conduct and design, and the reporting of these results. ~~To minimize duplication of the same review, provide transparency, and to reduce reporting bias of the current review, an intensive search was done before data extraction. A~~ This review protocol register~~ed~~ in the International Registration of Systems Reviews (PROSPERO) (registration number CRD42020221906).

Eligibility Criteria

The eligibility of the study was determined using the following criteria: (1) all facility-based observational studies; (2) all studies conducted in Ethiopia; (3) all studies reporting either the magnitude of any subtypes of stroke or rate of improvement at discharge and modifiable risk factors; and both published and unpublished studies. On the other hand, anonymous reports, case reports, qualitative, and ~~research which were~~ unable to access the full_-text after three email contacts of principal investigators of the particular studies ~~were~~ excluded ~~from~~ the analysis.

Information source and search strategies

We used ~~S~~standardized and well-described methods~~ were used~~ in this systemic review (16). Briefly, a search strategy ~~was~~ design~~developed after the identification of~~ using the relevant Medical Subject Headings (MESH), keywords, and ~~their~~ synonyms. The search strategy for ~~Pumed~~PubMed: the key~~-~~words which we used in our search included terms describing stroke, ~~terms describing~~ age, and ~~term describing~~ modifiable risk factors as shown in the search strategy as follows: (1) (Stroke [Title] OR "Ischemic stroke"[Title] OR "Ischaemic stroke"[Title] OR "Haemorrhagic stroke"[Title] OR "Hemorrhagic stroke" OR "Cerebral Vascular ~~a~~Accident" OR CVA); (2) (Adults OR "18 years or older") [Text Word] (3) (Ethiopia) [Text Word] **(**4**)** (Hypertension OR "High blood pressure" [Text Word] OR Diabetes [Text Word] OR "Diabetes mellitus" OR "Smoking" OR "Obesity" OR Alcohol OR "Heavy drinking" [Text Word] OR Physical exercise OR "Physical activity" [Text Word] OR (High blood cholesterol level OR "Hypercholesterolemia, Hyperlipidemia" OR "Hyperlipoproteinemia" OR "Arterial fibrillation) [Text Word] (5) #1 AND #2 AND #3 AND #4 (S1.).

A pretest of the search strategy by two authors ~~was~~ perform~~ed~~ in PubMed.~~, and t~~The actual electronic search ~~was done~~ did from November 20 to 25, 2020. Two independent reviewers implemented the electronic search in the following electronic databases: CINAHL (EBSCO), MEDLINE (via Ovid), PubMed, EMcare, AJOL, and Google scholar search engines~~. Moreover, snowballing and retrieving references or hand searching was performed on the reference lists of eligible studies to include studies that unable to identify by the search strategy~~. Finally, the search process ~~was~~ present~~ed~~ in a PRISMA flow chart.

Study Selection

Two of the reviewers (TWA and BWA) screened the titles and abstracts of each article to find potentially eligible studies. After removing duplicates, the search results ~~were~~ are exported to

End-Note software (version X7 Thomson Reuters, New York, NY) ~~used~~ to create a bibliographical database of the retrieved references. The selection process ~~was~~ conduct~~ed~~ in two stages: first screening of titles and abstracts against the predetermined inclusion/exclusion criteria, followed by a second screening of the full text of the research reports identified as probably relevant in the initial screening. Both stages were carried out independently by two authors (TWA and AG), and disagreements resolved by discussion with another author (BWA) determined the ~~last~~ eligibility of the included ~~articles~~in the review. ~~Articles that fulfilled the earlier criteria were used as a source of data for analysis.~~

Data Extraction process and Quality Assessment

The abstract and full-text review data abstraction was done by three independent authors (TWA, BZ, and AG) using a pre-piloted data extraction format prepared in the Microsoft™ Excel spreadsheet. Disagreement in data abstraction between the first two and third authors ~~was~~ resolved by a fourth independent author (BWA). From each observational ~~primary~~ study, we have extracted data participant sex, study year, region, sample size, study design, and first author name for the proportion of ischemic stroke, hemorrhagic stroke, improvement at discharge, and each risk factors (hypertension, diabetes mellitus, alcohol consumption, smoking, heart disease, lack physical activities, cholesterol, and obesity).

Before analysis, ~~a~~prevalence transformation ~~of prevalence was made~~carries out. The Newcastle-Ottawa Scale (NOS) was used to select for assessing the quality of the included studies. The NOS ~~included~~ had three categorical criteria with a maximum score of ten points. The assessment tool contains representatives of the sample, sample size, non-respondents, and ascertainment of exposure, independent blind assessment, and statistical test. Based on ~~this tool~~NOS, ~~a primary study with~~ a score of 6 out of 10 ~~was~~ consider~~ed~~ as good quality. ~~In the review~~

~~that t~~To maintain the validity of this review, we only included primary studies with fair to good quality (17-19).

The primary outcome of this review was the pooled proportion of the overall ischemic, hemorrhagic, and survival rate discharge~~,~~ and the magnitude of common modifiable risk factors among ~~people~~ stroke patients ~~with stroke~~ in Ethiopia. Stroke ~~was defined as~~is rapidly developing clinical signs of focal, or at times, global disturbance of cerebral function, lasting more than 24 hours or leading to death with no apparent cause other than a vascular origin (20, 21).

Quality Assessment

The 10-items rating scale uses to assess for each observational study ~~risk of bias of included studies is assessed using the 10-item rating scale developed by Hoy et al. for prevalence studies~~ (22). The assessment tool has a representative sample size, ~~method of~~ data collection method, reliability, and validity of study tools, case definition, and prevalence periods of the studies. Researchers categorized each observational article study as having a low risk of bias ("yes" answers to domain questions) or a high risk of bias ("no" answers to domain questions). Each study assigned a score of 1 (Yes) or 0 (No) for each domain, and these domain scores ~~were~~ sum~~med~~ to give an overall study quality score. Scores of 8–10 ~~were~~ consider~~ed~~ as having a "low risk of bias,"~~;~~ 6–7 a "moderate risk,"~~;~~ and 0–5 a "high risk."~~.~~ For the least risk of bias classification, discrepancies between the reviewers resolved via consensus.

Data analysis

Heterogeneity test and publication bias

Heterogeneity between the findings of the primary studies was assessed by using Cochran's Q test and quantified with the I-square statistics. A P-value of less than 0.1 ~~was~~ considers~~ed as~~ suggesting statistically significant heterogeneity.~~,~~ A heterogeneity considering a small number of

studies and their heterogeneity in design (23). Heterogeneity classifications are: ~~had taken~~ when I-square values below 25% low, 25-75% moderate, and above 75% high ~~classifications when I-square values were below 25%, between 25 and 75%, and above 75% respectively~~ (24). The random-effect model was used to pool the recommended health life rate,~~.~~ ~~The random-effect model~~ accounts for heterogeneity among study results beyond the variation associated with the chance ~~unlike the fixed effect model~~ (25).

We used the random-effect model ~~T~~to investigate the source of heterogeneity.~~,~~ ~~the random-effect model was used.~~ The meta-analysis was weighted to account for the residual between-study heterogeneity (i.e., heterogeneity not explained by the covariate in the regression (26). Publication bias ~~was~~ assesses~~d~~ by visual inspection of funnel plots based on the shape of the graph. The symmetrical graph ~~was~~ interprets~~ed~~ as an absence of publication bias. An asymmetrical one indicated the presence of publication bias.

~~On the other hand,~~ We employed Begg's and Egger's weighted regression ~~was employed~~ to identify the source of publication bias. P-values less than 0.05 were considered as the presence of significant publication bias (27, 28). We also applied~~A~~ leave-out sensitivity analysis ~~performed~~ to confirm some studies potentially biased the direction of the pooled estimate. Subgroup analyses by region and type of study setup (hospitals) ~~were~~ carried out because of significant heterogeneity between studies (i.e., $I^2 = 96.5\%$, $p<0.05$)

Statistical analysis

Data ~~were~~ analyzed in Stata Version 14. Data was presented in the evidence table and summarized using descriptive statistics. The effect measure for outcome variables ~~were~~ computed using the "Metaprop" command for meta-analysis of the proportion in Stata. In this review, the overall burden of ~~ischemic stroke, hemorrhagic~~ stroke, rate of improvement, and common

modifiable risk factors ~~was~~ calculate~~d~~ together with their corresponding 95% CI. A forest plot ~~was~~ generates~~d~~ to show the individual pooled burden of strokes and common modifiable stroke 95% CI, the author's name, study year, and study weights.

Result

Study selection process

From electronic databases, we retrieved 986 observational studies. After screening their titles and abstracts, duplications ~~were~~ removed using Endnote X7. Accordingly, we removed 644 duplicate articles~~ were removed~~. ~~From~~ Of 342 articles, 315 articles ~~were~~ excluded~~d~~ because their titles and abstracts were not in ~~-~~line with our inclusion criteria (full article not found, different population, different setting, and different outcome). Finally, 27 articles ~~were~~ include~~d~~ for this systemic review and meta-analysis (Fig. 1).

Study characteristics

Overall, we selected a total of 27 observational studies ~~were selected~~ in this systematic review and meta-analysis. ~~This~~ We included a total ~~consisted~~ of 5,845 participants. Among them, 2,647 participants were male, and 3,228 participants ~~were~~ improved at the time of discharge. The number of participants in each study ranged from 73 to 503. The most retrieved studies (n = 8) were from the Oromia (29-36) region followed by Addis Ababa (n=7) (37-43) and Amhara region (n=7) (44-50), Tigray region represented by four studies (51-54), ~~whereas~~ Southern Nations Nationalities and People's (SNNP) (55) region ~~represented~~ had ~~by~~ one study.

The smallest sample size was 73 obtained from a study conducted at Shashemene Referral Hospital, Ethiopia (29).~~ Whereas, t~~ The largest sample size was 503 reported from a study done at Ayder Comprehensive Specialized Hospital, Northern Ethiopia (52). Most studies dealt with

hypertension as a modifiable risk factors of stroke (n=24) (29-34, 36, 37, 39-41, 43, 44, 48, 51-59) followed by Arterial Fibrillation (AF) (n = 17) (30-32, 37, 40, 41, 44, 45, 47-50, 53-55, 57, 58), Diabetes mellitus (DM) (n = 15) (30, 31, 33, 37, 39-41, 44, 48, 49, 55-59), other heart disease rather than AF (n = 14) (29, 30, 32, 37, 41, 43, 47, 49, 54-59), and high cholesterol level (n = 7) (32, 33, 35, 37, 44, 48, 49, 51, 55) (Table1).

Table 1: Study characteristics of included articles for the final systematic review and meta-analysis on the burden of modifiable risk factors and rate of improvement at discharge after stroke in Ethiopia 2020

Authors name	Study year	Region	Sample size	Study design	Burden of Stroke reported outcome percentage (95% CI)					
					Ischemic stroke	Hemorrhagic stroke	Female	Male	Improvement at discharge	NOS score
Asgedome SW.et al	2019	Tigray	216	R	55.6	44.4	58.3	41.7	77.8	8
Asres AK. et al	2018	AA	170	CC	51.2	37.6	42.9	57.1	72.4	7
Baye M. et al	2018	Amhara	448	R	31.5	68.5	58.0	42.0	59.8	8
Bedassa T. et al	2018	Oromia	242	R	64.3	35.7	no	Not	Not	5
Beyene DT. et al	2017	Oromia	367	R	35.7	64.31	36.2	63.8	26.4	8
Dandena A. et al	2019	Oromia	283	P	43.1	44.5	35.0	65.0	Not	6
Deresse B. et al	2014	SNNP	163	P	50.3	49.7	33.7	66.3	85.3	8
Erkabu SG. et al	2016	Amhara	303	R	59.4	40.6	37	63.0	89.0	7
Fekadu G.et al	2017	Oromia	116	P	51.7	48.3	37.1	62.9	Not	6
Fekadu G.et al	2017	Oromia	116	CC	48.3	41.6	37.1	62.9	78.4	8
Fekadu G.et al	2017	Oromia	364	CC	42.3	57.7	42.9	57.7	94.0	7
Gebremariam SA. et al	2014	Tigray	142	CC	55.6	38.0	45.8	54.2	47.9	8
Gebreyohannes EA.et al	2017	Amhara	208	R	57.7	Not	57.7	42.3	87.5	7
Gedefa B. et al	2016	AA	163	R	35.6	64.4	43.6	56.4	69.9	8
Gelan Y. et al	2016	AA	227	CC	49.8	48.9	30.0	70.0	70.0	7
Greffie. ES et al	2013	Amhara	98	R	69.4	30.6	53.1	46.9	87.0	7

Gufue ZH. et al	2019	Tigray	503	R	56.6	43.4	50.1	49.9	85.1	7
Kassaw A.et al	2018	AA	170	R	51.2	48.8	42.9	57.1	80.0	8
Kefale B. et al	2019	Oromia	111	R	80.1	18.0	50.5	49.5	83.8	7
Mekonen HH.et al	2018	Tigray	89	R	32.6	36.6	63.2	51.7	Not	5
Mulat B. et al	2015	Amhara	427	R	56.7	43.3	63.2	36.8	Not	6
Mulugeta H. et al	2019	Amhara	162	R	50.0	30.0	53.7	46.3	27.2	7
Sultan M. et al	2014	AA	301	p	53.8	17.9	42.5	57.5	80.7	8
Tamirat KS. et al	2017	Amhara	151	R	60.3	39.7	50.3	49.7	90.7	7
Temesgen TG.et al	2017	Oromia	73	R	65.8	34.2	42.5	57.5	54.8	6
Zenebe G. et al	2001	AA	128	CC	43	57.0	39.8	61.7	Not	6
Zewdie A. et al	2016	AA	104	CC	44.2	55.8	44.0	56.0	Not	5

R: Retrospective, P: Prospective, CC: Cross-Sectional, AA: Addis Ababa, NOS: Newcastle-Ottawa Scale

Quality Appraisal

The quality score of the included study ranged from 5 to 8 to a mean score of 7.04 (SD = 0.94). Out of 27 studies, 21 (77.78 %) studies received a low risk of bias. ~~The authors also find types of bias: 5~~Five studies (29-31, 36, 39, 44, 49, 51, 55) had a high risk of case definition, ~~5 ~~five studies (29, 30, 35, 55, 56) had ~~a high risk of ~~random selection bias, and 14 studies (29, 33-35, 39, 41, 47, 51, 53, 57, 58) had a high risk of representation bias (S2.).

The ~~M~~magnitude of strokes in Ethiopia

From the total rank~~ed~~ of twenty-~~-~~seven primary studies, twenty-~~-~~five, twenty-~~-~~six studies provided information on the proportion of hemorrhagic and ischemic stroke~~ respectively~~. Twenty-six ~~primary ~~studies also provide information on stroke ~~the ~~proportion ~~of stroke on ~~in females and males. ~~Whereas t~~Twenty primary studies reported the rate of improvement at discharge after stroke. As presented in the forest plot (Fig. 2 and 3), the pooled estimate proportion of hemorrhagic and ischemic stroke were 46.42% (95%CI: 41.82-51.53; $I^2 = 91.6\%$) and 51.40% (95%CI: 46.97-

55.82; I^2 = 85.5%) respectively. ~~High heterogeneity was observed among the included studies.~~ The pooled estimate of stroke among female was 45.07 % (95%CI: 41.80-48.35; I^2 = 80.3%) and male was 54.70 % (95%CI: 51.32-58.08; I^2 = 79.5%) (S3.).

The ~~M~~magnitude of modifiable risk factors of stroke in Ethiopia

We investigated the magnitude of modifiable risk factors of stroke among the included studies. The proportion of DM among stroke patients ranged from 5.2% (54) to 21.6% (39). A total of 3~~,~~356 stroke ~~people~~ patients ~~were~~ include~~d~~ ~~to~~ determin~~e~~inge the pooled magnitude of DM among stroke ~~people~~patients. Accordingly, our pooled analysis showed that 14.722% (95%CI: 9.51, 19.94; I^2 = 95.8) of stroke ~~people~~ patients had DM. In this review, stroke ~~people~~ patients who had hypertension ranged from 24.1% (33) to 75.2% (32).

We used a total of 5064 stroke patients ~~T~~to determine the pooled magnitude of hypertension risk factors ~~among~~ in stroke patients~~, a total of 5,064 people with stroke were included~~. Consequently, we found that the overall ~~magnitude~~ pooled estimation of hypertension among stroke ~~people~~ patients was 49% (95CI%:43.59, 54.41; I^2 = 91.6%). Furthermore, the proportion of ~~prestrike story excessive~~ alcohol consumption (more than two drinks in a day for men and more than one drink in a day for women) among stroke ~~people~~ patients included in this study ranged from 10.4% (55) to 41.4% (48). ~~, and o~~Our meta-analysis revealed that 24.96 % (95%CI: 15.01, 34.90; I^2 = 92.7) of ~~people with~~ stroke patients had a history of ~~excessive~~ harmful alcohol intake (Table 2).

Table 2: The pooled effect of common modifiable risk factors among the primary studies of stroke in Ethiopia.

Modifiable risk factors	Estimated pooled proportion (95%CI)	I-squared (%)
Hypertension	49 (43.59, 54.41)	91.6

Diabetes mellitus	14.72 (9.51, 19.94)	95.8
Atrial fibrillation	19.21 (13.96, 24.46)	94.4
Other heart disease	20.11 (14.27, 25.95)	94.2
Dyslipidemia	20.99 (11.10, 30.88)	96.4
Smoking	10.38 (6.27, 14.94)	86.0
Obesity	11.64 (2.48, 20.79)	95.3
Alcohol	24.96 (15.01, 34.90)	92.7

Other heart Disease: Congestive heart failure, Structural heart disease, Myocardia friction

Recovery from stroke in Ethiopia

The ~~P~~proportion of improvement during discharge after stroke among the included primary studies was ranged from 26.4% (30) to 94% (36). We used ~~A total of~~ 2321 stroke ~~people~~ patients ~~were included~~ to ~~determine~~ estimate the pooled ~~magnitude~~ proportion of improvement at the time of discharge. ~~among stroke patients, and t~~The pooled improvement status of stroke during discharge in Ethiopia was 72.28% (95%CI: 62.48, 82.08; $I^2 = 96.5\%$) (Fig. 4).

Publication bias

Both funnel~~s~~ plots of precision asymmetry and Egger's intercept test ~~of the intercept~~ showed ~~that there is~~ no publication bias in the primary studies. Visual examination of the funnel plot showed ~~a~~ the symmetric distribution ~~of studies~~. Additionally, Egger's intercept test o~~f the intercept~~ was -0.147 (95% CI: -0.26, 1.18) p > 0.05 (0.102), as judged by Egger's test, there was no evidence of publication bias ~~being~~ present~~ed~~ at a 5% significance level (Fig. 5).

Subgroup analysis

Due to the heterogeneity of included studies, ~~further~~ we performed a sub-group analysis ~~was done by~~ using the following study characteristics: region, sample size, and study year. We applied

~~T~~the random-effect model ~~was applied~~ for reporting the pooled proportion of clinical outcomes during discharge of the sub~~-~~group analysis. Accordingly, the highest recovery rate ~~was~~ observed from the Addis Ababa region 74.51 (69.84-79.17; I^2 = 34.5%). ~~Whereas t~~The least pooled proportion of recovery was in the Oromia region (67.38 (95%CI: 41.60-93.17; I^2 = 98.1%). The subgroup analysis by study year showed that the pooled proportion of recovery rate after stroke during discharge was 75.59 % (95%CI 64.28 - 86.9; I^2 = 92.1%) for studies conducted before 2017 (Table 3).

Table 3: Subgroup analysis of recovery after stroke by region, sample size, and study year in Ethiopia 2020.

Variables	Characteristics	Estimated stroke recover during discharge (95% CI; I^2 = %)
Region	Oromia	67.38 (41.60-93.17; I^2 = 98.1)
	Addis Ababa	74.51 (69.84-79.17; I^2 = 34.5)
	Amhara	73.44 (50.29 – 96. 59; I^2 = 97.9)
	Tigray	70.19 (47.10 - 93.28; I^2 = 95.7)
	SNNPR	Single study
Sample size	<223 (median)	72.41 (60.00 - 83.82; I^2 = 96.0)
	>=223 (median)	72.05 (527.44 - 91.66; I^2 = 97.5)
Study year	Before 2017	75.59 (64.28 - 86.9; I^2 = 92.1)
	After 2017	70.50 (56.80 - 84.20; I^2 = 92.1)

SNNPR: South Nations, Nationalities and People Region

Meta-regression and sensitivity analysis

The sub~~-~~group analysis showed that heterogeneity across the studies was widespread. To name the source of heterogeneity, we conducted a meta-regression and sensitivity analysis. During the meta-regression analysis, we ~~conducted~~ applied using the following study covariance: study years and region. However, the results showed that none of these variables were a statistically significant source of heterogeneity. We also performed a sensitivity analysis to find the influence of each study on the overall effect size. No single study ~~much~~ affected the overall pooled proportion of clinical outcomes of ~~stroke among~~stroke among stroke ~~patients in~~patients in Ethiopia (Table 4, Fig. 6).

Table 4: Meta-regression output to explore the heterogeneity of the pooled proportion of clinical outcome of stroke in Ethiopia, 2020.

Variables	Coefficients	P-value	95% CI
Study Year	-4.83	0.538	-21.03, 11.35
Region			
Addis Ababa	-10.68	0.660	-61.37, 40.01
Amhara	-11.67	0.626	-61.63, 38.29
Oromia	-17.72	0.468	-68.44 33.01
Tigray	-14.83	0.563	-68.26 38.61

Discussion

This study ~~was~~ aimed to determine the overall proportion of ~~patient~~ stroke burden~~recovery after stroke,~~ and modifiable risk factors in Ethiopia. ~~Accordingly, from~~ Of all stroke cases in our review, more than half (51.40%) of stroke patients in Ethiopia was an ischemic stroke. This finding was almost similar to a study done in Kenya (56.1%) (60), much lower than a study done in China

(81.9- 91.7%) (61, 62), Burkina Faso (61.63%) (63), Iran (76.5 - 81.9%) (64, 65), and a 22 country case-control study (78%) (13). The difference in culture and economic status, lifestyle difference, poor management of modifiable risk factors, and difference in the preventive strategies in the general public could be the reasons for the difference.

For all stroke cases, ~~men~~ males stroke patients (54.70%) are relatively higher prevalence than ~~women~~ females stroke patients (45.07%) ~~with~~ by a 54.70: 45.07 split of male and female in all recruited patients in Ethiopia. A systematic review ~~in~~ of epidemiological studies on Western European surveys~~,~~ ~~have~~ has shown that stroke is more common in ~~men~~ males than in ~~women~~ females (66). The gender difference might be ~~men~~ males present a higher risk of stroke than ~~women~~females. But ~~women~~ females suffer ~~of~~ from a stroke ~~in~~ at older ages making them more prone to die from stroke than are ~~men~~males. Females were risk factor avoidance than ~~men~~ males. ~~s~~Such as females are less likely to drink heavily or smoke than males (67).

Our meta-analysis showed that almost half (49%) of all stroke patients had hypertension. Previous evidence also showed that 75.8 % of ~~people with~~ stroke patients had hypertension (63), ~~people with~~ hypertensive~~on~~ individuals are two to four times more likely to have a stroke (13, 61, 68). ~~This is because h~~Hypertension has remained the leading modifiable ~~predictor~~ risk factors of stroke morbidity and mortality since 1990 (69). People who can ~~M~~maintain~~ing a~~ normal blood pressure ~~and lowering high blood pressure~~ can decrease the risk of stroke by 30 to 40% (70).

Though hypertension is the main reported modifiable risk factor of stroke among the included primary studies, the pooled proportion of hypertension among ~~people~~ stroke patients~~with stroke~~ found in the current study is lower than the previous studies conducted in Burkina Faso (63), Iran (65), China (62), Bosnia-Herzegovina (71), Nigeria (72), and Bangladesh (73). The possible

explanation for this variation might be due to the lack of diagnostic modalities and proficiency, level of income, hypertension awareness, treatment, and control (74).

~~Excessive~~ Above limit, alcohol consumption is a well-established risk factor of stroke.~~, and~~ In our ~~analysis showed that~~review, alcohol consumption is the second most common modifiable risk factor~~s~~ of stroke. Almost one-fourth (24.96%) of ~~people with~~ stroke patients had a history ~~excessive~~ of alcohol consumption. ~~This is b~~Because drinking ~~excessive~~ the upper limit~~amounts~~ of alcohol intake can trigger atrial fibrillation – a type of irregular heartbeat. Atrial fibrillation increases the risk of stroke by five times~~,~~ because it can cause blood clots to form in the heart. If these clots move up into the brain, it can lead to stroke (75).
A review of 84 studies of alcohol consumption and cardio~~-~~vascular disease, alcohol consumption >60 g/day increased the risk of incident stroke by 62% compared with abstaining from alcohol (76). ~~This~~ The pooled proportion of alcohol consumption among ~~people with~~ stroke patients in Ethiopia is higher than a study conducted in Nigeria (72). The possible explanation for this variation might be ~~due to~~ the lack of diagnostic modalities and proficiency; measured dyslipidemia in the medical record before the occurrence of stroke. Another reason for this variation, ~~Even though Ethiopia passes tough law regulating alcohol sales and adverts, currently,~~ there is no effective community action to control alcohol consumption in Ethiopia (77, 78).

In our current study, dyslipidemia is ~~a~~ the third most common modifiable risk factor~~s~~ of stroke. More than two-tenth (20.99%) of~~ people with~~ stroke patients had dyslipidemia. ~~This is b~~Because of this, dyslipidemia promotes cervical or coronary atherosclerosis, which predisposes to athero-thrombotic and cardio-embolic stroke (79). ~~This result~~Our review is comparable with a previous study conducted in Nigeria (72). However, this estimated proportion of dyslipidemia

among ~~people with~~ stroke patients is lower than a study conducted in Bosnia-Herzegovina (71), China (62).

The reasons for the above results could ~~be~~ attribute~~d~~ to the following: first, the dramatic shift of increases in the prevalence of many known risk factors for chronic diseases such as unhealthy lifestyles (~~e.g.~~ decreased physical activity, ~~excessive~~ smoking, ~~excessive~~ alcohol -consumption~~use~~, and familiarize westernized diet) (80, 81). Second, ~~is~~ the impact of ~~China's~~ rapid ~~economic growth and~~ urbanization (increased risk of obesity) ~~in a subsequently more socio-economically developed environment)~~ (82). In Nigeria, ~~a study postulated that high level of~~ dyslipidemia in stroke patients is closely linked to ~~rapid urbanization and~~ a western diet ~~with most urban cities~~and saturated with fast food outlets and ~~increasing sedentary lifestyle which contrasts with our previous highly active agrarian based lifestyle~~physically inactive lifestyle behaviors (83).

Diabetes is a well-established risk factor ~~of~~ for stroke, and our analysis showed that diabetes mellitus is the fourth most common comorbidity of stroke. More than one-tenth (14.72%) of stroke patients had diabetes mellitus. ~~This is b~~Because of this, diabetes causes various micro-vascular and macro-vascular changes ~~often~~ ending in major clinical complications (84). This finding is comparable with the previous study conducted in Nigeria (72); higher than the study done in Burkina Faso (63), sub-Saharan African (85), and ~~a previous~~ a systematic review and meta-analysis done in Ethiopia (86). However, this proportion of diabetes mellitus among ~~people with~~ stroke patients is -lower than a study done in Bosnia-Herzegovina (71) and Iran (65).

~~Recovery after stroke was the main outcome of interest, and t~~The result found in this study showed that the overall pooled estimated proportion of recovery after stroke in Ethiopia was 72.28% (95%CI: 62.48, 82.08). This finding is in line with the ideal proportional recovery rule of stroke (87-89)~~,~~ but still lower than the goal set for 2015 (85%) in the Helsingborg Declaration

2006 (90). Even though recovery after stroke is in line -with the ideal recovery rule of stroke, this result is still lower than the previous study conducted in Bosnia-Herzegovina (71), Iran (65), sub-Saharan Africa (85), and Kenya (60). The possible reason might be the management protocol for stroke patients was sub-optimal and lagging behind the recommended guidelines due to a lack of skilled personnel, appropriate treatment, and diagnostic agents in Ethiopia (91). However, this result is relatively higher than previous studies conducted in Ghana (92).

The subgroup analyses by year of studies showed that the overall pooled proportion of recovery rate after stroke was higher among studies conducted before 2017. The lowest pooled proportion of recovery rate of stroke in this study population after 20717 may reflect the increased exposure to risk factors for stroke due to ongoing epidemiological and demographic transitions.

Limitations

There is considerable heterogeneity across the included studies. The observed heterogeneity may ~~be~~ describe~~d~~ by differences in the study design, the quality of the studies, and sensitivity. Since ~~O~~our study ~~is mainly~~ focused ~~based~~ on ~~the~~ in-patient~~ cases~~, ~~and~~ it cannot ~~be~~ externally validate~~d~~ to the general population.

Implication

This study has many implications for clinical practice and future research. First~~ly~~, the health care provider (~~especially~~ physicians and nurses) can develop effective strategies to improve recovery after stroke and all modifiable stroke~~, especially~~ -like high blood pressure, alcohol consumption behaviors that ~~people with~~ stroke patients exhibit such modifiable stroke risk factors. Second~~ly~~, identify~~ing and understanding that~~ the restriction to modify these modifiable stroke risk factors is the first step in developing evidence-based interventions to promote short and long-term health outcomes and quality of life. Future research should focus on developing and testing a conceptual model that can use ~~to intervention target~~ accessibility

to screening, treatment, sociocultural aspects of stroke risk factor modification in a national context. ~~should considered.~~ Finally, to give a long-term reduction in stroke risk factor~~-~~-related co-morbidity, researchers should assess ways to extend and sustain ~~modify~~lifestyle -modifiable ~~a~~ stroke risk factors and recovery rate after a stroke of this population.

Conclusion

~~Even though our pooled result showed that stroke recovery is in line with the ideal proportional recovery rule of stroke, this not satisfactory in the comparison of other developing countries.~~ There is a high burden of stroke with ~~in Ethiopia is related with~~ a high rate of modifiable risk factors in Ethiopia. More than 90% of patients had one or more modifiable risk factors. Therefore, efforts focused on the primary prevention of stroke. Such as~~All modifiable stroke risk factors, especially~~ lower high blood pressure, ~~and~~ alcohol limitation or moderation, early screening and treatment of atrial fibrillation and diabetes, lose weight, exercise more, and quit smoking.~~consumption, should be understood as a major public health problem in Ethiopia and eff orts should be focused on the primary prevention of stroke.~~

Declarations

Abbreviations

DALYs: Disability-Adjusted Life-Years; NCDs: Non-Communicable Diseases; MESH: Medical Subject Headings; SNNPR: South Nations, Nationalities and People Region; NOS: Newcastle-Ottawa Scale

Ethics approval and consent to participate

Not applicable.

Consent for publication

Not applicable.

Acknowledgments
Not applicable

Reference

1. Gorelick PBJTLN. The global burden of stroke: persistent and disabling. 2019;18(5):417-8.

2. Johnson CO, Nguyen M, Roth GA, Nichols E, Alam T, Abate D, et al. Global, regional, and national burden of stroke, 1990–2016: a systematic analysis for the Global Burden of Disease Study 2016. 2019;18(5):439-58.

3. Yan LL, Li C, Chen J, Miranda JJ, Luo R, Bettger J, et al. Prevention, management, and rehabilitation of stroke in low-and middle-income countries. J Eneurologicalsci. 2016;2:21-30.

4. Mukherjee D, Patil CG. Epidemiology and the global burden of stroke. World neurosurgery. 2011;76(6):S85-S90.

5. Katan M, Luft A, editors. Global burden of stroke. Seminars in neurology; 2018: Georg Thieme Verlag.

6. Kaba MJEJoHD. Non-communicable diseases: unwelcome in Ethiopia. 2018;32(3).

7. Misganaw A, Haregu TN, Deribe K, Tessema GA, Deribew A, Melaku YA, et al. National mortality burden due to communicable, non-communicable, and other diseases in Ethiopia, 1990–2015: findings from the Global Burden of Disease Study 2015. Population health metrics. 2017;15(1):29.

8. BeLue R, Okoror TA, Iwelunmor J, Taylor KD, Degboe AN, Agyemang C, et al. An overview of cardiovascular risk factor burden in sub-Saharan African countries: a socio-cultural perspective. 2009;5(1):10.

9. Feigin VL, Roth GA, Naghavi M, Parmar P, Krishnamurthi R, Chugh S, et al. Global burden of stroke and risk factors in 188 countries, during 1990–2013: a systematic analysis for the Global Burden of Disease Study 2013. 2016;15(9):913-24.

10. Jones P, Jones DJNT. Primary and Secondary Stroke Prevention Strategies. 2017;113(12):42-6.

11. Johnston SC, Mendis S, Mathers CDJTLN. Global variation in stroke burden and mortality: estimates from monitoring, surveillance, and modelling. 2009;8(4):345-54.

12. Mayosi BM, Lawn JE, Van Niekerk A, Bradshaw D, Karim SSA, Coovadia HM, et al. Health in South Africa: changes and challenges since 2009. 2012;380(9858):2029-43.

13. O'donnell MJ, Xavier D, Liu L, Zhang H, Chin SL, Rao-Melacini P, et al. Risk factors for ischaemic and intracerebral haemorrhagic stroke in 22 countries (the INTERSTROKE study): a case-control study. 2010;376(9735):112-23.

14. Gebremariam LW, Chiang C, Yatsuya H, Hilawe EH, Kahsay AB, Godefay H, et al. Non-communicable disease risk factor profile among public employees in a regional city in northern Ethiopia. 2018;8(1):1-11.

15. Shiferaw F, Letebo M, Misganaw A, Feleke Y, Gelibo T, Getachew T, et al. Non-communicable Diseases in Ethiopia: Disease burden, gaps in health care delivery and strategic directions. 2018;32(3).

16. Moher D, Liberati A, Tetzlaff J, Altman DG, med PGJP. Preferred reporting items for systematic reviews and meta-analyses: the PRISMA statement. 2009;6(7):e1000097.

17. Liberati A, Altman DG, Tetzlaff J, Mulrow C, Gøtzsche PC, Ioannidis JP, et al. The PRISMA statement for reporting systematic reviews and meta-analyses of studies that evaluate health care interventions: explanation and elaboration. Journal of clinical epidemiology. 2009;62(10):e1-e34.

18. Modesti PA, Reboldi G, Cappuccio FP, Agyemang C, Remuzzi G, Rapi S, et al. Panethnic differences in blood pressure in Europe: a systematic review and meta-analysis. PloS one. 2016;11(1):e0147601.

19. . Newcastle-Ottawa Scale customized for cross-sectional studies In. available from https://static-content.springer.com/esm/.../12889_2012_5111_MOESM3_ESM.doc.

20. WHO. WHO STEPS stroke manual: the WHO STEPwise approach to stroke surveillance: World Health Organization; 2005.

21. Sacco RL, Kasner SE, Broderick JP, Caplan LR, Connors J, Culebras A, et al. An updated definition of stroke for the 21st century: a statement for healthcare professionals from the American Heart Association/American Stroke Association. 2013;44(7):2064-89.

22. Hoy D, Brooks P, Woolf A, Blyth F, March L, Bain C, et al. Assessing risk of bias in prevalence studies: modification of an existing tool and evidence of interrater agreement. Journal of clinical epidemiology. 2012;65(9):934-9.

23. Higgins JP, Thompson SG, Deeks JJ, Altman DG. Measuring inconsistency in meta-analyses. Bmj. 2003;327(7414):557-60.

24. Cumpston M, Li T, Page MJ, Chandler J, Welch VA, Higgins JP, et al. Updated guidance for trusted systematic reviews: a new edition of the Cochrane Handbook for Systematic Reviews of Interventions. Cochrane Database Syst Rev. 2019;10:ED000142.

25. Borenstein M, Hedges LV, Higgins JP, Rothstein HR. A basic introduction to fixed- effect and random- effects models for meta- analysis. Research synthesis methods. 2010;1(2):97-111.

26. Thompson SG, Higgins JP. How should meta- regression analyses be undertaken and interpreted? Statistics in medicine. 2002;21(11):1559-73.

27. Duval S, Tweedie R. A nonparametric "trim and fill" method of accounting for publication bias in meta-analysis. Journal of the american statistical association. 2000;95(449):89-98.

28. Egger M, Smith GD, Schneider M, Minder C. Bias in meta-analysis detected by a simple, graphical test. Bmj. 1997;315(7109):629-34.

29. Temesgen TG, Teshome B, Njogu PJSr, treatment. Treatment outcomes and associated factors among hospitalized stroke patients at Shashemene Referral Hospital, Ethiopia. 2018;2018.

30. Beyene D, Asefa H. A two year retrospective cross-sectional study on prevalence, associated factors and treatment outcome among patients admitted to medical ward (stroke unit) at Jimma University Medical Center, Jimma, South West, Ethiopia, 2018. J Palliat Med Care. 2018;5(4):1-6.

31. Kefale B, Betero G, Temesgen G, Degu A. Management practice, and treatment outcome and its associated factors among hospitalized stroke patient at Ambo University Referral Hospital, Ethiopia: an Institutional Based Cross Sectional Study. 2019.

32. Fekadu G, Chelkeba L, Kebede AJBn. Risk factors, clinical presentations and predictors of stroke among adult patients admitted to stroke unit of Jimma university medical center, south west Ethiopia: prospective observational study. 2019;19(1):1-11.

33. Bedassa TSJIR. Assessment of the types and factors associated with stroke among adult patients admitted in Adama Hospital Medical College, Ethiopia. 2019;6:S490.

34. Dandena A, Sinaga M, Yirga Y, Zelalem T. CT Scan Pattern of Stroke Patients at Jimma University Medical Center, South West Ethiopia. J Biomedical Journal of Scientific Technical Research. 2020;29(4):22652-7.

35. Fekadu G, Chelkeba L, Kebede AJBn. Burden, clinical outcomes and predictors of time to in hospital mortality among adult patients admitted to stroke unit of Jimma university medical center: a prospective cohort study. 2019;19(1):213.

36. Fekadu G, Adola B, Mosisa G, Shibiru T, Chelkeba L. Clinical characteristics and treatment outcomes among stroke patients hospitalized to Nekemte referral hospital, western Ethiopia. Journal of Clinical Neuroscience. 2020;71:170-6.

37. Sultan M, Debebe F, Azazh A, Hassen GWJEJoHD. Epidemiology of stroke patients in Tikur Anbessa Specialized Hospital: Emphasizing clinical characteristics of hemorrhagic stroke patients. Ethiop J Health Dev. 2017;31(1):13-7.

38. Gedefa B MT, Berhe T, Abera H. Assessment of risk factors and treatment outcome of stroke admissions at St. Paul's teaching hospital, addis ababa, Ethiopia. Neurophysiology. 2017;8:1-6.

39. Zewdie A DF, Kebede S, Azazh A, Laytin A, Pashmforoosh G. et al. Prospective assessment of patients with stroke in Tikur Anbessa specialised hospital, Addis Ababa, Ethiopia. African Journal of Emergency Medicine. 2018;8(1):21-4.

40. Asres AK CA, Bedada T, Gebrekidan H. Frequency, nursing managements and stroke patients' outcomes among patients admitted to Tikur Anbessa specialized hospital, Addis Ababa, Ethiopia a retrospective, institution based cross-sectional study. International Journal of Africa Nursing Sciences. 2020: Jan 1;13:100228.

41. Ayehu Kassaw AC, Tadesse Bedada. Prevalence, nursing managements and patients' outcomes among stroke patients admitted to Tikur Anbessa specialized hospital, Addis Ababa, Ethiopia, 2018: Addis Ababa University; 2018.

42. Zenebe G, Alemayehu M, Asmera JJEmj. Characteristics and outcomes of stroke at Tikur Anbessa Teaching Hospital, Ethiopia. 2005;43(4):251-9.

43. Gelan Y, Weldeab A. Predictors of Stroke Mortality among Patients Admitted to a Hospital in Ethiopia (2070). AAN Enterprises; 2020.

44. Erkabu SG AY, Mihretu DD, Semere A, Alemu YM. Ischemic and hemorrhagic stroke in Bahir Dar, Ethiopia: a retrospective hospital-based study. Journal of Stroke and Cerebrovascular Diseases. 2018;27(6):1533-8.

45. Gebreyohannes EA BA, Abebe TB, Seid MA, Haile KT. In-hospital mortality among ischemic stroke patients in Gondar University Hospital: a retrospective cohort study. Stroke research and treatment. 2019;2019.

46. Greffie ES MT, Getahun S. Risk factors, clinical pattern and outcome of stroke in a referral hospital, Northwest Ethiopia. Clin Med Res. 2015;4(6):182-8.

47. Baye M HA, Gordon-Murer C, Mariscal T, Belay GJ, Gebremariam AA et al. Stroke Characteristics and Outcomes of Adult Patients in Northwest Ethiopia. Frontiers in Neurology. 2020;11:428.

48. Mulugeta H, Yehuala A, Haile D, Mekonnen N, Dessie G, Kassa GM, et al. Magnitude, risk factors and outcomes of stroke at Debre Markos Referral Hospital, Northwest Ethiopia: a retrospective observational study. The Egyptian Journal of Neurology, Psychiatry and Neurosurgery. 2020;56:1-9.

49. Mulat B MJ, Yeseni M, Alamirew M, Dermello M, Asemahagn MA. Magnitude of stroke and associated factors among patients who attended the medical ward of Felege Hiwot Referral Hospital, Bahir Dar town, Northwest Ethiopia Ethiopian Journal of Health Development. 2016;30(3):129-34.

50. Tamirat KS AS, Gebi NB, Gerffie ES. Clinical profile, in-hospital outcome and associated factors of stroke after the start of a standard organized stroke care unit at university of Gondar hospital, northwest Ethiopia. 2019. p. 15.

51. Mekonen HH, Birhanu MM, Mossie TB, Gebreslassie HT. Factors associated with stroke among adult patients with hypertension in Ayder Comprehensive Specialized Hospital, Tigray, Ethiopia, 2018: A case-control study. J PloS one. 2020;15(2):e0228650.

52. Gufue ZH GN, Ayele W, Yifru YM, Hailu NA, Welesemayat ET et al Survival of Stroke Patients According to Hypertension Status in Northern Ethiopia: Seven Years Retrospective Cohort Study. Vascular Health and Risk Management 2020;16:389.

53. Gebremariam SA, Yang HS. Types, risk profiles, and outcomes of stroke patients in a tertiary teaching hospital in northern Ethiopia. J Eneurologicalsci. 2016;3:41-7.

54. Asgedom SW, Gidey K, Gidey K, Niriayo YL, Desta DM, Atey TM. Medical complications and mortality of hospitalized stroke patients. J Journal of Stroke Cerebrovascular Diseases. 2020;29(8):104990.

55. Deresse B, Shaweno DJJotns. Epidemiology and in-hospital outcome of stroke in South Ethiopia. 2015;355(1-2):138-42.

56. Gebreyohannes EA, Bhagavathula AS, Abebe TB, Seid MA, Haile KTJSr, treatment. In-hospital mortality among ischemic stroke patients in Gondar University Hospital: a retrospective cohort study. 2019;2019.

57. Gedefa B, Menna T, Berhe T, Abera H. Assessment of risk factors and treatment outcome of stroke admissions at St. Paul's teaching hospital, addis ababa, Ethiopia. Journal of Neurology Neurophysiology. 2017;8(3):1-6.

58. Greffie ES, Mitiku T, Getahun S. Risk factors, clinical pattern and outcome of stroke in a referral hospital, Northwest Ethiopia. J Clin Med Res. 2015;4(6):182-8.

59. Tamirat K, Abdella S, Gebi N, Gerffie E. Clinical profile, in-hospital outcome and associated factors of stroke after the start of a standard organized stroke care unit at university of Gondar hospital, northwest Ethiopia. 2019.

60. Kaduka L, Muniu E, Oduor C, Mbui J, Gakunga R, Kwasa J, et al. Stroke mortality in Kenya's public tertiary hospitals: a prospective facility-based study. 2018;8(2):70-9.

61. Yi X, Luo H, Zhou J, Yu M, Chen X, Tan L, et al. Prevalence of stroke and stroke related risk factors: a population based cross sectional survey in southwestern China. 2020;20(1):5.

62. Zhang F-L, Guo Z-N, Wu Y-H, Liu H-Y, Luo Y, Sun M-S, et al. Prevalence of stroke and associated risk factors: a population based cross sectional study from northeast China. 2017;7(9):e015758.

63. Samadoulougou DRS, Kpoda H, Traore I, Savadogo L, Sombie I, Millogo A, editors. Evolution of the magnitude of stroke at the teaching hospital of Bobo-Dioulasso. World Congress on Clinical Trials in Diabetes; 2016: BioScientifica.

64. Ghandehari KJGMJ. Epidemiology of stroke in Iran. 2016;5(S1):3-9.

65. Farhoudi M, Mehrvar K, Sadeghi-Bazargani H, Hashemilar M, Seyedi-Vafaee M, Sadeghi-Hokmabad E, et al. Stroke subtypes, risk factors and mortality rate in northwest of Iran. 2017;16(3):112.

66. Appelros P, Stegmayr B, Terént AJS. Sex differences in stroke epidemiology: a systematic review. 2009;40(4):1082-90.

67. Yao X-y, Lin Y, Geng J-l, Sun Y-m, Chen Y, Shi G-w, et al. Age-and gender-specific prevalence of risk factors in patients with first-ever ischemic stroke in China. 2012;2012.

68. Chobanian AV, Bakris GL, Black HR, Cushman WC, Green LA, Izzo Jr JL, et al. Seventh report of the joint national committee on prevention, detection, evaluation, and treatment of high blood pressure. hypertension. 2003;42(6):1206-52.

69. Avan A, Digaleh H, Di Napoli M, Stranges S, Behrouz R, Shojaeianbabaei G, et al. Socioeconomic status and stroke incidence, prevalence, mortality, and worldwide burden: an ecological analysis from the Global Burden of Disease Study 2017. 2019;17(1):191.

70. Lawes CM, Bennett DA, Feigin VL, Rodgers AJS. Blood pressure and stroke: an overview of published reviews. 2004;35(3):776-85.

71. Bender M, Jusufovic E, Railic V, Kelava S, Tinjak S, Dzevdetbegovic D, et al. High burden of stroke risk factors in developing country: the case study of Bosnia-Herzegovina. 2017;29(4):277.

72. Watila M, Ibrahim A, Balarabe S, Gezawa I, Bakki B, Tahir A, et al. Risk factor profile among black stroke patients in Northeastern Nigeria. 2012;4(5):50-8.

73. Hossain A, Ahmed N, Rahman M, Islam M, Sadhya G, Fatema KJFMCJ. Analysis of sociodemographic and clinical factors associated with hospitalized stroke patients of Bangladesh. 2011;6(1):19-23.

74. Mills KT, Bundy JD, Kelly TN, Reed JE, Kearney PM, Reynolds K, et al. Global disparities of hypertension prevalence and control: a systematic analysis of population-based studies from 90 countries. 2016;134(6):441-50.

75. Alcohol and stroke, Factsheet13 (2014).

76. Paul E Ronksley SEB, Barbara J Turner, Kenneth J Mukamal, William A Ghali. Association of alcohol consumption with selected cardiovascular disease outcomes: a systematic review and meta-analysis. BMJ. 2011:342:d671.

77. Beyene NJJophp. Alcohol control policy in Ethiopia and implications for public health. 2019;40(4):423-35.

78. Getachew T, Defar A, Teklie H, Gonfa G, Bekele A, Bekele A, et al. Magnitude and predictors of excessive alcohol use in Ethiopia. 2017;31(1).

79. Ayata C, Shin HK, Dileköz E, Atochin DN, Kashiwagi S, Eikermann-Haerter K, et al. Hyperlipidemia disrupts cerebrovascular reflexes and worsens ischemic perfusion defect. 2013;33(6):954-62.

80. Popkin BMJHA. Will China's nutrition transition overwhelm its health care system and slow economic growth? 2008;27(4):1064-76.

81. Song P, Zha M, Yang X, Xu Y, Wang H, Fang Z, et al. Socioeconomic and geographic variations in the prevalence, awareness, treatment and control of dyslipidemia in middle-aged and older Chinese. 2019;282:57-66.

82. Xu S, Ming J, Yang C, Gao B, Wan Y, Xing Y, et al. Urban, semi-urban and rural difference in the prevalence of metabolic syndrome in Shaanxi province, northwestern China: a population-based survey. 2014;14(1):104.

83. Oguejiofor O, Onwukwe C, Odenigbo CJAoAm. Dyslipidemia in Nigeria: prevalence and pattern. 2012;11(4):197.

84. Chen R, Ovbiagele B, Feng WJTAjotms. Diabetes and stroke: epidemiology, pathophysiology, pharmaceuticals and outcomes. 2016;351(4):380-6.

85. Lekoubou A, Clovis N, Dzudie A, Kengne APJPCD. Diagnosed diabetes mellitus and in-hospital stroke mortality in a major sub-Saharan African urban medical unit. 2017;11(1):57-62.

86. Alene M, Assemie MA, Yismaw L, Ketema DBJBn. Magnitude of risk factors and in-hospital mortality of stroke in Ethiopia: a systematic review and meta-analysis. 2020;20(1):1-10.

87. Veerbeek JM, Winters C, van Wegen EE, Kwakkel GJPo. Is the proportional recovery rule applicable to the lower limb after a first-ever ischemic stroke? 2018;13(1):e0189279.

88. Hawe RL, Scott SH, Dukelow SPJS. Taking proportional out of stroke recovery. 2019;50(1):204-11.

89. Krakauer JW, Marshall RSJAon. The proportional recovery rule for stroke revisited. 2015;78(6):845-7.

90. Kjellstrom T NB, Shatchkute A. Helsingborg Declaration 2006 on European stroke strategies. Cerebrovasc Disease. 2007;23:231-41.

91. Ginenus Fekadu LC, Tsegaye Melaku, Busha Gamachu, Mohammed Gebre, Firomsa Bekele, Getahun Fetensad. Management protocols and encountered complications among stroke patients admitted to stroke unit of Jimma university medical center, Southwest Ethiopia: Prospective observational study. Annals of Medicine Surgery. 2019:135–43.

92. Sarfo FS, Akassi J, Awuah D, Adamu S, Nkyi C, Owolabi M, et al. Trends in stroke admission and mortality rates from 1983 to 2013 in central Ghana. 2015;357(1-2):240-5.

Figure Legends

Figure 1: Flow chart to a selection of studies for a systematic review and meta-analysis of the proportion of adherence to healthy lifestyle modification of people with hypertension in Ethiopia 2020.

Figure 2: ~~Frost~~ plot of in the proportion of hemorrhagic stroke in Ethiopia, 2020.

Figure 3 Forest plot of in the proportion of ischemic stroke in Ethiopia, 2020.

Figure 4: Forest plot of the proportion of recover during discharge after stroke in Ethiopia, 2020.

Figure 5: Meta funnels presentations of the proportion of recover after stroke in Ethiopia, 2020, whereby SE PIV (standard error of proportion) plotted on the Y-axis and log PIV (logarithm of proportion.

Figure 6: One-leave-out sensitivity analysis for studies conducted on the pooled estimated proportion of stroke clinical outcome in Ethiopia, 2020.

Response to Reviewers

Response to editor and reviewers'

Response to the editor:

We thank you and the reviewers for a thorough reading and constructive criticism of our manuscript and for the opportunity to revise and resubmit. We are pleased to submit the improved research article, including a proposed comment, "**The burden of stroke and modifiable risk factors in Ethiopia: a systemic review and meta-analysis with a manuscript ID of PONE-D-21-01404**"

1. General Comments:

#1. COMMENT: Please ensure that your manuscript meets PLOS ONE's style requirements, including those for file naming.

RESPONSE： We have checked and attest that all formatting and style requirements have been met PLOS ONE's style requirements.

#2. COMMENT: The funders had no role in study design, data collection and analysis, decision to publish, or preparation of the manuscript. At this time, please address the following queries…

RESPONSE: The authors received no specific funding for this work.

#3. COMMENT: Please include captions for your Supporting Information files at the end of your manuscript, and update any in-text citations to match accordingly.

RESPONSE: We include all capitation

#4. COMMENT: Thank you for submitting the above manuscript to PLOS ONE. During our internal evaluation of the manuscript, we found significant text overlap between your submission and the following previously published works, some of which you are an author.

RESPONSE: We revise the manuscript to rephrase the duplicated text.

2. Review Comments to the Author

REVIEWER #1 COMMENTS

1. **COMMENT:** There are multiple language errors, grammatical mistakes, typing errors which alter the meaning of what the authors may be trying to convey.

   RESPONSE: the language expert copyedited the manuscript for language, spelling, grammar and sentence structure.

2. **COMMENT:** There is use of different fonts in different sections of the manuscript, which the authors need to address.

RESPONSE: Thank you this comment. We make uniform of fonts throughout the manuscript.

3. **COMMENT:** The discussion section needs to highlight the reasons for the difference in results of each significant risk factor when compared to other studies quoted.

   RESPONSE: We try to highlight the reason from the different results of each significant risk factor when compared to other studies quoted.

4. **COMMENT:** The conclusion should emphasize how this study adds to literature; authors should point out feasible strategies suggested by them that can help address the modifiable risk factors in Ethiopia.

   RESPONSE: We accept the comment and we emphasize feasible strategies in the nation-Ethiopia.

REVIEWER #2 COMMENTS

1. **COMMENT**: Revise the research type as "review" not "research article"

   RESPONSE: we accept the comment and replace research article" by “review”

2. **COMMENT**: Explain the operational definition you have used regarding "alcohol consumption", its broad term, is it any alcohol consumption?

   RESPONSE: when to say alcohol consumption, more than two drinks in a day for men and more than 1 drink in a day for women

3. **COMMENT**: You have not used "AJOL: database. However, most of articles from Ethiopia were published on local and regional journals; which are indexed by AJOL. So, I believe by not including AJOL in your searching process, you may miss many articles from Ethiopia.

   RESPONSE: we included AJOL for a data bases

4. **COMMENT**: You have also included un-published articles. But how did you access this un-published articles? Because I know there are many published and un-published articles from our departments you haven't included.

   RESPONSE: we access the institutional repository (like Jimma University, Addis Ababa University). If you have an included article, please share a link and we are ready to included

5. **COMMENT**: The search key words are very few, to capture all modifiable stroke risk factor. Please explain this issue.

   RESPONSE: we select the most public health important modifiable factors

6. **COMMENT**: In the Analysis process, since the authors mentioned meta-analysis, they should present their results in "Forest plot", which they didn't included.

   RESPONSE: we try to present the result in 'Forest plot' in figure 2 to figure 4.

7. **COMMENT**: Please revise your discussion section by comparing your findings with other regions mainly focusing on points unique to this region.

   RESPONSE: we try to discuss by comparing our findings with other regions mainly focusing on points unique to this region.

8. **COMMENT**: Include your data extraction detail tables as a supplementary file for detail evaluation of your work.

   RESPONSE: we include data extraction detail tables as a supplementary file.

REVIEWER #3 COMMENTS

1. **COMMENT:** The Title: was unclear and ambiguous as one doesn't understand. So this title should be changed to (for example): Burden of stroke in Ethiopia.

   RESPONSE: we changed the tittle as recommended "Burden of stroke and modifiable risk factors in Ethiopia: a systemic review and meta-analysis."

   On abstract part

2. **COMMENT:** abstract on back ground, line 2, 3. This sentence is not correct as the contribution of modifiable risk factors to the global burden of stroke is well studied in the international literature, so they should remove the word global from the sentence and specify the sentence to be regional (in Ethiopia).

   RESPONSE: the sentence is specified in regional context-Ethiopia.

3. **COMMENT**: Back ground line 3: crucial for informing stroke prevention strategies. This sentence is wrong, they should write for establishing stroke prevention strategies and I suggest that, the language of the paper should be revised by native speaker to be more clear and informative.

   RESPONSE: we accept the comment and write as the reviewer suggestion.

4. **COMMENT:** back ground line 5: one of the keys to improving the quality of life. To improve not to improving, and as I said before the language of paper should be revised and rewritten by native speaker

   RESPONSE: we accept the comment and write as the reviewer suggestion.

5. **COMMENT:** Conclusion: needs to be rewritten, it should be more concise and informative.

   RESPONSE: we try to revised the conclusion section.
