# Supplementary material for: The burden of stroke and modifiable risk factors in Ethiopia: A systemic review and meta-analysis
Source: PLoS One. 2021 Nov 1;16(11):e0259244. doi: 10.1371/journal.pone.0259244 (PMC8559958; doi:10.1371/journal.pone.0259244)
Supplement: Attachment — Submitted filename: Response to Reviewers.docx [file pone.0259244.s008.docx]

**Response to Reviewers**

Response to editor and reviewers’
Response to the editor:

We thank you and the reviewers for a thorough reading and constructive criticism of our manuscript and for the opportunity to revise and resubmit. We are pleased to submit the improved research article, including a proposed comment, “**The burden of stroke and modifiable risk factors in Ethiopia: a systemic review and meta-analysis with a manuscript ID of PONE-D-21-01404**”

1. General Comments:

**#1. COMMENT**: Please ensure that your manuscript meets PLOS ONE's style requirements, including those for file naming.

RESPONSE: We have checked and attest that all formatting and style requirements have
been met PLOS ONE's style requirements.

#2. COMMENT: The funders had no role in study design, data collection and analysis, decision to publish, or preparation of the manuscript. At this time, please address the following queries…

RESPONSE: The authors received no specific funding for this work.

#3. COMMENT: Please include captions for your Supporting Information files at the end of your manuscript, and update any in-text citations to match accordingly.

**RESPONSE: We include all capitation**

#4. COMMENT: Thank you for submitting the above manuscript to PLOS ONE. During our internal evaluation of the manuscript, we found significant text overlap between your submission and the following previously published works, some of which you are an author.

RESPONSE: We revise the manuscript to rephrase the duplicated text.

2. **Review Comments to the Author**

**REVIEWER #1 COMMENTS**

1. **COMMENT:** There are multiple language errors, grammatical mistakes, typing errors which alter the meaning of what the authors may be trying to convey.

RESPONSE: the language expert copyedited the manuscript for language, spelling, grammar and sentence structure.

2. COMMENT: There is use of different fonts in different sections of the manuscript, which the authors need to address.

**RESPONSE: Thank you this comment. We make uniform of fonts throughout the manuscript.**

3. **COMMENT:** The discussion section needs to highlight the reasons for the difference in results of each significant risk factor when compared to other studies quoted.

**RESPONSE: We try to highlight the reason from the different results of each significant risk factor when compared to other studies quoted.**

4. **COMMENT:** The conclusion should emphasize how this study adds to literature; authors should point out feasible strategies suggested by them that can help address the modifiable risk factors in Ethiopia.

**RESPONSE: We accept the comment and we emphasize feasible strategies in the nation- Ethiopia.**

**REVIEWER #2 COMMENTS**

1. **COMMENT**: Revise the research type as "review" not "research article"

**RESPONSE: we accept the comment and replace** research article" by “review”

2. **COMMENT**: Explain the operational definition you have used regarding "alcohol consumption", its broad term, is it any alcohol consumption?

**RESPONSE: when to say alcohol consumption, more than two drinks in a day for men and more than 1 drink in a day for women**

3. **COMMENT**: You have not used "AJOL: database. However, most of articles from Ethiopia were published on local and regional journals; which are indexed by AJOL. So, I believe by not including AJOL in your searching process, you may miss many articles from Ethiopia.

**RESPONSE: we included AJOL for a data bases**

4. **COMMENT**: You have also included un-published articles. But how did you access this un-published articles? Because I know there are many published and un-published articles from our departments you haven't included.

**RESPONSE: we access the institutional repository (like Jimma University, Addis Ababa University). If you have an included article, please share a link and we are ready to included**

5. **COMMENT**: The search key words are very few, to capture all modifiable stroke risk factor. Please explain this issue.

**RESPONSE: we select the most public health important** **modifiable factors**

6. **COMMENT**: In the Analysis process, since the authors mentioned meta-analysis, they should present their results in "Forest plot", which they didn't included.

**RESPONSE: we try to present the result in ‘Forest plot’ in figure 2 to figure 4.**

7. **COMMENT**: Please revise your discussion section by comparing your findings with other regions mainly focusing on points unique to this region.

**RESPONSE: we try to discuss by comparing our findings with other regions mainly focusing on points unique to this region.**

8. **COMMENT**: Include your data extraction detail tables as a supplementary file for detail evaluation of your work.

**RESPONSE: we include data extraction detail tables as a supplementary file.**

**REVIEWER #3 COMMENTS**

1. **COMMENT:** The Title: was unclear and ambiguous as one doesn’t understand. So this title should be changed to (for example): Burden of stroke in Ethiopia.

**RESPONSE:** **we** **changed the tittle as recommended “Burden of stroke and modifiable risk factors in Ethiopia: a systemic review and meta-analysis**.”

**On abstract part**

2. **COMMENT:** abstract on back ground, line 2, 3. This sentence is not correct as the contribution of modifiable risk factors to the global burden of stroke is well studied in the international literature, so they should remove the word global from the sentence and specify the sentence to be regional (in Ethiopia).

**RESPONSE: the sentence is specified in regional context-Ethiopia.**

3. **COMMENT**: Back ground line 3: crucial for informing stroke prevention strategies. This sentence is wrong, they should write for establishing stroke prevention strategies and I suggest that, the language of the paper should be revised by native speaker to be more clear and informative.

**RESPONSE: we accept the comment and write as the reviewer suggestion.**

4. **COMMENT:** back ground line 5: one of the keys to improving the quality of life. To improve not to improving, and as I said before the language of paper should be revised and rewritten by native speaker

**RESPONSE: we accept the comment and write as the reviewer suggestion.**

5. **COMMENT:** Conclusion: needs to be rewritten, it should be more concise and informative.

**RESPONSE: we try to revised the conclusion section.**
