# Supplementary material for: The burden of stroke and modifiable risk factors in Ethiopia: A systemic review and meta-analysis
Source: PLoS One. 2021 Nov 1;16(11):e0259244. doi: 10.1371/journal.pone.0259244 (PMC8559958; doi:10.1371/journal.pone.0259244)
Supplement: Attachment — Submitted filename: Response to Reviewers.docx [file pone.0259244.s010.docx]

Response to Reviewers

Response to editor and reviewer
we thank you and the reviewers for a thorough reading and constructive criticism of our manuscript and for the opportunity to revise and resubmit. We are pleased to submit the improved research article, including a proposed comment, “**The burden of stroke and modifiable risk factors in Ethiopia: a systemic review and meta-analysis.**”

**RESPONSE TO REVIEWER 1**

REVIEWER #1 COMMENT:

1. Although the content of the paper is good, however the language errors and grammatical mistakes in the document are still far too many to consider this as a meaningful submission. The typing errors, missing verbs and punctuations completely take away from what the authors are trying to convey. I strongly recommend that they take the help of an English language expert to thoroughly revise the whole manuscript before submission.

**RESPONSE:** we try to address the comment and one fried who is a language expert help me in editorial technique.

2. In my previous review I had submitted an attachment with many comments and edits (apart from comments), however those have not been addressed at all. I urge the authors to go through the attachments submitted along with the comments as well before submitting their revisions.

**RESPONSE:** we addressed all the important comment in the revised manuscript.

3. The references are not in the correct format and there is no uniformity. Authors’ names as well as journal names are missing. Authors need to re-write this whole section and present the bibliography correctly.

**RESPONSE: we intensive edit the whole reference by using endnote reference manager software.**

4. . I would like to re-iterate that the authenticity and content of a paper are just as important as the presentation, for acceptance to any journal. I request the authors to thoroughly go through the document again and make relevant edits before submission.

**RESPONSE: we try to thoroughly edit and incorporate all the comment of the reviewer**
