# Supplementary material for: The burden of stroke and modifiable risk factors in Ethiopia: A systemic review and meta-analysis
Source: PLoS One. 2021 Nov 1;16(11):e0259244. doi: 10.1371/journal.pone.0259244 (PMC8559958; doi:10.1371/journal.pone.0259244)
Supplement: Attachment — Submitted filename: Response to Reviewers.docx [file pone.0259244.s012.docx]

Response to Reviewers

Response to editor and reviewer
We thank you and the reviewers for a thorough reading and constructive criticism of our manuscript and for the opportunity to revise and resubmit. We are pleased to submit the improved research article, including a proposed comment, “**The burden of stroke and modifiable risk factors in Ethiopia: a systemic review and meta-analysis.**”

**Response to editorial comment**

**Comment:** Journal Requirements: Please review your reference list to ensure that it is complete and correct. If you have cited papers that have been retracted, please include the rationale for doing so in the manuscript text, or remove these references and replace them with relevant current references. Any changes to the reference list should be mentioned in the rebuttal letter that accompanies your revised manuscript. If you need to cite a retracted article, indicate the article’s retracted status in the References list and also include a citation and full reference for the retraction notice.

**RESPONSE**: **We revised the reference list a cording to journal requirements. *Reference ’75: Alcohol and stroke, Factsheet13 (2014)*. ‘ with ‘Zhang C, Qin Y-Y, Chen Q, Jiang H, Chen X-Z, Xu C-L, et al. Alcohol intake and risk of stroke: a dose–response meta-analysis of prospective studies. International journal of cardiology. 2014;174’ the reason of replacement, the update one more appropriate one.**(669-677)’ b

**RESPONSE TO REVIEWER 1**

REVIEWER #1 COMMENT:

1. The references are however not according to format. Journal abbreviations need to be formatted.

**RESPONSE**: we used reference manager software (EndNote) and mange according to this software manger.

2. Few other minor errors are present, which I have addressed in the attachment.

**RESPONSE: we addressed all comment, which we have found in the attachment comments.**
