# Supplementary material for: The burden of stroke and modifiable risk factors in Ethiopia: A systemic review and meta-analysis
Source: PLoS One. 2021 Nov 1;16(11):e0259244. doi: 10.1371/journal.pone.0259244 (PMC8559958; doi:10.1371/journal.pone.0259244)
Supplement: Attachment — Submitted filename: Response to Reviewers.docx [file pone.0259244.s013.docx]

Response to editor and reviewer

We thank you and the reviewers for a thorough reading and constructive criticism of our manuscript and for the opportunity to revise and resubmit. We are pleased to submit the improved research article, including a proposed comment, “**The burden of stroke and modifiable risk factors in Ethiopia: a systemic review and meta-analysis.**”

**RESPONSE TO EDITORIAL COMMENT AND RESPONSE TO REVIEWER 1**

EDITORIAL/REVIEWER #1 COMMENT:

1. Please review your reference list to ensure that it is complete and correct. If you have cited papers that have been retracted, please include the rationale for doing so in the manuscript text, or remove these references and replace them with relevant current references. Any changes to the reference list should be mentioned in the rebuttal letter that accompanies your revised manuscript. If you need to cite a retracted article, indicate the article’s retracted status in the References list and also include a citation and full reference for the retraction notice.

**RESPONSE**: **we used reference manager software (EndNote) and mange according to this software manger. And also rewrite with ‘NLM abbreviation forma’**

2. Please edit your references. The journal names should be in NLM abbreviated format.

**RESPONSE: we try to edit all reference in NLM abbreviation format.**

3. Please upload a Response to Reviewers letter which should include a point by point response to each of the points made by the Editor and / or Reviewers. (This should be uploaded as a 'Response to Reviewers' file type.)

**RESPONSE: we uploaded the response letter.**

4. Please include captions for your Supporting Information files at the end of your manuscript, and update any in-text citations to match accordingly.

**RESPONSE: we include caption of the supporting information file which are cited in the manuscript. But, supportive information which are not cited in the manuscript are not caption at the end of the manuscript. Such as appendix 3, data extraction speed sheet and PRISMA check list.**
